# Habitual consumption of high-fibre bread fortified with bean hulls increased plasma indole-3-propionic concentration and decreased putrescine and deoxycholic acid faecal concentrations in healthy volunteers

**DOI:** 10.1017/S0007114523000491

**Published:** 2023-11-14

**Authors:** Marietta Sayegh, Qian Qian Ni, Viren Ranawana, Vassilios Raikos, Nicholas J. Hayward, Helen E. Hayes, Gary Duncan, Louise Cantlay, Freda Farquharson, Michael Solvang, Graham W. Horgan, Petra Louis, Wendy R. Russell, Miriam Clegg, Frank Thies, Madalina Neacsu

**Affiliations:** 1 The Rowett Institute, University of Aberdeen, Aberdeen AB25 2ZD, UK; 2 BIOSS Aberdeen, Aberdeen, UK; 3 Institute for Food, Nutrition and Health and Department of Food and Nutritional Sciences, University of Reading, Whiteknights, UK

**Keywords:** Dietary fibre, Bean hulls, Food fortification, Agricultural by-products, High-fibre bread, Indole 3-propionic acid

## Abstract

Only 6 to 8 % of the UK adults meet the daily recommendation for dietary fibre. Fava bean processing lead to vast amounts of high-fibre by-products such as hulls. Bean hull fortified bread was formulated to increase and diversify dietary fibre while reducing waste. This study assessed the bean hull: suitability as a source of dietary fibre; the systemic and microbial metabolism of its components and postprandial events following bean hull bread rolls. Nine healthy participants (53·9 ± 16·7 years) were recruited for a randomised controlled crossover study attending two 3 days intervention sessions, involving the consumption of two bread rolls per day (control or bean hull rolls). Blood and faecal samples were collected before and after each session and analysed for systemic and microbial metabolites of bread roll components using targeted LC-MS/MS and GC analysis. Satiety, gut hormones, glucose, insulin and gastric emptying biomarkers were also measured. Two bean hull rolls provided over 85 % of the daily recommendation for dietary fibre; but despite being a rich source of plant metabolites (*P* = 0·04 *v*. control bread), these had poor systemic bioavailability. Consumption of bean hull rolls for 3 days significantly increased plasma concentration of indole-3-propionic acid (*P* = 0·009) and decreased faecal concentration of putrescine (*P* = 0·035) and deoxycholic acid (*P* = 0·046). However, it had no effect on postprandial plasma gut hormones, bacterial composition and faecal short chain fatty acids amount. Therefore, bean hulls require further processing to improve their bioactives systemic availability and fibre fermentation.

## Graphical abstract

**Figure:**
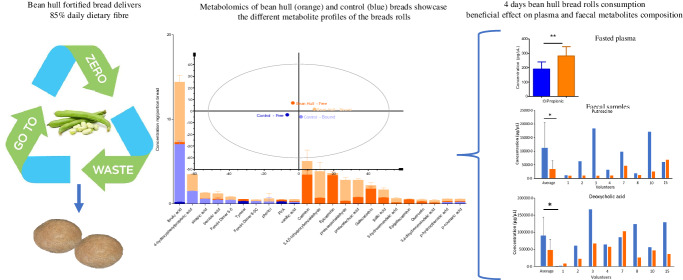

The Scientific Advisory Committee on Nutrition (SACN) advised daily fibre intakes of 30 g for adults (with proportionally lower recommendations for children from the age of 2 years)^(1)^. However, the latest UK National Diet and Nutrition Survey (NDNS) shows UK fibre intake was below the government recommendations for all older age/sex groups, with only 9 % of adults meeting recommended intakes^(2)^. The UK’s primary sources of dietary fibre are cereals and other grains ranging from 38 % to 44 % of intake across the age groups, followed by vegetables and potatoes (21 % to 32 %) and fruit (6 % to 16 %)^(2)^.

It is well recognised that a diet rich in fibre contributes to maintaining a healthy gut microbiota associated with increased diversity and functions, such as the production of SCFA in the colon, mainly acetate, propionate and butyrate^(3)^. Butyrate is a primary energy source for the enterocytes and also modulates immune activity, while acetate and propionate mainly exert systemic regulatory functions^(3)^. Microbial SCFA exert several beneficial effects on human metabolism by intervening in glucose homeostasis, lipid metabolism and appetite regulation^(4)^. Increased SCFA concentrations may also increase the solubility of certain minerals in the gut^(5)^. In addition, diversification of dietary fibre could contribute to beneficial health effects through the increase of gut microbial diversity^(6,7)^. Moreover, it has been shown that a diet high in fibre intake and whole grain cereals promotes the synthesis of indole-3-propionic acid, a microbial-derived metabolite of tryptophan, associated with lower likelihood of type 2 diabetes^(8)^, and delivers substantially higher bound phytophenols than commonly consumed fruits and vegetables^(9)^.

The current UK food supply does not have sufficient dietary fibre for everyone to meet the 30 g/d recommendation^(10^, therefore is vital to identify novel sources of dietary fibre. According to the FAO, approximately one-third of all food produced for human consumption in the world is either lost or wasted^(11)^. A significant volume of secondary crop products such as peels, hulls and leaves that are generated during processing remain underutilised. Valorising this type of material represents an opportunity to reduce food waste while delivering foods rich in quality nutrients such as fibre protein and bioactive phytochemicals.

Broad bean (*Vicia faba*; field bean, horse bean, faba bean) is a legume belonging to the family Fabaceae. It is a major dietary component in many parts of the world, notably in Middle Eastern, Mediterranean, Asian, South American and African regions, and the UK is the largest producer of broad beans in Europe, with a cultivation area of approximately 170 000 ha, with most being grown for export^(12)^. The main part of the plant used for food is the seed consumed fresh or dried, which has a favourable nutritional profile rich in protein, fibre, micronutrients and phenols, beneficial for human health^(13–15)^. The processing of broad beans involves the removal of the seed testa (hull), which constitutes approximately 12–14 % of dry seed weight^(16)^. Therefore, the hull is an important by-product of bean processing which is largely discarded. To date, little work has been carried out to assess the potential of broad bean hulls for human consumption despite being a rich source of fibre and phytochemicals^(14)^. To fully comprehend the bean hull as a potential source of food, it is essential to assess the bioavailability and metabolism of its nutrients and bioactive molecules at the systemic and gut levels. This will also inform on hull potential biological activity and will advise further on the potential for food formulation.

Therefore, this study assessed the bean hulls’ suitability to be used as a source of dietary fibre in order to help contribute to dietary recommendations and promote diversity while contributing to agricultural waste reduction.

## Materials and methods

### Volunteer recruitment

Nine healthy volunteers (four men and five women, age 54·1 ± 16·9, BMI: 25·6 ± 2·0 kg/m^2^) were recruited. Inclusion criteria included healthy males and females aged 18–75 years, BMI in the range of 23–35 kg/m^2^, HbA1c < 6·5 %, total cholesterol ≤ 7 mmol/l < 5 mmol/l, systolic blood pressure (BP) ≤ 139 mmHg and diastolic BP ≤ 89 mmHg, with no chronic health conditions. Individuals with glucose 6 phosphate dehydrogenase deficiency, smokers, athletes, those with food intolerances/allergies, women with polycystic ovaries syndrome, pregnant or breastfeeding, using prescription medication including hormonal contraceptives, thyroid medications or hormonal replacement therapy were excluded. Additionally, volunteers who habitually consumed ≥ 3 portions of fruits and vegetables per day or had taken antibiotics in the last 4 weeks before taking part in the study were also excluded.

Prospective volunteers who registered an interest in the study signed the consent form and attended a screening visit at the Human Nutrition Unit (HNU) at the Rowett Institute. The volunteers completed a general health questionnaire, and their height and weight were measured in the fasted state. A fasting blood sample was also collected to assess glucose 6 phosphate dehydrogenase deficiency deficiency, performed at Aberdeen Royal Infirmary Hospital in Foresterhill. Eligible volunteers participated in two randomised cross-over intervention visits to the HNU, each lasting 3 days and separated by a minimum washout period of 1 week.

### Study design

Before one of the two intervention sessions, the volunteers recorded their habitual diet for seven consecutive days in food diaries. The evening before each visit, volunteers were provided with a standardised meal consisting of a vegetarian paella (made in-house), a glass of semi-skimmed milk, toffee yoghurt and a plain digestive biscuit. Supplementary Table 1 provides the details on nutritional information for this meal.

**Fig. 1.**
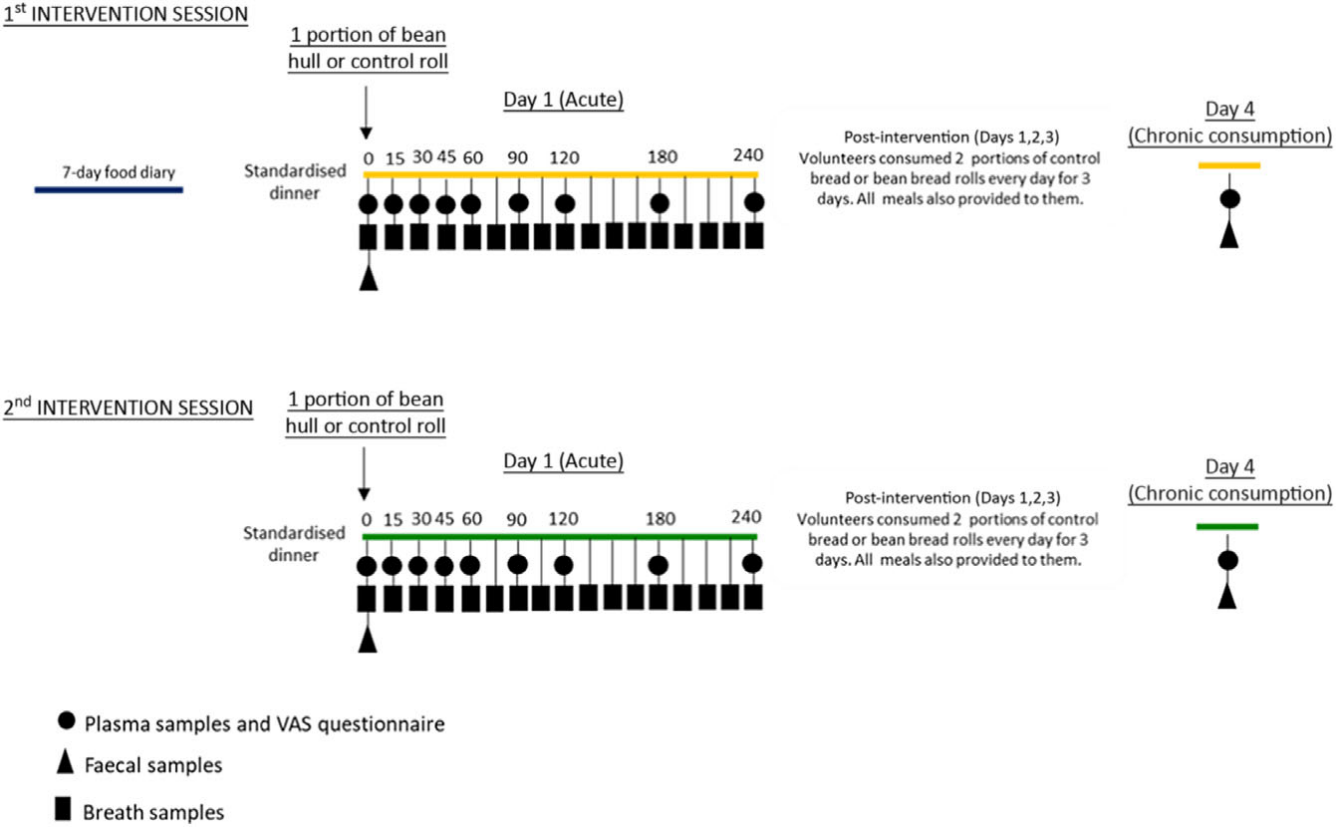
Human dietary intervention study diagram. Each volunteer (*n* 9) consumed in random order either a portion of bean hull bread roll (one roll, in average 155·8 g) or a control bread roll (one roll, in average 122·3 g) along with a 25 g of spread following a standardised dinner the evening before. Sample collection: at baseline (0 h), fasted blood and faecal samples; following meal consumption, over 4 hours blood samples together with VAS questionnaires were taken at time points described in diagram, and breath samples were collected every 15 min. For the remaining of day 1 and for days 2 and 3, volunteers were provided with all meals and with two portions of control bread or bean hull bread rolls per day. In the morning of day 4 (after chronic consumption of a total of six control or bean hull bread rolls), fasted blood and faecal samples were collected. A 1-week food diary was completed once before volunteers’ first intervention session. Between the two intervention sessions, there was a minimum washout period of 7 days. VAS, Visual Analogue Scale questionnaire.

On the morning of the day of each intervention session, the volunteers arrived and fasted at the HNU, and their fat mass was measured using BodPod® (COSMED Srl). Their blood pressure was also measured using a blood pressure monitor (OMRON Healthcare). Participants consumed the test meal within 10 min, and their blood samples (45 ml in total) were collected using a cannula at baseline (before the test meal consumption) and at 15, 30, 45, 60, 90, 120, 180 and 240 min from the start of the meal (see intervention day diagram, Fig. 1). The volunteers completed a satiety questionnaire based on 100 mm visual analogue scales at each time point. Breath samples were collected at baseline and every 15 min for 4 h after the meal consumption to measure gastric emptying. For this purpose, 100 mg of sodium (1–^13^C) acetate was added to the test meal at the time of baking.

Blood glucose levels were measured for 240 min postprandially using a Continuous Glucose Monitoring system (iPro2, Medtronic MiniMed), which determined interstitial glucose concentrations every 5 min. Faecal samples were collected before the beginning of the intervention (see intervention day diagram, Fig. 1) and on the morning of the fourth day, along with a fasted blood sample. At the end of the morning of each intervention visit, volunteers were provided with meals for the rest of the day and the subsequent 2 days. Participants were only instructed to consume only the meals that were provided to them during the study days. These meals included two portions per day of either the bean hull or control bread rolls; each participant consumed six bread rolls in total, spread over 3 days. The meals were standardised for energy and macronutrients (online Supplementary Table 2) based on the participants’ gender (daily energy standardised to 8·368 MJ and 10·46 MJ for women and men, respectively). To check for the volunteers’ compliance, the participants were asked to complete a food diary during these three study days.

The study was conducted in accordance with the Declaration of Helsinki, and all procedures involving human subjects were reviewed and approved by the Human Studies Management Committee of the Rowett Institute, University of Aberdeen, UK, and the Rowett Institute Ethics Panel and registered with clinicaltrial.gov (study ID number: NCT05252013). Participants were recruited between January 2019 and March 2020.

### Test meals

The preparation of the control and the bean hull bread rolls has been described previously^(17)^. One roll of control and bean hull bread was equivalent to one portion. Each control bread roll was prepared using only wheat flour (75 g) (Allison white bread flour, Tesco). The bean hull bread roll contained additionally 20 g of broad bean hull powder. The water content and salt were adjusted accordingly. The broad bean hull (variety ‘Fuego’) used for the bread rolls was kindly provided by Askew and Barrett (Pulses) Ltd. A detailed description of the ingredients and compositions of the bread rolls can be found in Supplementary Table 3. On each test day, each bread roll (on average 122·3 g control and 155·8 g bean hull) was served with 25 g of raspberry jam (Tesco Stores Ltd.). In addition, a standardised lunch containing 15 % protein, 30 % fat and 55 % carbohydrate was provided 4 hours after the test meal (online Supplementary Table 2).

### General reagents

Standards and general laboratory reagents were purchased from Sigma-Aldrich (Gillingham, England) and Fisher Scientific UK Ltd or synthesised as described previously^(18,19)^. Chemicals used for ICP-MS analysis were nitric acid of TraceSelect Ultra grade (Fluka, Sigma-Aldrich), hydrochloric acid (30 %) of Ultrapur grade (Merck), and deionised water (Millipore). Single elements standards were purchased from all Inorganic Ventures. Sulphatase (*Helix Pomatia* - S9626) and glucuronidase (*Helix Pomatia* - G7017) were purchased from Merck. The internal standards for LC-MS were 2-amino-3,4,7,8-tetramethyl-3H-imidazo [4,5-f] quinoxaline (4,7,8-tri-MelQx) from Toronto Research Chemicals Inc. and 13C-benzoic acid from Isotec, Sigma-Aldrich.

### Macronutrient and micronutrients composition of bread rolls

Macronutrients (protein, fibre measured as soluble and insoluble NSP and fat) were measured as previously described^(15)^. Briefly, the protein content was measured as total nitrogen by the Dumas combustion method, using a conversion factor of 6·25 to calculate the protein concentration. Resistant starch and NSP were measured according to the methods described by Englyst et al. (1992)^(20)^. Total fat was determined by Soxhlet extraction using Soxtec 2050 Auto Fat Extraction System^(15)^.

Micronutrient analysis was conducted as previously described^(15)^ using Inductively Coupled MS (ICP-MS) (Agilent 7700X spectrometer, Agilent Technologies), equipped with a MicroMist nebulizer, nickel sampler and skimmer cones and erbium as internal standard.

### Phytic acid content analysis of the bread rolls

The phytic acid content of the samples was measured and calculated using the Phytic Acid Assay Kit (Megazyme), as described previously^(21)^.

### Determination of *in vitro* fermentability of the bean hull fibre

Anaerobic incubations were carried out in Hungate tubes as described previously^(22)^. Fresh faecal samples were obtained from three different donors (2 females, 1 male; age 33–59) with no history of gastrointestinal disorders or antibiotic treatment for at least 3 months prior to the study and processed within 2–3 h after defecation. Eight millilitres of anaerobic PBS (Sigma-Aldrich) were added to 2 g of faecal sample and homogenised in a gentleMACS C tube (Miltenyi Biotech) on a Dispomix Drive (Medic Tools). The homogenate was further diluted 5-fold in anaerobic PBS and 0·5 ml of the homogenised faecal suspension was used as an inoculum for 9·5 ml of fermentation medium^(22)^, set to pH 6, to achieve a final faecal concentration of 0·2 %. Four different substrate settings were compared: (1) no added substrate (no carbohydrate control, ‘NO CHO’); (2) 0·6 % (w/v) alcohol-insoluble residue (AIR) of bean hull powder; (3) 0·6 % (w/v) of a mix of different carbohydrates to simulate the major components of plant cell walls (recombined cell wall, ‘RCW’), consisting of 33·3 % cellulose (11 365, Sigma Aldrich), 33·3 % apple pectin (76 282, Sigma Aldrich), 22·1 % xyloglucan (P-XYGLN, Megazyme), 9·1 % xylan (P-XYLNBE, Megazyme) and 2·1 % mannan (P-MANCB, Megazyme); (4) 0·2 % inulin (Sigma Aldrich I2255). Bean hull AIR was prepared by stirring the powder in 70 % ethanol (33·3 % [w/v]) at room temperature for 3 days, replacing the 70 % ethanol in the morning, noon and late afternoon by centrifugation (3000 g; Eppendorf 5810R) and removal of the supernatant. The AIR was freeze-dried (Labconco) and stored at room temperature. All substrates were sterilised in absolute ethanol and dried under a flow of nitrogen. Incubations were carried out in triplicate at ∼90° angle on a rotator (Stuart SB3, Bibby Scientific) at 25 rpm, 37°C. Gas production was measured after 24, 48 and 72 h with a glass syringe by penetrating the rubber seal of the Hungate tube with a sterile needle and recording the displayed volume. Culture aliquots (1·5 ml) were removed from the incubation tubes under CO_2_ at 24 and 48 h. The aliquots were centrifuged (10 000 × g; 10 min; 4°C; miniSpin, Eppendorf), and the supernatant was collected for SCFA analysis. The pellet was resuspended in sodium phosphate buffer (978 µl) and MT buffer (122 µl), both part of the FastDNA spin kit for soil (MP Biomedicals), and stored at −70°C until DNA extraction. On the day of inoculation (T = 0 h), aliquots of the faecal inocula (500 µl) were added to lysis matrix tubes together with sodium phosphate buffer (478 µl) and MT buffer (122 µl) and stored at –70°C until DNA extraction.

### Extraction of plant metabolites from bread rolls

The bread rolls were freeze-dried then freeze-milled in liquid nitrogen (Spex 6700; Edison). Plant metabolites were extracted as previously described^(9)^. Briefly, samples (approximately 0·1 g dry weight; *n* 3) were suspended in hydrochloric acid extracted into ethyl acetate. The resultant pellet was sequentially hydrolysed and extracted for the acid and alkali-labile phenolic compounds. The extracts were then reconstituted in methanol and prepared for LC-MS analysis as described below.

### Human sample processing

The blood sample at each time point was collected directly into 5 ml Aprotinin K_3_EDTA tubes. The samples were centrifuged immediately at 3000 rpm and 4°C for 15 min to separate the plasma. After collection, the faecal samples were manually homogenised and weighed, and the faecal waters were separated by centrifugation (50 000 rpm × g; 2 h; 4°C) as previously described^(23)^. During the baseline visit, fasting total cholesterol and glycated HbA1c were quantified in plasma samples using the Alere Cholestech LDX (Alere) and the Afinion AS100 analyser (Alere), respectively. Body fat composition was collected using BodPod®.

### Samples analysis

#### Plasma and faecal metabolites

Plasma (baseline, 30, 60, 120, 180 and 240 min and the fasted sample from day 4) and faecal samples (days 1 and 4) were processed as previously described^(23)^ and analysed using LC-MS/MS as described below.

#### SCFA analysis

SCFA concentrations were measured in volunteers’ faecal samples and in *in vitro* culture supernatants using GC as described previously^(24,25)^. After derivatisation, 1 μl of the sample was analysed using a Hewlett-Packard gas chromatograph fitted with a silica capillary column with helium as a carrier gas. The SCFA concentrations were calculated from the relative response factor with respect to the internal standard 2-ethylbutyrate^(22)^.

#### DNA extractions and quantitative PCR

DNA extractions were performed with a FastDNA spin kit for soil (MP Biomedicals) according to the manufacturer’s instructions. DNA concentrations were measured using Qubit 2·0 Fluorometer (Thermo Fisher Scientific). The total number of 16S rRNA gene copies per ml and abundance of several bacterial genera or species of the communities in the anaerobic faecal incubation experiments was determined by quantitative PCR as described previously^(22)^ with 2 ng DNA in a total volume of 10 µl and expressed as 16S rRNA gene copies per ml of culture. For the quantification of *Faecalibacterium prausnitzii*, primers used are described in Lindstad et al. (2021)^(26)^ and for the quantification of bacteria related to the *Roseburia* genus primers used were Rrec2 described in Ramirez-Farias et al. (2008)^(27)^.

#### Metabolic profile

Total PYY (peptide YY), total ghrelin, total glucagon and total glucagon-like peptide 1 (GLP-1) were analysed from postprandial plasma samples at baseline, 15, 30, 45, 60, 90, 120, 180 and 240 min following the bean hull and control bread rolls using Human Metabolic Hormone Magnetic Bead Panel (Merck Millipore) as previously described^(28)^. Plasma insulin (baseline, 15, 30, 45, 60, 90, 120, 180 and 240 min), glucose and lipid profile (baseline, 60, 120, 240 min) were measured and analysed as described before^(29)^.

#### Breath samples

Breath samples were collected at baseline (0 h) and every 15 min for 240 min and were analysed by MS. At each time point, five breath tubes were collected by the participants, and four of these were analysed using Elementar iso FLOW coupled to an isoprime precisION MS (Elementar). Gastric emptying rate analysis was completed by the analytical facilities based at the Rowett Institute.

#### The LC-MS/MS analysis

Bread plant metabolites were analysed as described previously^(15,30)^. One volunteer did not provide a baseline (day 1) faecal sample, and therefore only eight faecal samples were analysed. The detailed LC-MS/MS analysis for the plasma and faecal metabolites has been described previously^(23)^.

### Statistical analysis

The samples size of nine volunteers was predicted to detect a treatment effect 1·1 times the magnitude of the within-volunteer variation in the difference, with 80 % power at the 5 % significance level^(30)^. For glucose and insulin, the within-volunteer spread was approximately 10 % (based on previous studies), which gave the study power to detect a treatment shift of ca. 14 %^(30)^. The effect of treatment (bread rolls) over time (plasma and faecal metabolites) and between the two intervention sessions were assessed using a paired *t* test (significance level *P* ≤ 0·05). The same test was also used to determine any significant changes in glucose and insulin levels on day 4 of the intervention. All variables were analysed by ANOVA with terms for volunteer and treatment. To compare the gut microbiota composition following the bean hull and control bread rolls, ANOVA with terms for volunteer, baseline and diet was used. Glucose and insulin analysis were performed after calculating the AUC and incremental AUC of data collected over multiple time points following consumption of the test meals. Analysis was performed on log-transformed data if data distribution was skewed. The plant (non-nutrient) metabolites from the bread meals, plasma and faecal phenylpropanoid pathway and products of protein and carbohydrate metabolism data were analysed by principal component analysis (PCA) using SIMCA 14·1 (Umetrics, Cambridge). The correlation between plasma and faecal metabolites and gastrointestinal hormones was analysed by partial least squares-discriminant analysis (PLS-DA) SIMCA 14·1 (Umetrics). Unless otherwise stated, all data are presented as mean ± sd.

## Results

### Human volunteer baseline characteristics

The baseline characteristics and body composition of the participants are summarised in Table 1. Nine participants (four males and five females) with a mean age of 54·1 ± 16·9 years and BMI of 25·6 ± 2·0 kg/m^2^ were recruited to the study.

**Table 1.** Baseline characteristics and body composition of the participants

Characteristics	Values	
	Mean	sd
Males/females	4/5	
Age (years)	53·9	16·7
Height (m)	1·7	0·1
Weight (kg)	74·4	11·6
BMI (kg/m^2^)	25·6	2·0
Systolic (mmHg)	119·6	19·2
Diastolic (mmHg)	74·9	7·3
Total cholesterol (mmol/l)	5·9	1·0
HbA1c (%)	5·2	0·3
Body fat (%)	28·9	10·3

All values are mean ± sd.

### Composition of the bread rolls

#### Macronutrient content

Two bean hull bread rolls provide over 85 % of daily fibre recommendation: The monosaccharide composition of the soluble and insoluble NSP for each portion of the control and bean hull bread roll is shown in Table 2. Bean hull bread roll was rich in dietary fibre, containing over 14 % fibre (as a sum of soluble and insoluble NSP). Soluble NSP was mainly comprised of xylose and arabinose for both bean hull and control bread rolls. For the insoluble NSP composition, glucose was the main component for both bread rolls, followed by xylose and uronic acid (Table 2). Consumption of two bean hull bread rolls per day contributes to over 85 % of the recommended dietary fibre (DF) per day, more than three times higher than the DF provided by the control bread roll. The total carbohydrate, starch, resistant starch, fat, protein and free sugar contents of the two bread rolls were very similar (online Supplementary Figures 1 and 2).

**Table 2.** Monosaccharide composition of soluble non-starch polysaccharides (NSP) and insoluble NSP content, expressed as % of dry weight ± sd (*n* 4), bean hull bread roll and control bread roll

	Bean hull bread roll (% dry weight)				Control bread roll (% dry weight)			
	Insoluble NSP		Soluble NSP		Insoluble NSP		Soluble NSP	
	% of dry weight	sd	% of dry weight	sd	% of dry weight	sd	% of dry weight	sd
Rhamnose	0·101	0·006	0	0	0·005	0·006	0	0
Fucose	0·029	0·002	0·028	0·002	0·019	0·004	0·03	0·006
Arabinose	0·502	0·028	0·254	0·016	0·475	0·089	0·198	0·065
Xylose	2·257	0·125	0·462	0·049	0·733	0·137	0·31	0·123
Mannose	0·315	0·034	0·153	0·011	0·386	0·072	0·16	0·03
Galactose	0·192	0·014	0·031	0·006	0·073	0·014	0·013	0·016
Glucose	7·665	0·453	0·051	0·017	2·59	0·482	0·016	0·019
Uronic acid	2·021	0·148	0·07	0·004	0·016	0·016	0	0
Total NSP g/portion (one bread roll)	12·84 ± 0·74				3·63 ± 0·75			
Contribution to daily DF (%) (two bread rolls)*	85·6 %				24·2 %			

* Contribution to dietary fibre (DF) calculated according to SACN 2015 guidelines (recommending 30 g DF/day).

#### Micronutrient content

**Fig. 2.**
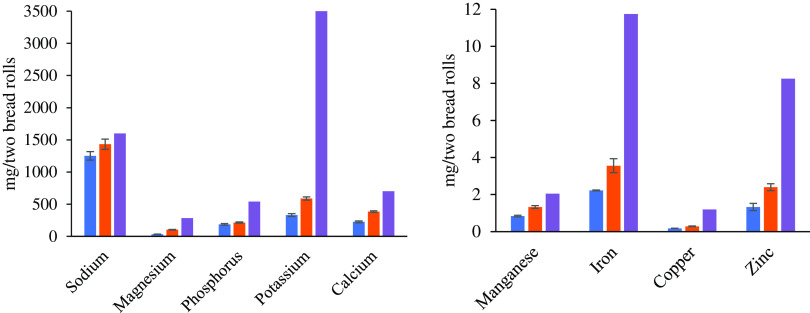
Main micronutrients expressed in mg ± sd (*n* 3) measured in control (blue) and bean hull (orange) bread rolls samples using quantitative ICP-MS analysis. Purple colour shows recommended nutrient intake (RNI) for each of the microelements (Public Health England, 2016).

Two bean hull bread rolls contribute towards daily micronutrient recommendations: bean hull bread roll was also a rich source of several minerals. Overall, the micronutrient content in the bean hull bread roll was higher than the control bread roll. Bean hull bread roll had a significantly higher content of P (*P* = 0·04), Mg (*P* < 0·001), K (*P* < 0·001), Ca (*P* < 0·001), Mn (*P* < 0·001), Fe (*P* < 0·001), Zn (*P* < 0·001) and Cu (*P* < 0·001) (Fig. 2). The consumption of two bean hull bread rolls delivers 36 % of daily recommendations of Mg, 39 % of P, approximately 55 % of Ca, 65 % of Mn, 30 % of Fe, 29 % of Zn, 24 % of Cu, 17 % of K, 15 % of Mo and approximately 10 % of Se for adult men and women. Two bean hull bread rolls have significantly higher Na content (*P* = 0·04) than the control bread, delivering approximately 90 % of the daily Na recommendations for adult men and women (Fig. 2). The phytic acid content was very similar for the two types of breads with 0·09 and 0·1 g per two bean hull and control bread rolls, respectively.

#### Bean hull fibre fermentability

**Fig. 3.**
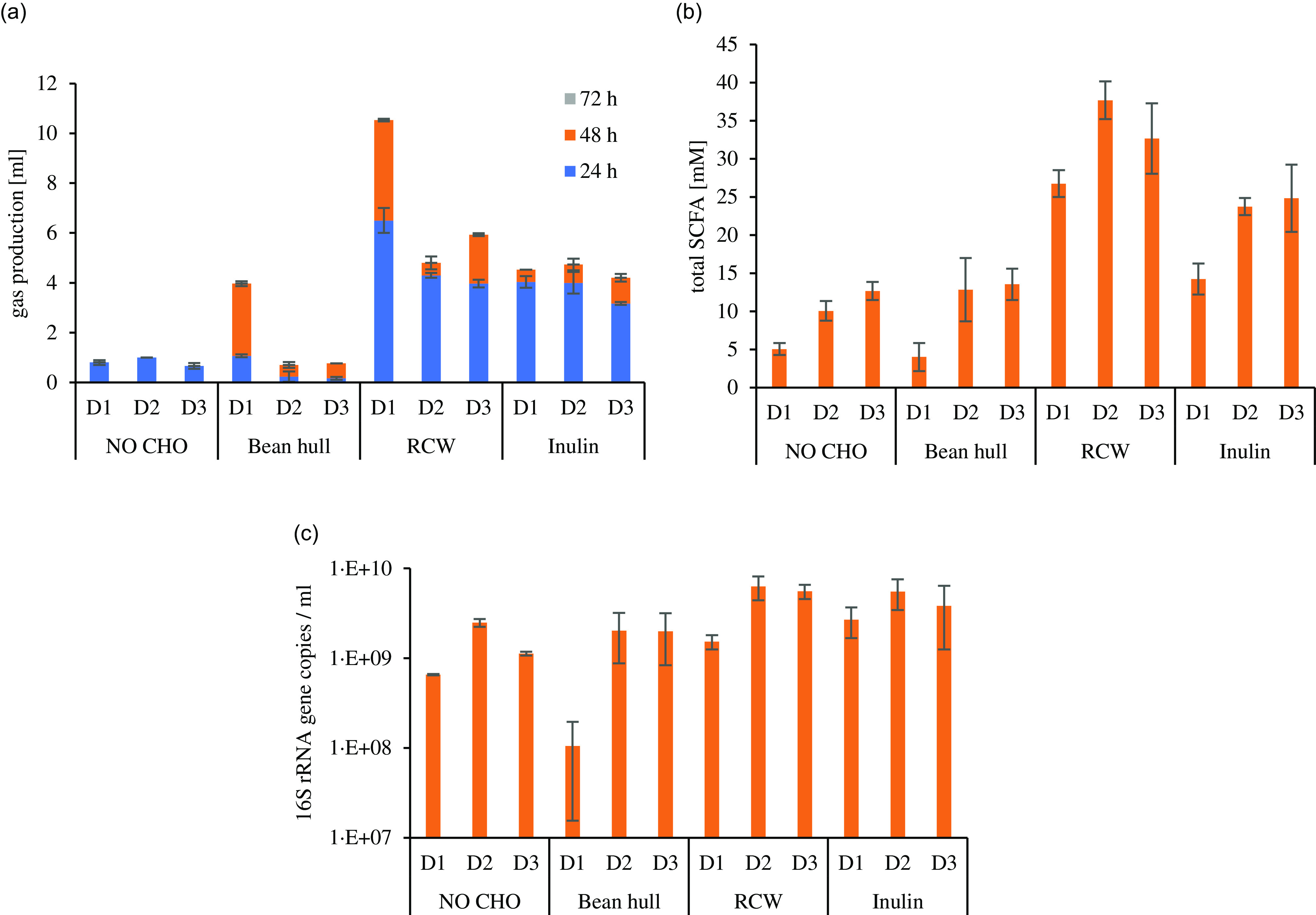
Bean hull fibre fermentability *in vitro*. The gas production over 72 h of faecal incubations with three donors (D1, D2, D3) on basal medium without added substrate (NO CHO), bean hull AIR (Bean hull), a mix of carbohydrates representing the major plant cell wall compounds (RCW) and inulin (a). The total SCFA production after 48 h of incubation (b). Total bacteria after 48 h of incubation, determined by qPCR and expressed as 16S rRNA gene copies per ml of culture (c).

Bean hull fibre has a very low fermentability *in vitro*. The gut microbial fermentability of bean hull fibre was investigated *in vitro* using human faecal batch incubations with bean hull AIR in comparison to a mix of commercially available carbohydrates representing the main components of plant cell walls (‘recombined cell wall’, RCW) and the soluble fermentable fibre inulin over 72 h with faeces from three healthy adult donors. Gas production during the incubations was very low for two of the three donors compared with the two other substrate incubations and mirrored the levels produced in the negative control (no carbohydrate present, NO CHO). Donor 1 produced more gas on bean hull AIR than in the negative control, and all donors produced gas mostly between 24 and 48 h, whereas most of the gas was produced within 24 h in the other incubations (Fig. 3). For donors 2 and 3, the trends seen based on gas production were replicated when assessing SCFA production and bacterial growth (based on total 16S rRNA gene copies per ml of culture) after 48 h of incubation, with bean hull AIR and negative control resulting in similar and lower levels compared with the positive control substrates. Donor 1 surprisingly had the lowest amount of SCFA production and total bacteria on the bean hull material, thus the microbial activity as indicated by the relatively high gas production did not lead to SCFA formation or bacterial growth (Fig. 3).

#### Bread roll plant metabolite composition

**Fig. 4.**
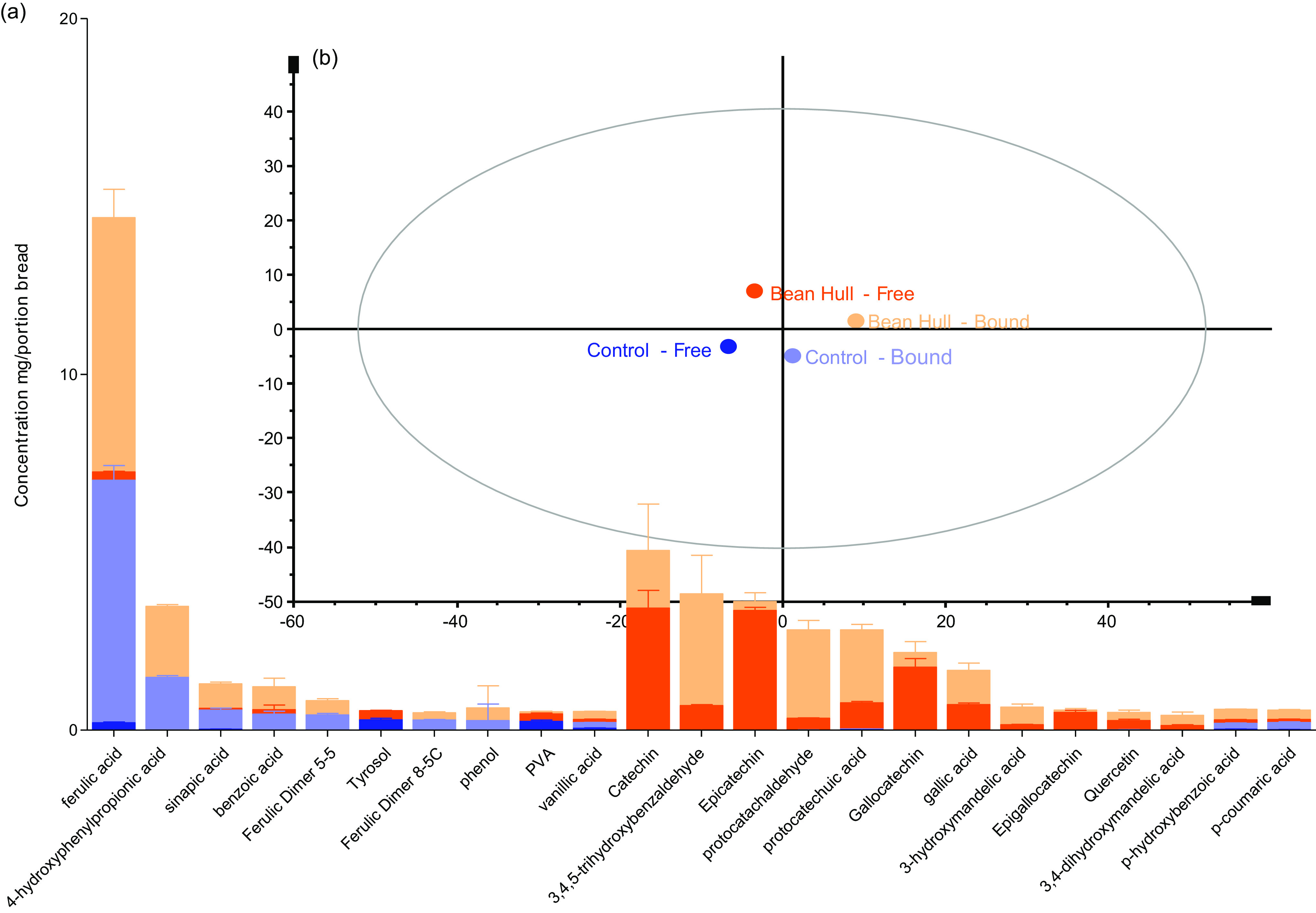
The most abundant plant metabolites measured in the bean hull and control bread rolls (mg/portion of bread rolls consumed ± sd, *n* 3) (a). The principal component analysis (PCA) of plant metabolites measured (average +/– sd, *n* 3) from bean hull and control bread rolls (b). The first two component (PC1 = 38·7 % and PC2 = 29·0 %) scores were plotted. Where the ‘free’ stands for molecules extracted without any chemical hydrolysis being molecules potentially released early in the gastrointestinal tract and the ‘bound’ molecules are those extracted following an alkaline and acid hydrolysis which are potentially released later in the gastrointestinal tract (colon).

The bean hull bread rolls are a rich source of phenolics, specifically flavonoids. The most abundant plant metabolites measured in the study bread rolls are presented in Fig. 4(a). Bean hull bread roll had a statistically higher amount of total plant metabolites measured (summing all the individual plant metabolites measured by LC-MS/MS) (30·8 mg/portion) compared with the control bread (7·9 mg/portion) (*P* = 0·04). The PCA of the plant metabolites measured showed a separation of the bean hull bread from the control bread suggesting different plant metabolite profiles between the two intervention meals (Fig. 4(b)). Ferulic acid was the most abundant phenol in both bread rolls (with 7·02 ± 0·42 mg/portion of control and 7·37 ± 0·78 mg/portion of bean hull roll), most probably being a component of the wheat flour. The separation observed on the PCA plot is associated with the statistically higher concentration of p-hydroxybenzoic acid (*P* < 0·001), epicatechin (*P* < 0·001) and catechin (*P* = 0·001) in the bean hull bread roll compared with the control. Bean hull bread roll also had statistically higher amounts of luteolin (*P* < 0·001), kaempferol (*P* < 0·001), apigenin (*P* = 0·002) and isorhamnetin (*P* < 0·001) compared with the control bread roll. The complete profile of plant metabolites measured is presented in Supplementary Table 4.

#### Plasma metabolite composition

**Fig. 5.**
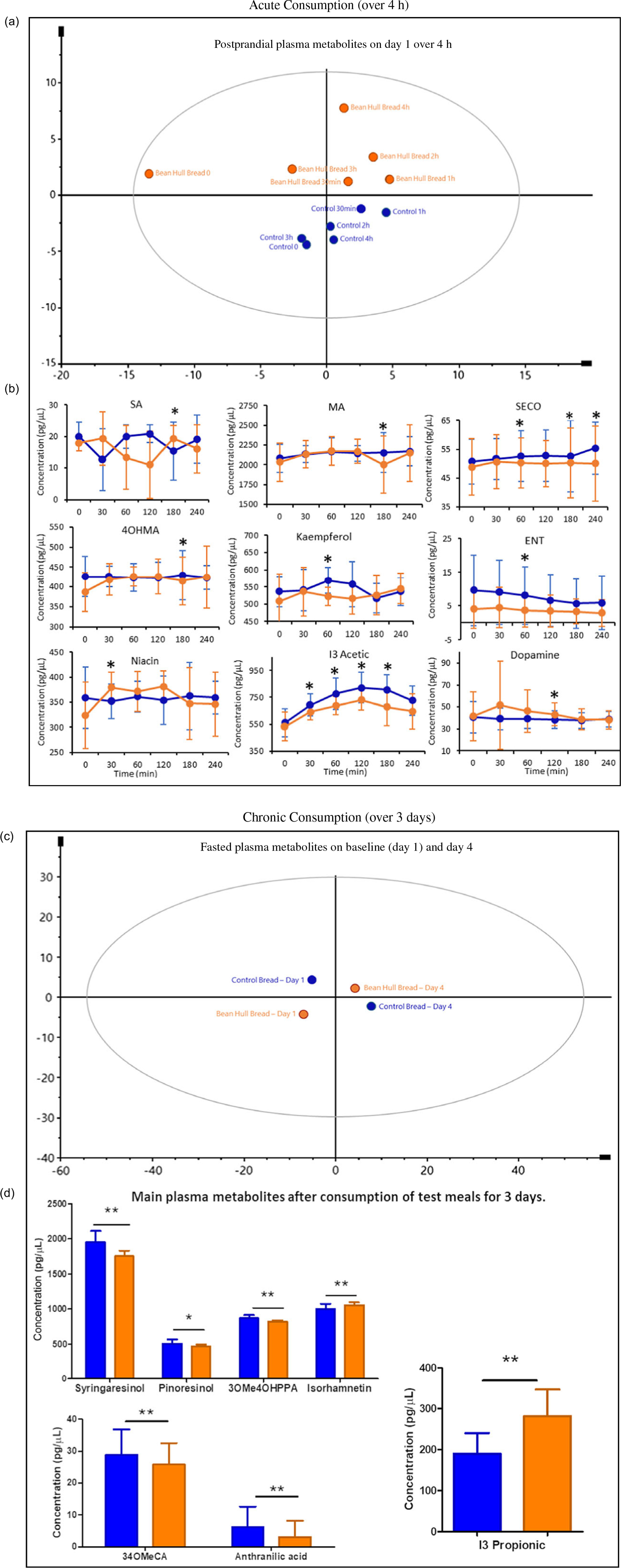
Plasma composition (over 4 h) following bean hull (orange) and control (blue) bread rolls consumption. (a) The PCA analysis of average postprandial plasma metabolites (*n* 9) at 0, 30, 60, 12, 180 and 240 min for bean hull and control bread rolls. (b) Plasma average concentration *n* 9 ± sd for the metabolites significantly postprandially following the bean hull and control breads consumption. (c) The PCA plot for average fasted metabolites initially on day 1 and after 3 days chronic consumption on day 4 of the bean hull (orange) and control bread rolls. (d) The fasted plasma average concentrations *n* 9 ± sd on day 4 for metabolites with significant differences in concentration after the consumption of bean hull *v*. control bread rolls for 3 days (b). 34OMeCA, 3,4-dimethoxycinnamic acid; 3OMe4OHPPA, 4-hydroxy-3-methoxyphenylpropionic acid; ENT, enterolactone; MA, mandelic acid; Seco, secoisolariciresinol; SA, sinapic acid; PCA, principal component analysis.

The phenolics from bean hull bread rolls have a poor systemic availability. The PCA (Fig. 5(a)) shows a separation between the postprandial metabolites (measured at 0, 30, 60, 120, 180 and 240 min) present in volunteers’ plasma following the bean hull and control bread rolls consumption on day 1. The quantitative analysis of the average metabolites present in plasma showed that nine metabolites were significantly changed over the 4 h following the consumption of the bean hull bread rolls (Fig. 5(b)). After the consumption of the control bread, indole-3-acetic acid concentration was significantly higher (*P* < 0·05) compared with the bean hull bread consumption from 30 min up to 3 h postprandially. Plasma mandelic acid and its derivative, 4-hydroxymandelic acid levels, were significantly lower at 3 h following the bean hull bread, driven mainly by one volunteer whose mandelic acid levels have dropped at 3 h. Again, only after consumption of the control bread, the secoisolariciresinol (at 1 h, 3 h, 4 h, *P* < 0·05), the sinapic acid (at 2 h, *P* = 0·014) and kaempferol (at 1h, *P* = 0·003) were significantly higher compared with the bean hull bread. Only dopamine and niacin concentrations were statistically higher at 2 h (*P* = 0·045) and at 30 min (*P* = 0·016) following the bean hull consumption compared with the control bread roll. It should be noted that certain metabolites, particularly 4-hydroxyphenylpyruvic acid, phenylpyruvic acid, phenylacetic acid, phenyllactic acid, luteolin, protocatechuic acid, mandelic acid, p-hydroxybenzoic acid, apigenin and benzoic acid were present in high concentrations throughout the 4 h on day 1 and remain unchanged following the consumption of the bean hull or the control bread rolls (online Supplementary Table 5). The highest number of plasma metabolites significantly changed at 30 and 60 min compared with baseline (0 h) was observed following the consumption of the bean hull bread (online Supplementary Table 5).

The chronic consumption of bean hull bread rolls leads to a significant increase in plasma indole-3-propionic acid, a microbial metabolite of tryptophan. The composition of fasting plasma metabolites collected following the chronic consumption of study bread rolls for 3 d (days 1 to 4 – Fig. 1) informs on the microbial metabolism of the bread rolls components. The PCA (Fig. 5(c)) showed that the overall metabolite profile in plasma differed on day 4 following the consumption of bean hull and control bread rolls for 3 days. Plasma isorhamnetin and the indole-3-propionic acid were present in significantly higher amounts (*P* = 0·014 and *P* = 0·009, respectively) after bean hull bread chronic consumption. Plasma syringaresinol (*P* = 0·001), 4-hydroxy-3-methoxyphenylpropionic acid (*P* = 0·01), 3,4-dimethoxycinnamic acid (*P* = 0·006), anthranilic acid (*P* = 0·02) and pinoresinol (*P* = 0·049) amounts were significantly higher after the control bread compared with the bean hull bread rolls consumption in the day 4 plasma (Fig. 5(d)). A larger sample size would make these differences for these compounds stronger.

It was also observed that after consuming both bread rolls for 3 days, there was a significant increase of 4-hydroxy-3-methoxyphenyl propionic acid, phenyl pyruvic acid, phenyl lactic acid in the day 4 plasma sample compared with the baseline (day 1), indicating a high microbial metabolism of tyrosine. In addition, enterolactone and secoisolariciresinol significantly increased following the consumption of bean hull and plain bread rolls on day 4 compared with the baseline (online Supplementary Table 6). A significant increase of 4-hydroxymandelic acid (*P* = 0·02), indole-3-lactic acid (*P* = 0·03) and tangeretin (*P* = 0·003) in the day 4 plasma sample compared with baseline (online Supplementary Table 6) was only observed after consumption of the bean hull bread roll. The highest number of plasma metabolites with a significant change on day 4 was following the chronic consumption of the control bread rolls (online Supplementary Table 6), suggesting that this bread was metabolised later in the gastrointestinal tract at the colon level. The amount of bile acids in plasma was variable, with no significant changes detected following the consumption of the two types of bread rolls for 3 days.

#### Plasma gastrointestinal hormones composition

The consumption of bean hull bread rolls has no effect on postprandial plasma gut hormone levels. None of the test meals have significantly affected ghrelin and GLP-1 postprandial plasma concentrations over 4 hours (AUC, *P* = 0·495 and *P* = 0·593, respectively) and (net AUC, *P* = 0·723 and *P* = 0·812, respectively). The glucagon AUC and net AUC levels were significantly lower following the bean hull bread in comparison with the control. Plasma PYY levels could not be measured in all volunteers as the concentrations were below the detection limit in many volunteers. Therefore, no statistical analysis was performed on this hormone. Following the bean hull bread roll consumption, the incremental AUC values for insulin were significantly lower (*P* = 0·04) than after the consumption of the control bread. A trend was also seen toward a reduction in insulin AUC values; however, this was NS (*P* = 0·06). *t* test analysis on each time point showed that insulin concentration was significantly lower at 90 min (*P* = 0·021) following the bean hull bread but not for any other time points in comparison to the control bread (online Supplementary Fig. 3).

#### Plasma glucose and lipids profile

The consumption of bean hull bread rolls has no effect on blood glucose and lipid levels. The blood glucose levels were not significantly different following the consumption of bean hull bread compared with the control bread rolls on day 1, for 4 hours postprandially and on day 4, on faster plasma samples following 3 days bread consumption (online Supplementary Fig. 4). Similar results were observed for plasma lipid profile on day 1 and day 4 (online Supplementary Tables 7 and 8).

#### Faecal metabolites composition

**Fig. 6.**
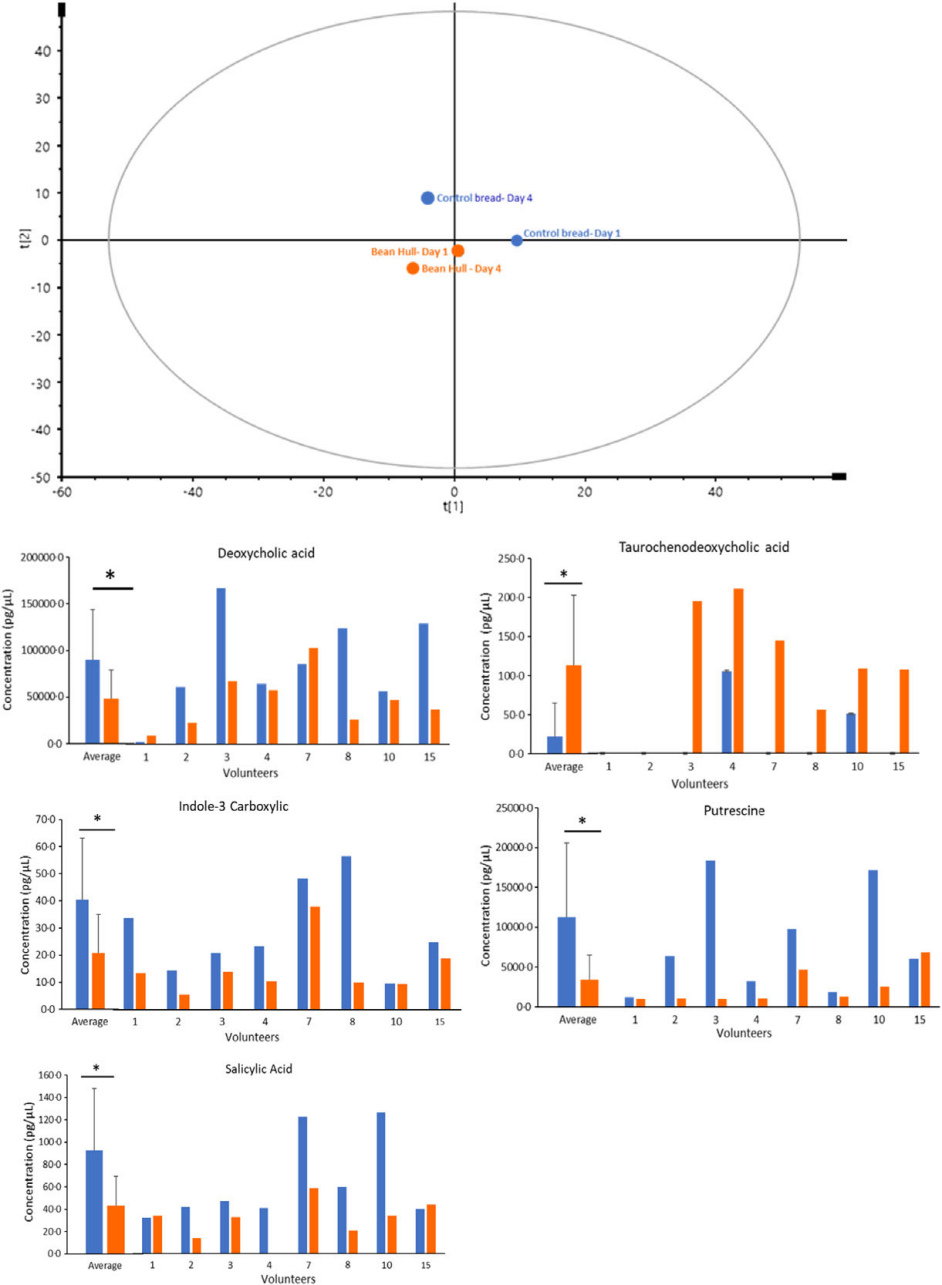
Faecal metabolites concentrations before (day 1) and after (day 4) the consumption of the study bread rolls. The PCA plot of average faecal metabolites (*n* 8) at baseline (day 1) and on day 4 following the bean hull (orange) and control (blue) bread rolls consumption. The average faecal metabolites concentration *n* 8 for faecal metabolites with significant concentration differences following the bean hull *v*. control bread rolls consumption on day 4 of the intervention (including the individual concentrations measured for each of the volunteer). * *P* < 0·05. PCA, principal component analysis.

The chronic consumption of the bean hull bread rolls significantly decreased faecal concentration of putrescine and deoxycholic acid while significantly increased taurocheno-deoxycholic acid. The PCA plot shows discrimination between the faecal metabolites produced on day 4 after 3 days of chronic consumption of the control and bean hull bread rolls (D4 control *v*. D4 bean hull). From the 194 faecal metabolites measured in faeces, putrescine (*P* = 0·035), salicylic acid (*P* = 0·028), indole-3 carboxylic acid (*P* = 0·044) and deoxycholic acid (*P* = 0·046) were significantly reduced (Fig. 6), following chronic consumption of the bean hull bread consumption compared with the control bread, while taurochenodeoxycholic acid significantly increased (*P* = 0·041). There were other differences in the concentration of microbial metabolites measured on faecal samples collected on day 4 of the study between the two interventions; however, these changes were NS. Following 3 days consumption of bean hull bread, the vanillic acid (*P* < 0·001), 1,2-hydroxybenzene (*P* = 0·01), 4-methylcatechol (*P* = 0·02), 4-hydroxy-3-methoxyphenyl acetic acid (*P* = 0·02), 2,5-dihydroxy benzoic acid (*P* = 0·03), tyramine (*P* = 0·04), cholic acid (*P* = 0·046) and chenodeoxycholic acid (*P* = 0·050) concentrations in faecal samples were significantly reduced compared with baseline levels. In contrast, indole-3-acetic acid (*P* = 0·04), taurochenodeoxycholic acid (*P* = 0·03) and 3,4,5-trimethoxy benzaldehyde (*P* = 0·04) were significantly increased (Table 3).

**Table 3.** Average concentration (*n* 8 ± sd) of the faecal metabolites on day 1 (baseline) and day 4 with a significant change following consumption of the control and bean hull bread rolls for 3 days

	Metabolites in faecal waters									
	Day 1 Control bread (baseline)		Day 4 Control bread		*P* value	Day 1 Bean hull bread (baseline)		Day 4 Bean hull bread		*P* value
	*n* 8	sd	*n* 8	sd		*n* 8	sd	*n* 8	sd	
Vanillic acid	421·88	341·00	139·90	141·95	0·049	191·00	87·10	93·56	69·64	< 0·001
1,2-Hydroxybenzene	384·10	566·60	276·90	247·90	0·37	310·56	229·50	139·78	130·62	0·01
Taurochenodeoxycholic acid	9·20	9·20	21·70	43·24	0·54	23·60	28·85	101·14	86·29	0·03
3,4,5-Trimethoxybenzaldehyde	0·00	0·00	3·10	8·90	0·35	0·00	0·00	12·96	14·75	0·04
Chenodeoxycholic acid	627·30	1172·30	286·70	810·90	0·26	1022·91	1334·30	0·00	0·00	0·05
4-Methylcatechol	1703·60	1591·90	1857·40	2006·30	0·86	1898·00	1487·50	1130·83	1178·90	0·02
2,5-Dihydroxybenzoic acid	1444·30	1842·60	472·90	563·34	0·08	789·00	761·90	258·33	269·20	0·03
Tyramine	575·20	742·70	240·20	181·26	0·28	844·06	1456·50	609·83	1354·55	0·04
Indole-3 acetic	3002·60	2458·80	1485·40	815·30	0·17	2479·50	3010·0	4903·89	4633·10	0·04
Cholic acid	4977·60	8173·00	7934·40	19 469·90	0·73	1834·10	2416·00	93·82	265·40	0·046
4-hydroxy-3-methoxyphenylacetic acid	3215·00	2880·00	1203·10	1424·20	0·11	2241·67	1010·90	1250·56	1256·00	0·02
Deoxycholic acid	47 174·40	32 155·64	89 951·60	54 157·40	0·02	53 407·60	48 474·30	43 685·00	30 336·30	0·58
Phenylacetic acid	96 106·25	49 606·40	143 812·50	75 465·70	0·02	112 088·90	42 524·60	101 822·20	59 595·60	0·56
l-methyl	442·00	612·40	1659·20	1631·60	0·02	850·10	1002·10	585·00	1151·30	0·34
Syringic acid	273·56	190·60	112·80	82·30	0·03	249·10	334·97	167·00	235·70	0·09

*P* value determined using *t* test.

#### Faecal SCFA concentrations

Consumption of bean hull bread rolls does not promote microbial SCFA production. Total SCFA concentrations in faecal samples remained mostly unchanged after interventions (Table 4). Branched-chain fatty acids isovalerate and isobutyrate concentrations tended to increase after consuming the control bread rolls compared with the bean hull (*P* = 0·07 and *P* = 0·08, respectively) (Table 4). All SCFA concentrations were decreasing, non-significantly (*P* > 0·05) following the consumption of bean hull bread rolls compared with the baseline (Table 4). Similar results were found when comparing the SCFA concentration following the two test meals on day 4 (Table 4).

**Table 4.** Faecal SCFA concentrations (mM) *n* 9 ± sd at day 1 and following the consumption of control or bean hull bread rolls day 4

	Total SCFA		Formate		Acetate		Propionate		Iso-butyrate		Butyrate		Iso-valerate		Valerate		Caproate		Succinate	
	(mM) *n* 9	sd	(mM) *n* 9	sd	(mM) *n* 9	sd	(mM) *n* 9	sd	(mM) *n* 9	sd	(mM) *n* 9	sd	(mM) *n* 9	sd	(mM) *n* 9	sd	(mM) *n* 9	sd	(mM) *n* 9	sd
Control bread day 1 (mM)	83·62	39·54	0·00	0·00	46·07	21·98	16·95	7·38	1·58	0·95	14·41	10·42	1·32	0·85	2·13	1·19	0·59	0·92	0·57	0·80
Control bread day 4 (mM)	87·31	42·81	0·16	0·47	48·39	19·52	16·57	6·89	2·33	1·54	14·50	14·22	2·09	1·55	2·42	1·57	0·61	1·16	0·24	0·48
Control bread day1 *v*. day 4 (% change)*	4·40		–		5·03		–2·24		47·36		0·62		58·68		13·51		3·54		–57·73	
Bean hull bread day 1 (mM)	85·63	40·14	0·00	0·00	50·13	24·25	16·07	5·28	1·85	0·88	13·05	10·65	1·51	0·74	2·12	0·87	0·67	0·87	0·23	0·47
Bean hull bread day 4 (mM)	67·04	11·34	0·00	0·00	39·28	7·42	14·65	3·95	1·40	0·49	8·29	3·39	1·13	0·61	1·73	0·43	0·43	0·61	0·13	0·39
Bean hull bread day1 *v*. day 4 (% change)*	–21·70		–		–21·64		–8·81		–24·33		–36·49		–25·26		–18·18		–35·51		–44·84	
Day 1 *v*. day 4 (% change)†					–18·82		–11·57		–40·05		–42·83		–46·04		–28·23		–29·67		–46·63	
ttest (comparing control *v*. bean hull on day 4)	0·18		0·35		0·24		0·40		0·08		0·15		0·07		0·16		0·44		0·63	
ttest (comparing bean hull on D1 *v*. D4)	0·18		–		0·21		0·47		0·15		0·13		0·15		0·18		0·15		0·65	

* Percentage change between baseline and post-intervention (day 1 *v*. day 4) following the bean hull and control bread rolls.

† % change between the two test meals on day 4.

#### The gut microbiota composition

**Fig. 7.**
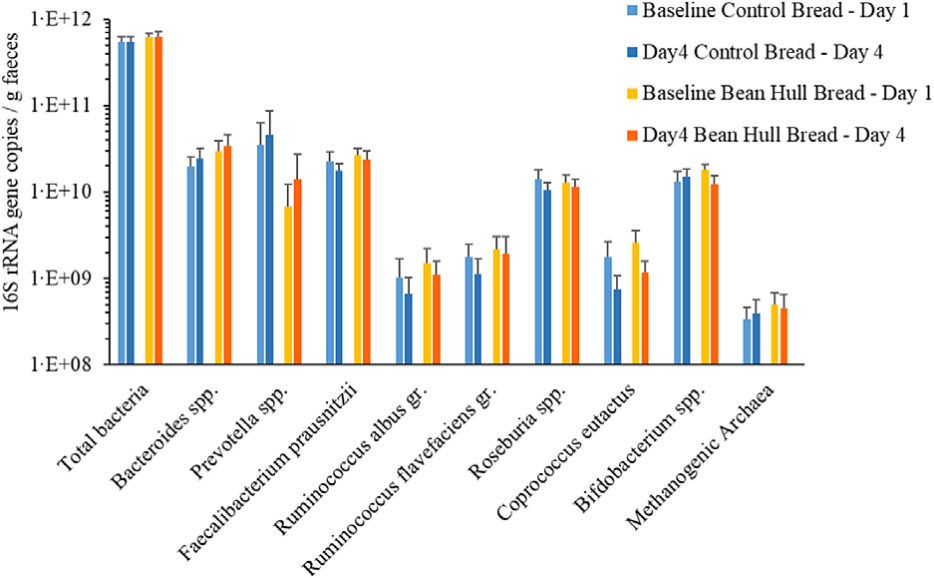
Abundance of total faecal microbiota and specific genera or species at day 1 (baseline), day 4 (chronic consumption), determined by qPCR. Average and standard error of 16S rRNA gene copies per gram faeces. ANOVA with terms for volunteer, baseline and diet was used to compare bean hull with control. qPCR, quantitative PCR.

Bean hull bread rolls consumption did not affect the abundance and composition of the gut microbes. The absolute abundance of total bacteria was determined by qPCR, but no significant differences were observed. The abundance of several genera or species within the two major phyla Bacteroidetes and Firmicutes as well as Archaea also did not appear to be influenced by the consumption of bean hull bread (Fig. 7).

#### Gastric emptying

Consumption of bean hull bread rolls had no effect on gastric emptying markers. The latency and lag phases were significantly delayed (*P* = 0·01 and *P* = 0·04, respectively) following the control bread compared with the bean hull bread roll on day 1. These two markers represent the start of emptying the meal from the stomach. There was no significant difference between meals on ascension and half-time markers (Table 5).

**Table 5.** Gastric emptying markers on day 1 following the control and bean hull bread rolls

	Control bread roll		Bean hull bread roll		*P*-value
	Mean	sd	Mean	sd	
Latency phase (min)	36·2	15·8	21·1	7·1	0·01
Ascension time (min)	112·6	29·7	125·3	31·0	0·32
Half time (min)	148·8	28·2	146·4	29·8	0·86
Lag phase (min)	96·2	22·5	77·4	16·7	0·04

#### Satiety and hunger

Consumption of bean hull bread rolls has no effect on hunger. There were no significant differences between the intervention and control bread for the visual analogue scale measures (hunger, fullness, desire, quantity) from baseline to 4 hours post the bread consumption (*P* > 0·05) (online Supplementary Fig. 6).

## Discussion

Most UK adults consume their dietary fibre from wheat-based cereals^(31,32)^. To understand how the consumption of the dietary fibre can be diversified and at the same time increased, the present study assessed the suitability of new sources of fibre such as bean hulls to complement, contribute and boost the fibre intake of the UK population. This study showed that two bean hull bread rolls provided over 85 % of the recommended DF, which was more than three times higher than the DF provided by the control bread roll (wheat bread). Dietary fibre has a vital role in promoting overall metabolic health. It has also been related to preventing and treating other pathologies, including CVD, colonic health and gut motility^(33)^. Current recommendations for dietary fibre intake for adults in the UK are 30 g per day^(1)^. However, most adults in the UK are only eating an average of approximately 18 g of dietary fibre per day. Therefore, two bean hull bread rolls could easily contribute to meeting the recommended daily dietary fibre consumption. One of the richest sources of dietary fibre are wheat bran-based products. The wheat bran NSP comprise 47·4 % xylose, 40·9 % arabinose and 8·4 % glucose^(25)^. Bean hull bread appeared to have a similar monomeric composition but in different ratio (19·2 % xylose, 5·35 % arabinose and 54·6 % glucose).

Overall, bean hull bread had a significantly higher mineral content than the control bread roll. Thus, regular consumption of bean hull bread roll could further contribute towards meeting the recommended daily intake compared with the control bread, and it can be an alternative cost-effective approach to control some mineral deficiencies. In addition, bean hull bread had low phytic acid content, suggesting that microelements from bean hull bread potentially are bioavailable^(34)^.

The most abundant phenolic compounds found in the bean hull bread roll were ferulic acid, the hydrogenated product of the 5–5 linked dimer and the 8–5 linked ferulic dimer. These are components of the wheat from the bean hull bread, as wheat flour is rich in 5–5 linked dimer ferulic acid. Catechin, epicatechin, gallocatechin, epigallocatechin and quercetin were also found in higher concentrations in the bean hull bread roll; however, they were found in much lower quantities than in well-known sources such as green tea (10 to 20 times higher in catechins than bean hull bread roll)^(35)^ or apples where quercetin concentration ranges from 99·6 ± 5·4 to 495·3 ± 44·0 mg in 100 g^(36)^. There is abundant research reporting the beneficial health benefits of flavonoids on the pathogenesis of diabetes^(37)^ lipid profile^(38)^ through their anti-microbial and antioxidant and anti-inflammatory actions^(39)^. However, even though the present study showed that the concentration of these flavonoids was higher in the free than bound form in the bean hull bread roll, the consumption of this bread roll did not increase these phenols in the plasma over 4 h following the consumption. This suggests that the phenolics identified from the bean hull bread were poorly bioavailable systemically. This calls for further formulation of bean hull matrix to improve the bioavailability of these key bioactives.

The present study showed that chronic consumption of the bean hull bread rolls significantly increases plasma indole-3-propionic acid (IPA). IPA is a microbial product from dietary tryptophan metabolite absorbed from the gut into the bloodstream^(40,41)^. Higher serum IPA has been found to be directly associated with higher dietary fibre intake, which potentially could explain the study results as one bean hull bread roll provides 12·84 g fibre (as soluble and insoluble NSP)^(40,41)^. Recently it was suggested that high levels of IPA have a protective role against the development of type 2 diabetes (T2D)^(41)^. The effect of IPA on lowering T2D might be mediated by direct effect of IPA on β-cell function. Moreover, higher IPA could ameliorate inflammation and cell oxidative damage which consequently can lower T2D^(40)^. Thus, foods that increase plasma IPA are potential candidates to promote the prevention of T2D.

The isorhamnetin was also present in significantly higher quantities in plasma on day 4 following bean hull bread roll consumption. Isorhamnetin is a methylated flavanol and has positive impacts on many health functions, including risks of hyperglycaemia and CVD^(42)^.

Three days consumption of bean hull bread rolls also led to a decrease of polyamine putrescine, in volunteers’ faecal samples compared with the control bread. High levels of polyamines are toxic and associated with various chronic diseases, including cancer^(43)^. A review by Linsalata et al. (2014)^(44)^ reported that high intake of phytophenols can have chemopreventive properties by affecting the polyamine metabolic pathways. Therefore, the consumption of bean hull-rich foods could be considered as part of nutritional strategies to prevent chronic disease development. Similarly, the secondary bile acid, deoxycholic acid formed by microbial conversion of the primary acids formed in the liver was significantly reduced following the chronic consumption of the bean hull bread. It has been reported that deoxycholic acid induce tumorigenesis in the bowel due to the generation of reactive oxygen species which cause DNA damage through the promotion of mucosal and DNA damage^(45)^.

The dietary fibre can affect health through multiple mechanisms, including its fermentation in the large intestine to yield SCFA^(25)^. After the consumption of the bean hull bread roll for 3 days, the SCFA concentration was reduced compared with the control bread consumption. Potentially with a higher number of participants, this decrease would have reached significance. No differences in faecal microbiota abundance or composition were detected here over time. These findings were also supported by the *in vitro* fermentability experiments on bean hull, leading to similar results. These results are also in agreement with a study made by Karatas, Günay and Sayar (2017)^(46)^ reporting a lower *in vitro* fermentability of the bean hull compared with its seed. All these outcomes collectively suggest that the bean hull fibre is poorly fermented by the human gut microbiota following the consumption of the fortified bread.

### Study limitations

The present study has several limitations. A relatively small sample size of nine participants limits the conclusions that can be drawn from the study; however, this sample size does not weaken the significant differences which have been found.

### Conclusion

The habitual consumption of the bean hull bread rolls could potentially provide some benefits against T2D due to the increased production of indole-3-propionic acid, and against cancer, by reducing the production of putrescine and deoxycholic acid, which can cause DNA damage. Bean hull fortified bread rolls could be utilised as a source of fibre in the diet and contribute to the daily micronutrient requirements. However, further food formulation work is necessary to improve the bean hulls’ phytochemicals systemic bioavailability and increase its fibre microbial fermentation in healthy adults.

## Supplementary material

For supplementary material accompanying this paper visit https://doi.org/10.1017/S0007114523000491. click here to view supplementary material
