# Supplementary material for: Habitual consumption of high-fibre bread fortified with bean hulls increased plasma indole-3-propionic concentration and decreased putrescine and deoxycholic acid faecal concentrations in healthy volunteers
Source: Br J Nutr. 2023 Feb 27;130(9):1521–36. doi: 10.1017/S0007114523000491 (PMC10551484; doi:10.1017/S0007114523000491)
Supplement: Supplementary file 1 [file S0007114523000491sup001.docx]

**Habitual consumption of high-fibre bread fortified with bean hulls increased plasma indole-3-propionic concentration and decreased putrescine and deoxycholic acid faecal concentrations in healthy volunteers**

Marietta Sayegh, Qian Qian Ni, Viren Ranawana, Vassilis Raikos, Nick J. Hayward, Helen Hayes, Gary Duncan, Louise Cantlay, Freda Farquharson, Michael Solvang, Graham Horgan, Petra Louis, Wendy Russell, Miriam Clegg, Frank Thies and Madalina Neacsu

**Supplementary Table 1.** Nutritional composition of the pre-test

|  | **Weight (g)** | **Fat (g)** | **Energy (kcal)** | **Protein (g)** | **CHO (g)** | **Fibre (g)** | **Salt (g)** |
| --- | --- | --- | --- | --- | --- | --- | --- |
| Vegetarian Paella | 378.7 | 7.5 | 374.9 | 9.1 | 68.2 | 5.6 | 1.9 |
| Semi-skilled milk | 137.0 | 2.3 | 64.8 | 4.8 | 6.4 | - | 0.1 |
| Muller light toffee yoghurt | 175.0 | 0.2 | 83.6 | 7.2 | 13.8 | - | 0.4 |
| Digestive biscuit, plain | 16.0 | 3.6 | 76.7 | 1.1 | 10.3 | 0.6 | 0.2 |
| **TOTAL** | **706.7** | **13.6** | **600.0** | **22.1** | **98.8** | **6.2** | **2.6** |

CHO: carbohydrates

**Supplementary Table 2**. ^1,2^Nutritional information of the meals provided to the volunteers for Days 1, 2, 3.

| **Day** | **Meal** | **Food** | **Weight (g)** | **Energy (kcal)** | **Fat (g)** | **Protein (g)** | **CHO (g)** | **Fibre (g)** | **Salt (g)** |  |
| --- | --- | --- | --- | --- | --- | --- | --- | --- | --- | --- |
| **Vegetarian Intervention Meals (2000 kcal)** | | | | | | | | | | |
| 1 | Lunch | Tesco Mushroom risotto | 366.0 | 331.1 | 8.4 | 12.4 | 51.2 | 5.1 | 1.5 |  |
| 1 | Lunch | Mars bar | 43.2 | 186.2 | 7.2 | 1.9 | 29.9 | 0.0 | 0.2 |  |
| 1 | Dinner | BEAN HULL BREAD ROLL | 152.0 | 352.0 | 5.3 | 12.9 | 51.7 | 22.0 | 1.8 |  |
| 1 | Dinner | Tesco Sunflower spread | 10.1 | 49.6 | 5.6 | 0.0 | 0.1 | 0.0 | 0.1 |  |
| 1 | Dinner | Tesco Macaroni and cheese pasta pot | 180.0 | 276.9 | 7.7 | 10.1 | 39.8 | 2.9 | 0.9 |  |
| 1 | Dinner | Cheese, Cheddar-type, reduced fat | 58.9 | 183.9 | 13.0 | 16.4 | 0.5 | 0.0 | 1.1 |  |
| 1 | Dinner | Alpro Original fresh | 200.0 | 77.3 | 3.6 | 6.0 | 5.0 | 1.0 | 0.2 |  |
| 1 | Dinner | Double cream | 18.8 | 91.8 | 10.1 | 0.3 | 0.3 | 0.0 | 0.0 |  |
| 1 | Dinner | Digestive biscuit, plain | 6.6 | 31.6 | 1.5 | 0.4 | 4.3 | 0.3 | 0.1 |  |
| 1 | Dinner | Tesco peaches in light syrup | 77.8 | 35.6 | 0.0 | 0.2 | 8.7 | 0.7 | 0.0 |  |
| **TOTAL (Day 1)** | | | **1290.4** | **2000.0** | **67.8** | **73.8** | **260.4** | **53.9** | **7.6** |  |
| 2 | Breakfast | BEAN HULL BREAD ROLL | 152.0 | 352.0 | 5.3 | 12.9 | 51.7 | 22.0 | 1.8 |  |
| 2 | Breakfast | Rice Krispies | 38.0 | 145.3 | 0.4 | 2.2 | 34.7 | 0.3 | 0.4 |  |
| 2 | Breakfast | Jam | 8.8 | 23.5 | 0.0 | 0.1 | 6.1 | 0.0 | 0.0 |  |
| 2 | Breakfast | Tesco Sunflower spread | 9.5 | 46.6 | 5.2 | 0.0 | 0.1 | 0.0 | 0.1 |  |
| 2 | Breakfast | Orange juice, unsweetened | 99.9 | 36.5 | 0.0 | 0.9 | 8.6 | 0.0 | 0.0 |  |
| 2 | Breakfast | Alpro Original fresh | 350.0 | 135.3 | 6.3 | 10.5 | 8.8 | 1.8 | 0.3 |  |
| 2 | Lunch | BEAN HULL BREAD ROLL | 152.0 | 352.0 | 5.3 | 12.9 | 51.7 | 22.0 | 1.8 |  |
| 2 | Lunch | Tesco Sunflower spread | 9.0 | 44.2 | 5.0 | 0.0 | 0.1 | 0.0 | 0.1 |  |
| 2 | Lunch | Cheese, Cheddar-type, reduced fat | 61.9 | 193.2 | 13.7 | 17.3 | 0.5 | 0.0 | 1.1 |  |
| 2 | Lunch | Coleslaw Tesco | 37.0 | 62.6 | 5.8 | 0.4 | 2.2 | 0.7 | 0.2 |  |
| 2 | Lunch | walkers ready salted crisps | 25.0 | 133.0 | 8.0 | 1.5 | 14.2 | 1.1 | 0.4 |  |
| 2 | Dinner | Chocolate, milk | 20.0 | 104.7 | 6.2 | 1.5 | 11.2 | 0.5 | 0.0 |  |
| 2 | Dinner | Quorn Tantalising tikka masala | 375.0 | 396.0 | 6.8 | 13.5 | 67.9 | 11.3 | 1.8 |  |
| **TOTAL (Day 2)** | | | **1338.1** | **2000.0** | **68.0** | **73.5** | **257.5** | **59.4** | **7.9** |  |
| 3 | Breakfast | BEAN HULL BREAD ROLL | 152.0 | 352.0 | 5.3 | 12.9 | 51.7 | 22.0 | 1.8 |  |
| 3 | Breakfast | Special K | 29.5 | 114.5 | 0.4 | 2.7 | 25.4 | 1.3 | 0.3 |  |
| 3 | Breakfast | Tesco Sunflower spread | 9.4 | 46.1 | 5.2 | 0.0 | 0.1 | 0.0 | 0.1 |  |
| 3 | Breakfast | jam | 16.0 | 42.6 | 0.0 | 0.1 | 11.0 | 0.0 | 0.0 |  |
| 3 | Breakfast | Orange juice, unsweetened | 198.8 | 72.7 | 0.0 | 1.8 | 17.1 | 0.0 | 0.0 |  |
| 3 | Breakfast | Alpro Original fresh | 200.0 | 77.3 | 3.6 | 6.0 | 5.0 | 1.0 | 0.2 |  |
| 3 | Lunch | BEAN HULL BREAD ROLL | 152.0 | 352.0 | 5.3 | 12.9 | 51.7 | 22.0 | 1.8 |  |
| 3 | Lunch | Cucumber, raw | 24.0 | 3.7 | 0.1 | 0.2 | 0.3 | 0.2 | 0.0 |  |
| 3 | Lunch | Mayonnaise reduced fat | 15.2 | 43.2 | 4.3 | 0.2 | 1.2 | 0.0 | 0.0 |  |
| 3 | Lunch | Boiled egg | 121.0 | 172.2 | 11.6 | 17.1 | 0.0 | 0.0 | 0.5 |  |
| 3 | Lunch | walkers ready salted crisps | 25.0 | 133.0 | 8.0 | 1.5 | 14.2 | 1.1 | 0.4 |  |
| 3 | Dinner | Quorn cottage pie | 380.0 | 305.0 | 8.0 | 12.9 | 41.8 | 11.4 | 1.1 |  |
| 3 | Dinner | Frozen peas cooked | 48.0 | 39.9 | 0.4 | 2.7 | 5.2 | 2.7 | 0.0 |  |
| 3 | Dinner | Tesco individual strawberry cheesecake | 100.0 | 270.7 | 15.5 | 2.9 | 31.5 | 0.6 | 0.2 |  |
| **TOTAL (Day 3)** | | | **1470.9** | **2000.0** | **67.8** | **73.9** | **256.1** | **62.2** | **6.3** |  |
| **Day** | **Meal** | **Food** | **Weight (g)** | **Energy (kcal)** | **Fat (g)** | **Protein (g)** | **CHO (g)** | **Fibre (g)** | **Salt (g)** |  |
| **Non-vegetarian Intervention Meals (2000 kcal)** | | | | | | | | | | |
| 1 | Lunch | Tesco Mushroom risotto | 325.00 | 293.98 | 7.48 | 11.05 | 45.50 | 4.55 | 1.30 |  |
| 1 | Lunch | Tesco ready to eat flame grilled chicken mini fillet | 64.20 | 69.51 | 0.71 | 14.83 | 0.77 | 0.00 | 0.32 |  |
| 1 | Lunch | Mars bar | 51.00 | 219.80 | 8.52 | 2.24 | 35.34 | 0.00 | 0.21 |  |
| 1 | Dinner | BEAN HULL BREAD ROLL | 152.00 | 352.00 | 5.34 | 12.89 | 51.68 | 21.96 | 1.79 |  |
| 1 | Dinner | Tesco Sunflower spread | 17.80 | 87.34 | 9.79 | 0.02 | 0.16 | 0.00 | 0.23 |  |
| 1 | Dinner | Tesco Macaroni and cheese pasta pot | 203.00 | 312.27 | 8.73 | 11.37 | 44.86 | 3.25 | 1.02 |  |
| 1 | Dinner | Cheese, Cheddar-type, reduced fat | 12.00 | 37.46 | 2.65 | 3.35 | 0.10 | 0.00 | 0.22 |  |
| 1 | Dinner | Semi-skimmed milk, pasteurised | 121.80 | 57.58 | 2.07 | 4.26 | 5.72 | 0.00 | 0.13 |  |
| 1 | Dinner | double cream | 32.00 | 156.27 | 17.18 | 0.51 | 0.54 | 0.00 | 0.02 |  |
| 1 | Dinner | Tesco peaches in light syrup | 67.60 | 30.97 | 0.00 | 0.20 | 7.57 | 0.61 | 0.00 |  |
| **TOTAL (Day 1)** | | | **1223.40** | **2000.00** | **67.79** | **73.77** | **261.18** | **52.33** | **7.04** |  |
| 2 | Breakfast | BEAN HULL BREAD ROLL | 152.00 | 352.00 | 5.34 | 12.89 | 51.68 | 21.96 | 1.79 |  |
| 2 | Breakfast | Rice Krispies | 25.00 | 95.62 | 0.25 | 1.43 | 22.80 | 0.18 | 0.23 |  |
| 2 | Breakfast | Jam | 19.00 | 50.65 | 0.00 | 0.11 | 13.11 | 0.00 | 0.01 |  |
| 2 | Breakfast | Tesco Sunflower spread | 10.00 | 49.07 | 5.50 | 0.01 | 0.09 | 0.00 | 0.13 |  |
| 2 | Breakfast | Orange juice, unsweetened | 100.00 | 36.58 | 0.00 | 0.90 | 8.60 | 0.00 | 0.00 |  |
| 2 | Breakfast | Semi-skimmed milk, pasteurised | 129.30 | 61.12 | 2.20 | 4.53 | 6.08 | 0.00 | 0.14 |  |
| 2 | Lunch | BEAN HULL BREAD ROLL | 152.00 | 352.00 | 5.34 | 12.89 | 51.68 | 21.96 | 1.79 |  |
| 2 | Lunch | Heinz potato and leek soup | 160.00 | 76.02 | 2.88 | 1.28 | 11.36 | 0.96 | 0.96 |  |
| 2 | Lunch | Tesco Sunflower spread | 9.60 | 47.11 | 5.28 | 0.01 | 0.09 | 0.00 | 0.12 |  |
| 2 | Lunch | Cheese, Cheddar-type, reduced fat | 17.00 | 53.07 | 3.76 | 4.74 | 0.14 | 0.00 | 0.31 |  |
| 2 | Lunch | Tesco Wafer Thin Honey Roast Ham | 11.00 | 13.12 | 0.29 | 2.38 | 0.21 | 0.07 | 0.22 |  |
| 2 | Lunch | walkers ready salted crisps | 25.00 | 133.01 | 7.98 | 1.53 | 14.15 | 1.08 | 0.35 |  |
| 2 | Dinner | chocolate, milk | 20.00 | 104.75 | 6.22 | 1.46 | 11.20 | 0.46 | 0.04 |  |
| 2 | Dinner | Tesco chicken curry with rice | 414.00 | 618.03 | 22.77 | 29.81 | 69.97 | 5.38 | 1.45 |  |
| **TOTAL (Day 2)** | | | **1243.90** | **2000.00** | **67.79** | **73.96** | **261.14** | **52.05** | **7.54** |  |
| 3 | Breakfast | BEAN HULL BREAD ROLL | 152.00 | 352.00 | 5.34 | 12.89 | 51.68 | 21.96 | 1.79 |  |
| 3 | Breakfast | Special K | 30.00 | 116.42 | 0.45 | 2.70 | 25.83 | 1.35 | 0.30 |  |
| 3 | Breakfast | Tesco Sunflower spread | 12.10 | 59.37 | 6.66 | 0.01 | 0.11 | 0.00 | 0.16 |  |
| 3 | Breakfast | jam | 20.70 | 55.18 | 0.00 | 0.12 | 14.28 | 0.00 | 0.02 |  |
| 3 | Breakfast | Orange juice, unsweetened | 157.70 | 57.69 | 0.00 | 1.42 | 13.56 | 0.00 | 0.00 |  |
| 3 | Breakfast | Semi-skimmed milk, pasteurised | 101.00 | 47.75 | 1.72 | 3.54 | 4.75 | 0.00 | 0.11 |  |
| 3 | Lunch | BEAN HULL BREAD ROLL | 152.00 | 352.00 | 5.34 | 12.89 | 51.68 | 21.96 | 1.79 |  |
| 3 | Lunch | Cucumber, raw | 24.00 | 3.67 | 0.14 | 0.24 | 0.29 | 0.17 | 0.00 |  |
| 3 | Lunch | Mayonnaise reduced fat | 30.00 | 85.26 | 8.43 | 0.30 | 2.46 | 0.00 | 0.00 |  |
| 3 | Lunch | tuna in brine | 28.50 | 31.38 | 0.29 | 7.10 | 0.00 | 0.00 | 0.21 |  |
| 3 | Lunch | walkers ready salted crisps | 25.00 | 133.01 | 7.98 | 1.53 | 14.15 | 1.08 | 0.35 |  |
| 3 | Dinner | Tesco finest cottage pie | 390.00 | 434.78 | 15.60 | 26.52 | 47.58 | 3.51 | 3.12 |  |
| 3 | Dinner | Frozen peas cooked | 31.00 | 25.79 | 0.28 | 1.77 | 3.35 | 1.74 | 0.00 |  |
| 3 | Dinner | Tesco individual strawberry cheesecake | 100.00 | 270.72 | 15.50 | 2.90 | 31.50 | 0.60 | 0.17 |  |
| **TOTAL (Day 3)** | | | **1254.00** | **2000.03** | **67.71** | **73.92** | **261.22** | **52.37** | **8.01** |  |
| **Day** | **Meal** | **Food** | **Weight (g)** | **Energy (kcal)** | **Fat (g)** | **Protein (g)** | **CHO (g)** | **Fibre (g)** | **Salt (g)** |  |
| **Vegetarian Intervention Meals (2500 kcal)** | | | | | | | | | | |
| 1 | Lunch | Tesco Mushroom risotto | 366 | 331.06 | 8.42 | 12.44 | 51.24 | 5.12 | 1.46 |  |
| 1 | Lunch | Mars bar | 43.2 | 186.18 | 7.21 | 1.90 | 29.94 | 0.00 | 0.18 |  |
| 1 | Dinner | BEAN HULL BREAD ROLL | 152 | 352.00 | 5.34 | 12.89 | 51.68 | 21.96 | 1.79 |  |
| 1 | Dinner | Tesco Sunflower spread | 11.6 | 56.92 | 6.38 | 0.01 | 0.10 | 0.00 | 0.15 |  |
| 1 | Dinner | Tesco Macaroni and cheese pasta pot | 314 | 483.02 | 13.50 | 17.58 | 69.39 | 5.02 | 1.57 |  |
| 1 | Dinner | Cheese, Cheddar-type, reduced fat | 62.6 | 195.41 | 13.83 | 17.47 | 0.50 | 0.00 | 1.13 |  |
| 1 | Dinner | Semi-skimmed milk, pasteurised | 398.9 | 188.57 | 6.78 | 13.96 | 18.75 | 0.00 | 0.43 |  |
| 1 | Dinner | Double cream | 20.5 | 100.11 | 11.01 | 0.33 | 0.35 | 0.00 | 0.01 |  |
| 1 | Dinner | Digestive biscuit, plain | 31 | 148.55 | 6.91 | 2.08 | 20.03 | 1.18 | 0.31 |  |
| 1 | Dinner | Tesco peaches in light syrup | 177.4 | 81.27 | 0.00 | 0.53 | 19.87 | 1.60 | 0.00 |  |
| **TOTAL (Day 1)** | | | **1754.2** | **2500.0** | **84.72** | **92.23** | **330.78** | **56.85** | **8.83** |  |
| 2 | Breakfast | BEAN HULL BREAD ROLL | 152.0 | 352.0 | 5.3 | 12.9 | 51.7 | 22.0 | 1.8 |  |
| 2 | Breakfast | Rice Krispies | 38.0 | 145.3 | 0.4 | 2.2 | 34.7 | 0.3 | 0.4 |  |
| 2 | Breakfast | jam | 16.8 | 44.8 | 0.0 | 0.1 | 11.6 | 0.0 | 0.0 |  |
| 2 | Breakfast | Tesco Sunflower spread | 14.4 | 70.7 | 7.9 | 0.0 | 0.1 | 0.0 | 0.2 |  |
| 2 | Breakfast | Orange juice, unsweetened | 260.0 | 95.1 | 0.0 | 2.3 | 22.4 | 0.0 | 0.0 |  |
| 2 | Breakfast | Semi-skimmed milk, pasteurised | 450.0 | 212.7 | 7.7 | 15.8 | 21.2 | 0.0 | 0.5 |  |
| 2 | Lunch | BEAN HULL BREAD ROLL | 152.0 | 352.0 | 5.3 | 12.9 | 51.7 | 22.0 | 1.8 |  |
| 2 | Lunch | Tesco Sunflower spread | 9.6 | 47.1 | 5.3 | 0.0 | 0.1 | 0.0 | 0.1 |  |
| 2 | Lunch | Cheese, Cheddar-type, reduced fat | 80.0 | 249.7 | 17.7 | 22.3 | 0.6 | 0.0 | 1.4 |  |
| 2 | Lunch | Coleslaw Tesco | 50.0 | 84.6 | 7.9 | 0.5 | 3.0 | 1.0 | 0.3 |  |
| 2 | Lunch | walkers ready salted crisps | 25.0 | 133.0 | 8.0 | 1.5 | 14.2 | 1.1 | 0.4 |  |
| 2 | Dinner | chocolate, milk | 30.0 | 157.1 | 9.3 | 2.2 | 16.8 | 0.7 | 0.1 |  |
| 2 | Dinner | Quorn Tantalising tikka masala | 550.0 | 580.8 | 9.9 | 19.8 | 99.6 | 16.5 | 2.6 |  |
| **TOTAL (Day 2)** | | | **1827.8** | **2500.0** | **84.6** | **92.5** | **327.4** | **63.4** | **9.5** |  |
| 3 | Breakfast | BEAN HULL BREAD ROLL | 152.0 | 352.0 | 5.3 | 12.9 | 51.7 | 22.0 | 1.8 |  |
| 3 | Breakfast | Special K | 51.0 | 197.9 | 0.8 | 4.6 | 43.9 | 2.3 | 0.5 |  |
| 3 | Breakfast | Tesco Sunflower spread | 23.5 | 115.3 | 12.9 | 0.0 | 0.2 | 0.0 | 0.3 |  |
| 3 | Breakfast | Jam | 26.0 | 69.3 | 0.0 | 0.2 | 17.9 | 0.0 | 0.0 |  |
| 3 | Breakfast | Orange juice, unsweetened | 291.0 | 106.4 | 0.0 | 2.6 | 25.0 | 0.0 | 0.0 |  |
| 3 | Breakfast | Semi-skimmed milk, pasteurised | 290.2 | 137.2 | 4.9 | 10.2 | 13.6 | 0.0 | 0.3 |  |
| 3 | Lunch | BEAN HULL BREAD ROLL | 152.0 | 352.0 | 5.3 | 12.9 | 51.7 | 22.0 | 1.8 |  |
| 3 | Lunch | Cucumber, raw | 30.0 | 4.6 | 0.2 | 0.3 | 0.4 | 0.2 | 0.0 |  |
| 3 | Lunch | Mayonnaise reduced fat | 40.0 | 113.7 | 11.2 | 0.4 | 3.3 | 0.0 | 0.0 |  |
| 3 | Lunch | Boiled egg | 154.0 | 219.2 | 14.8 | 21.7 | 0.0 | 0.0 | 0.6 |  |
| 3 | Lunch | walkers ready salted crisps | 25.0 | 133.0 | 8.0 | 1.5 | 14.2 | 1.1 | 0.4 |  |
| 3 | Dinner | Quorn cottage pie | 540.0 | 427.9 | 5.4 | 20.5 | 71.3 | 12.4 | 3.8 |  |
| 3 | Dinner | Frozen peas cooked | 31.0 | 25.8 | 0.3 | 1.8 | 3.3 | 1.7 | 0.0 |  |
| 3 | Dinner | Tesco individual strawberry cheesecake | 100.0 | 270.7 | 15.5 | 2.9 | 31.5 | 0.6 | 0.2 |  |
| **TOTAL (DAY 3)** | | | **1905.7** | **2500.0** | **84.7** | **92.5** | **328.0** | **62.3** | **9.6** |  |
| **Day** | **Meal** | **Food** | **Weight (g)** | **Energy (kcal)** | **Fat (g)** | **Protein (g)** | **CHO (g)** | **Fibre (g)** | **Salt (g)** |  |
| **Non-vegetarian Intervention Meals (2500 kcal)** | | | | | | | | | | |
| 1 | Lunch | Tesco chicken and asparagus Risotto | 358.0 | 334.9 | 5.0 | 25.8 | 45.8 | 5.4 | 1.1 |  |
| 1 | Lunch | Mars bar | 51.0 | 219.8 | 8.5 | 2.2 | 35.3 | 0.0 | 0.2 |  |
| 1 | Dinner | BEAN HULL BREAD ROLL | 152.0 | 352.0 | 5.3 | 12.9 | 51.7 | 22.0 | 1.8 |  |
| 1 | Dinner | Tesco Sunflower spread | 12.5 | 61.3 | 6.9 | 0.0 | 0.1 | 0.0 | 0.2 |  |
| 1 | Dinner | Tesco Macaroni and cheese pasta pot | 314.0 | 483.0 | 13.5 | 17.6 | 69.4 | 5.0 | 1.6 |  |
| 1 | Dinner | Cheese, Cheddar-type, reduced fat | 33.0 | 103.0 | 7.3 | 9.2 | 0.3 | 0.0 | 0.6 |  |
| 1 | Dinner | Semi-skimmed milk, pasteurised | 236.0 | 111.6 | 4.0 | 8.3 | 11.1 | 0.0 | 0.3 |  |
| 1 | Dinner | double cream | 40.2 | 196.3 | 21.6 | 0.6 | 0.7 | 0.0 | 0.0 |  |
| 1 | Dinner | Hartleys ready to eat jelly strawberry | 125.0 | 64.8 | 0.1 | 0.1 | 16.5 | 0.0 | 0.1 |  |
| 1 | Dinner | Digestive biscuit, plain | 32.0 | 153.3 | 7.1 | 2.1 | 20.7 | 1.2 | 0.3 |  |
| 1 | Dinner | Tesco peaches in light syrup | 94.0 | 43.1 | 0.0 | 0.3 | 10.5 | 0.8 | 0.0 |  |
| **TOTAL (Day 1)** | | | **1624.7** | **2500.0** | **84.7** | **92.2** | **331.0** | **56.4** | **7.9** |  |
| 2 | Breakfast | BEAN HULL BREAD ROLL | 152.0 | 352.0 | 5.3 | 12.9 | 51.7 | 22.0 | 1.8 |  |
| 2 | Breakfast | Rice Krispies | 40.0 | 153.0 | 0.4 | 2.3 | 36.5 | 0.3 | 0.4 |  |
| 2 | Breakfast | Jam | 19.0 | 50.6 | 0.0 | 0.1 | 13.1 | 0.0 | 0.0 |  |
| 2 | Breakfast | Tesco Sunflower spread | 10.0 | 49.1 | 5.5 | 0.0 | 0.1 | 0.0 | 0.1 |  |
| 2 | Breakfast | Orange juice, unsweetened | 191.0 | 69.9 | 0.0 | 1.7 | 16.4 | 0.0 | 0.0 |  |
| 2 | Breakfast | Semi-skimmed milk, pasteurised | 199.8 | 94.5 | 3.4 | 7.0 | 9.4 | 0.0 | 0.2 |  |
| 2 | Lunch | BEAN HULL BREAD ROLL | 152.0 | 352.0 | 5.3 | 12.9 | 51.7 | 22.0 | 1.8 |  |
| 2 | Lunch | Heinz potato and leek soup | 400.0 | 190.0 | 7.2 | 3.2 | 28.4 | 2.4 | 2.4 |  |
| 2 | Lunch | Tesco Sunflower spread | 9.6 | 47.1 | 5.3 | 0.0 | 0.1 | 0.0 | 0.1 |  |
| 2 | Lunch | Cheese, Cheddar-type, reduced fat | 19.0 | 59.3 | 4.2 | 5.3 | 0.2 | 0.0 | 0.3 |  |
| 2 | Lunch | Tesco Wafer Thin Honey Roast Ham | 16.0 | 19.1 | 0.4 | 3.5 | 0.3 | 0.1 | 0.3 |  |
| 2 | Lunch | walkers ready salted crisps | 25.0 | 133.0 | 8.0 | 1.5 | 14.2 | 1.1 | 0.4 |  |
| 2 | Dinner | chocolate, milk | 30.0 | 157.1 | 9.3 | 2.2 | 16.8 | 0.7 | 0.1 |  |
| 2 | Dinner | Tesco chicken curry with rice | 550.0 | 821.1 | 30.3 | 39.6 | 93.0 | 7.2 | 1.9 |  |
| **TOTAL (Day 2)** | | | **1813.4** | **2500.0** | **84.6** | **92.2** | **331.7** | **55.6** | **9.8** |  |
| 3 | Breakfast | BEAN HULL BREAD ROLL | 152.0 | 352.0 | 5.3 | 12.9 | 51.7 | 22.0 | 1.8 |  |
| 3 | Breakfast | Special K | 49.7 | 192.9 | 0.7 | 4.5 | 42.8 | 2.2 | 0.5 |  |
| 3 | Breakfast | Tesco Sunflower spread | 23.6 | 115.8 | 13.0 | 0.0 | 0.2 | 0.0 | 0.3 |  |
| 3 | Breakfast | Jam | 26.5 | 70.6 | 0.0 | 0.2 | 18.3 | 0.0 | 0.0 |  |
| 3 | Breakfast | Orange juice, unsweetened | 444.6 | 162.6 | 0.0 | 4.0 | 38.2 | 0.0 | 0.0 |  |
| 3 | Breakfast | Semi-skimmed milk, pasteurised | 134.0 | 63.3 | 2.3 | 4.7 | 6.3 | 0.0 | 0.1 |  |
| 3 | Lunch | BEAN HULL BREAD ROLL | 152.0 | 352.0 | 5.3 | 12.9 | 51.7 | 22.0 | 1.8 |  |
| 3 | Lunch | Cucumber, raw | 45.0 | 6.9 | 0.3 | 0.5 | 0.5 | 0.3 | 0.0 |  |
| 3 | Lunch | Mayonnaise reduced fat | 38.4 | 109.1 | 10.8 | 0.4 | 3.1 | 0.0 | 0.0 |  |
| 3 | Lunch | tuna in brine | 30.0 | 33.0 | 0.3 | 7.5 | 0.0 | 0.0 | 0.2 |  |
| 3 | Lunch | walkers ready salted crisps | 25.0 | 133.0 | 8.0 | 1.5 | 14.2 | 1.1 | 0.4 |  |
| 3 | Dinner | Tesco finest cottage pie | 570.0 | 635.5 | 22.8 | 38.8 | 69.5 | 5.1 | 4.6 |  |
| 3 | Dinner | Frozen peas cooked | 33.0 | 27.5 | 0.3 | 1.9 | 3.6 | 1.8 | 0.0 |  |
| 3 | Dinner | Tesco individual strawberry cheesecake | 100.0 | 270.7 | 15.5 | 2.9 | 31.5 | 0.6 | 0.2 |  |
| **TOTAL (Day 3)** | | | **1823.8** | **2500.0** | **84.6** | **92.5** | **331.6** | **55.1** | **9.9** |  |
| **Day** | **Meal** | **Food** | **Weight (g)** | **Energy (kcal)** | **Fat (g)** | **Protein (g)** | **CHO (g)** | **Fibre (g)** | **Salt (g)** |  |
| **Vegetarian Control Meals (2000kcal)** | | | | | | | | | | |
| 1 | Lunch | Tesco Mushroom risotto | 366.0 | 331.1 | 8.4 | 12.4 | 51.2 | 5.1 | 1.5 |  |
| 1 | Lunch | Mars bar | 43.2 | 186.2 | 7.2 | 1.9 | 29.9 | 0.0 | 0.2 |  |
| 1 | Dinner | CONTROL BREAD ROLL | 117.0 | 322.2 | 5.5 | 11.9 | 53.2 | 4.5 | 1.4 |  |
| 1 | Dinner | Tesco Macaroni and cheese pasta pot | 171.0 | 263.0 | 7.4 | 9.6 | 37.8 | 2.7 | 0.9 |  |
| 1 | Dinner | Cheese, Cheddar-type, reduced fat | 27.7 | 86.5 | 6.1 | 7.7 | 0.2 | 0.0 | 0.5 |  |
| 1 | Dinner | Tesco Sunflower spread | 12.4 | 60.8 | 6.8 | 0.0 | 0.1 | 0.0 | 0.2 |  |
| 1 | Dinner | Semi-skimmed milk, pasteurised | 477.7 | 225.8 | 8.1 | 16.7 | 22.5 | 0.0 | 0.5 |  |
| 1 | Dinner | double cream | 17.0 | 83.0 | 9.1 | 0.3 | 0.3 | 0.0 | 0.0 |  |
| 1 | Dinner | Digestive biscuit, plain | 16.0 | 76.7 | 3.6 | 1.1 | 10.3 | 0.6 | 0.2 |  |
| 1 | Dinner | Tesco peaches in light syrup | 22.9 | 10.5 | 0.0 | 0.1 | 2.6 | 0.2 | 0.0 |  |
| **TOTAL (Day 1)** | | | **1412.9** | **2000.0** | **67.8** | **73.8** | **278.5** | **17.7** | **6.7** |  |
| 2 | Breakfast | CONTROL BREAD ROLL | 117.0 | 322.2 | 5.5 | 11.9 | 53.2 | 4.5 | 1.4 |  |
| 2 | Breakfast | Rice Krispies | 30.0 | 114.7 | 0.3 | 1.7 | 27.4 | 0.2 | 0.3 |  |
| 2 | Breakfast | jam | 15.0 | 40.0 | 0.0 | 0.1 | 10.4 | 0.0 | 0.0 |  |
| 2 | Breakfast | Tesco Sunflower spread | 6.8 | 33.4 | 3.7 | 0.0 | 0.1 | 0.0 | 0.1 |  |
| 2 | Breakfast | Orange juice, unsweetened | 179.9 | 65.8 | 0.0 | 1.6 | 15.5 | 0.0 | 0.0 |  |
| 2 | Breakfast | Semi-skimmed milk, pasteurised | 347.0 | 164.0 | 5.9 | 12.1 | 16.3 | 0.0 | 0.4 |  |
| 2 | Lunch | CONTROL BREAD ROLL | 117.0 | 322.2 | 5.5 | 11.9 | 53.2 | 4.5 | 1.4 |  |
| 2 | Lunch | Tesco Sunflower spread | 7.7 | 37.8 | 4.2 | 0.0 | 0.1 | 0.0 | 0.1 |  |
| 2 | Lunch | Cheese, Cheddar-type, reduced fat | 60.0 | 187.3 | 13.3 | 16.7 | 0.5 | 0.0 | 1.1 |  |
| 2 | Lunch | Coleslaw Tesco | 40.0 | 67.7 | 6.3 | 0.4 | 2.4 | 0.8 | 0.2 |  |
| 2 | Lunch | walkers ready salted crisps | 25.0 | 133.0 | 8.0 | 1.5 | 14.2 | 1.1 | 0.4 |  |
| 2 | Dinner | Quorn Tantalising tikka masala | 375.0 | 396.0 | 6.8 | 13.5 | 67.9 | 11.3 | 1.8 |  |
| 2 | Dinner | chocolate, milk | 27.0 | 141.4 | 8.4 | 2.0 | 15.1 | 0.6 | 0.1 |  |
| **TOTAL (Day 2)** | | | **1347.4** | **2000.0** | **67.9** | **73.5** | **275.9** | **23.0** | **7.2** |  |
| 3 | Breakfast | CONTROL BREAD ROLL | 117.0 | 322.2 | 5.5 | 11.9 | 53.2 | 4.5 | 1.4 |  |
| 3 | Breakfast | Special K | 35.0 | 135.8 | 0.5 | 3.2 | 30.1 | 1.6 | 0.4 |  |
| 3 | Breakfast | Tesco Sunflower spread | 10.7 | 52.5 | 5.9 | 0.0 | 0.1 | 0.0 | 0.1 |  |
| 3 | Breakfast | Jam | 20.0 | 53.3 | 0.0 | 0.1 | 13.8 | 0.0 | 0.0 |  |
| 3 | Breakfast | Orange juice, unsweetened | 210.0 | 76.8 | 0.0 | 1.9 | 18.1 | 0.0 | 0.0 |  |
| 3 | Breakfast | Semi-skimmed milk, pasteurised | 333.0 | 157.4 | 5.7 | 11.7 | 15.7 | 0.0 | 0.4 |  |
| 3 | Lunch | CONTROL BREAD ROLL | 117.0 | 322.2 | 5.5 | 11.9 | 53.2 | 4.5 | 1.4 |  |
| 3 | Lunch | Cucumber, raw | 28.4 | 4.3 | 0.2 | 0.3 | 0.3 | 0.2 | 0.0 |  |
| 3 | Lunch | Mayonnaise reduced fat | 28.7 | 81.6 | 8.1 | 0.3 | 2.4 | 0.0 | 0.0 |  |
| 3 | Lunch | Boiled egg | 101.0 | 143.7 | 9.7 | 14.2 | 0.0 | 0.0 | 0.4 |  |
| 3 | Lunch | walkers ready salted crisps | 25.0 | 133.0 | 8.0 | 1.5 | 14.2 | 1.1 | 0.4 |  |
| 3 | Dinner | Quorn cottage pie | 300.0 | 237.7 | 3.0 | 11.4 | 39.6 | 6.9 | 2.1 |  |
| 3 | Dinner | Frozen peas cooked | 41.0 | 34.1 | 0.4 | 2.3 | 4.4 | 2.3 | 0.0 |  |
| 3 | Dinner | Tesco individual strawberry cheesecake | 100.0 | 270.7 | 15.5 | 2.9 | 31.5 | 0.6 | 0.2 |  |
| **TOTAL (Day 3)** | | | **1466.8** | **2000.0** | **67.9** | **73.6** | **276.4** | **21.7** | **6.7** |  |
| **Day** | **Meal** | **Food** | **Weight (g)** | **Energy (kcal)** | **Fat (g)** | **Protein (g)** | **CHO (g)** | **Fibre (g)** | **Salt (g)** |  |
| **Non-Vegetarian Control Meals (2000 kcal)** | | | | | | | | | | |
| 1 | Lunch | Tesco Mushroom risotto | 325.00 | 293.98 | 7.48 | 11.05 | 45.50 | 4.55 | 1.30 |  |
| 1 | Lunch | Tesco ready to eat flame grilled chicken mini fillet | 64.20 | 69.51 | 0.71 | 14.83 | 0.77 | 0.00 | 0.32 |  |
| 1 | Lunch | Mars bar | 51.00 | 219.80 | 8.52 | 2.24 | 35.34 | 0.00 | 0.21 |  |
| 1 | Dinner | CONTROL BREAD ROLL | 117.00 | 322.17 | 5.52 | 11.91 | 53.15 | 4.53 | 1.40 |  |
| 1 | Dinner | Tesco Macaroni and cheese pasta pot | 198.60 | 305.50 | 8.54 | 11.12 | 43.89 | 3.18 | 0.99 |  |
| 1 | Dinner | Tesco Sunflower spread | 14.00 | 68.70 | 7.70 | 0.01 | 0.13 | 0.00 | 0.18 |  |
| 1 | Dinner | Semi-skimmed milk, pasteurised | 252.10 | 119.17 | 4.29 | 8.82 | 11.85 | 0.00 | 0.27 |  |
| 1 | Dinner | double cream | 29.70 | 145.04 | 15.95 | 0.48 | 0.50 | 0.00 | 0.02 |  |
| 1 | Dinner | Digestive biscuit, plain | 16.00 | 76.67 | 3.57 | 1.07 | 10.34 | 0.61 | 0.16 |  |
| 1 | Dinner | Tesco peaches in light syrup | 58.30 | 26.71 | 0.00 | 0.17 | 6.53 | 0.52 | 0.00 |  |
| **TOTAL (Day 1)** | | | **1267.90** | **2000.02** | **67.79** | **73.78** | **278.41** | **17.92** | **6.28** |  |
| 2 | Breakfast | CONTROL BREAD ROLL | 117.00 | 322.17 | 5.52 | 11.91 | 53.15 | 4.53 | 1.40 |  |
| 2 | Breakfast | Rice Krispies | 34.50 | 131.95 | 0.35 | 1.97 | 31.46 | 0.24 | 0.32 |  |
| 2 | Breakfast | Jam | 19.00 | 50.65 | 0.00 | 0.11 | 13.11 | 0.00 | 0.01 |  |
| 2 | Breakfast | Tesco Sunflower spread | 10.00 | 49.07 | 5.50 | 0.01 | 0.09 | 0.00 | 0.13 |  |
| 2 | Breakfast | Orange juice, unsweetened | 152.40 | 55.75 | 0.00 | 1.37 | 13.11 | 0.00 | 0.00 |  |
| 2 | Breakfast | Semi-skimmed milk, pasteurised | 135.00 | 63.82 | 2.30 | 4.73 | 6.35 | 0.00 | 0.15 |  |
| 2 | Lunch | CONTROL BREAD ROLL | 117.00 | 322.17 | 5.52 | 11.91 | 53.15 | 4.53 | 1.40 |  |
| 2 | Lunch | Heinz potato and leek soup | 180.00 | 85.52 | 3.24 | 1.44 | 12.78 | 1.08 | 1.08 |  |
| 2 | Lunch | Tesco Sunflower spread | 7.70 | 37.78 | 4.24 | 0.01 | 0.07 | 0.00 | 0.10 |  |
| 2 | Lunch | Cheese, Cheddar-type, reduced fat | 17.30 | 54.00 | 3.82 | 4.83 | 0.14 | 0.00 | 0.31 |  |
| 2 | Lunch | Tesco Wafer Thin Honey Roast Ham | 11.70 | 13.96 | 0.30 | 2.53 | 0.22 | 0.07 | 0.23 |  |
| 2 | Lunch | walkers ready salted crisps | 25.00 | 133.01 | 7.98 | 1.53 | 14.15 | 1.08 | 0.35 |  |
| 2 | Dinner | chocolate, milk | 20.00 | 104.75 | 6.22 | 1.46 | 11.20 | 0.46 | 0.04 |  |
| 2 | Dinner | Tesco chicken curry with rice | 414.00 | 618.03 | 22.77 | 29.81 | 69.97 | 5.38 | 1.45 |  |
| **TOTAL (Day 2)** | | | **1260.60** | **2000.04** | **67.75** | **73.60** | **278.95** | **17.36** | **6.99** |  |
| 3 | Breakfast | CONTROL BREAD ROLL | 117.00 | 322.17 | 5.52 | 11.91 | 53.15 | 4.53 | 1.40 |  |
| 3 | Breakfast | Special K | 35.00 | 135.82 | 0.53 | 3.15 | 30.14 | 1.58 | 0.35 |  |
| 3 | Breakfast | Tesco Sunflower spread | 11.10 | 54.47 | 6.11 | 0.01 | 0.10 | 0.00 | 0.14 |  |
| 3 | Breakfast | Jam | 20.70 | 55.18 | 0.00 | 0.12 | 14.28 | 0.00 | 0.02 |  |
| 3 | Breakfast | Orange juice, unsweetened | 260.00 | 95.11 | 0.00 | 2.34 | 22.36 | 0.00 | 0.01 |  |
| 3 | Breakfast | Semi-skimmed milk, pasteurised | 101.00 | 47.75 | 1.72 | 3.54 | 4.75 | 0.00 | 0.11 |  |
| 3 | Lunch | CONTROL BREAD ROLL | 117.00 | 322.17 | 5.52 | 11.91 | 53.15 | 4.53 | 1.40 |  |
| 3 | Lunch | Cucumber, raw | 25.00 | 3.83 | 0.15 | 0.25 | 0.30 | 0.18 | 0.00 |  |
| 3 | Lunch | Mayonnaise reduced fat | 30.00 | 85.26 | 8.43 | 0.30 | 2.46 | 0.00 | 0.00 |  |
| 3 | Lunch | tuna in brine | 29.00 | 31.93 | 0.29 | 7.22 | 0.00 | 0.00 | 0.21 |  |
| 3 | Lunch | walkers ready salted crisps | 25.00 | 133.01 | 7.98 | 1.53 | 14.15 | 1.08 | 0.35 |  |
| 3 | Dinner | Tesco finest cottage pie | 390.00 | 434.78 | 15.60 | 26.52 | 47.58 | 3.51 | 3.12 |  |
| 3 | Dinner | Frozen peas cooked | 40.00 | 33.28 | 0.36 | 2.28 | 4.32 | 2.24 | 0.00 |  |
| 3 | Dinner | Tesco individual strawberry cheesecake | 100.00 | 270.72 | 15.50 | 2.90 | 31.50 | 0.60 | 0.17 |  |
| **TOTAL (Day 3)** | | | **1300.80** | **2000.02** | **67.70** | **73.98** | **278.24** | **18.23** | **7.29** |  |
| **Day** | **Meal** | **Food** | **Weight (g)** | **Energy (kcal)** | **Fat (g)** | **Protein (g)** | **CHO (g)** | **Fibre (g)** | **Salt (g)** |  |
| **Vegetarian Control Meals (2500kcal)** | | | | | | | | | | |
| 1 | Lunch | Tesco Mushroom risotto | 366.0 | 331.1 | 8.4 | 12.4 | 51.2 | 5.1 | 1.5 |  |
| 1 | Lunch | Mars bar | 43.2 | 186.2 | 7.2 | 1.9 | 29.9 | 0.0 | 0.2 |  |
| 1 | Dinner | CONTROL BREAD ROLL | 117.0 | 322.2 | 5.5 | 11.9 | 53.2 | 4.5 | 1.4 |  |
| 1 | Dinner | Tesco Macaroni and cheese pasta pot | 314.0 | 483.0 | 13.5 | 17.6 | 69.4 | 5.0 | 1.6 |  |
| 1 | Dinner | Cheese, Cheddar-type, reduced fat | 58.4 | 182.3 | 12.9 | 16.3 | 0.5 | 0.0 | 1.1 |  |
| 1 | Dinner | Tesco Sunflower spread | 11.2 | 55.0 | 6.2 | 0.0 | 0.1 | 0.0 | 0.1 |  |
| 1 | Dinner | Semi-skimmed milk, pasteurised | 478.8 | 226.3 | 8.1 | 16.8 | 22.5 | 0.0 | 0.5 |  |
| 1 | Dinner | double cream | 19.0 | 92.8 | 10.2 | 0.3 | 0.3 | 0.0 | 0.0 |  |
| 1 | Dinner | Digestive biscuit, plain | 32.0 | 153.3 | 7.1 | 2.1 | 20.7 | 1.2 | 0.3 |  |
| 1 | Dinner | Tesco peaches in light syrup | 264.6 | 121.2 | 0.0 | 0.8 | 29.6 | 2.4 | 0.0 |  |
| **TOTAL (Day 1)** | | | **1846.2** | **2500.0** | **84.7** | **92.2** | **347.8** | **22.8** | **8.1** |  |
| 2 | Breakfast | CONTROL BREAD ROLL | 117.0 | 322.2 | 5.5 | 11.9 | 53.2 | 4.5 | 1.4 |  |
| 2 | Breakfast | Rice Krispies | 40.0 | 153.0 | 0.4 | 2.3 | 36.5 | 0.3 | 0.4 |  |
| 2 | Breakfast | jam | 19.4 | 51.7 | 0.0 | 0.1 | 13.4 | 0.0 | 0.0 |  |
| 2 | Breakfast | Tesco Sunflower spread | 10.0 | 49.1 | 5.5 | 0.0 | 0.1 | 0.0 | 0.1 |  |
| 2 | Breakfast | Orange juice, unsweetened | 383.0 | 140.1 | 0.0 | 3.4 | 32.9 | 0.0 | 0.0 |  |
| 2 | Breakfast | Semi-skimmed milk, pasteurised | 472.8 | 223.5 | 8.0 | 16.5 | 22.2 | 0.0 | 0.5 |  |
| 2 | Lunch | CONTROL BREAD ROLL | 117.0 | 322.2 | 5.5 | 11.9 | 53.2 | 4.5 | 1.4 |  |
| 2 | Lunch | Tesco Sunflower spread | 13.0 | 63.8 | 7.2 | 0.0 | 0.1 | 0.0 | 0.2 |  |
| 2 | Lunch | Cheese, Cheddar-type, reduced fat | 80.0 | 249.7 | 17.7 | 22.3 | 0.6 | 0.0 | 1.4 |  |
| 2 | Lunch | Coleslaw Tesco | 50.0 | 84.6 | 7.9 | 0.5 | 3.0 | 1.0 | 0.3 |  |
| 2 | Lunch | walkers ready salted crisps | 25.0 | 133.0 | 8.0 | 1.5 | 14.2 | 1.1 | 0.4 |  |
| 2 | Dinner | Quorn Tantalising tikka masala | 545.0 | 575.5 | 9.8 | 19.6 | 98.6 | 16.4 | 2.6 |  |
| 2 | Dinner | chocolate, milk | 30.0 | 157.1 | 9.3 | 2.2 | 16.8 | 0.7 | 0.1 |  |
| **TOTAL (Day 2)** | | | **1902.2** | **2500.0** | **84.8** | **92.4** | **344.7** | **28.4** | **8.7** |  |
| 3 | Breakfast | CONTROL BREAD ROLL | 117.0 | 322.2 | 5.5 | 11.9 | 53.2 | 4.5 | 1.4 |  |
| 3 | Breakfast | Special K | 48.0 | 186.3 | 0.7 | 4.3 | 41.3 | 2.2 | 0.5 |  |
| 3 | Breakfast | Tesco Sunflower spread | 23.3 | 114.3 | 12.8 | 0.0 | 0.2 | 0.0 | 0.3 |  |
| 3 | Breakfast | Jam | 28.0 | 74.6 | 0.0 | 0.2 | 19.3 | 0.0 | 0.0 |  |
| 3 | Breakfast | Orange juice, unsweetened | 382.9 | 140.1 | 0.0 | 3.4 | 32.9 | 0.0 | 0.0 |  |
| 3 | Breakfast | Semi-skimmed milk, pasteurised | 397.0 | 187.7 | 6.7 | 13.9 | 18.7 | 0.0 | 0.4 |  |
| 3 | Lunch | CONTROL BREAD ROLL | 117.0 | 322.2 | 5.5 | 11.9 | 53.2 | 4.5 | 1.4 |  |
| 3 | Lunch | Cucumber, raw | 28.4 | 4.3 | 0.2 | 0.3 | 0.3 | 0.2 | 0.0 |  |
| 3 | Lunch | Mayonnaise reduced fat | 40.0 | 113.7 | 11.2 | 0.4 | 3.3 | 0.0 | 0.0 |  |
| 3 | Lunch | Boiled egg | 131.0 | 186.4 | 12.6 | 18.5 | 0.0 | 0.0 | 0.5 |  |
| 3 | Lunch | walkers ready salted crisps | 25.0 | 133.0 | 8.0 | 1.5 | 14.2 | 1.1 | 0.4 |  |
| 3 | Dinner | Quorn cottage pie | 550.0 | 435.8 | 5.5 | 20.9 | 72.6 | 12.7 | 3.9 |  |
| 3 | Dinner | Frozen peas cooked | 41.0 | 34.1 | 0.4 | 2.3 | 4.4 | 2.3 | 0.0 |  |
| 3 | Dinner | Tesco individual strawberry cheesecake | 100.0 | 270.7 | 15.5 | 2.9 | 31.5 | 0.6 | 0.2 |  |
| **TOTAL (Day 3)** | | | **2028.6** | **2500.0** | **84.7** | **92.5** | **345.1** | **28.0** | **8.9** |  |
| **Day** | **Meal** | **Food** | **Weight (g)** | **Energy (kcal)** | **Fat (g)** | **Protein (g)** | **CHO (g)** | **Fibre (g)** | **Salt (g)** |  |
| **Non-Vegetarian Control Meals (2500 kcal)** | | | | | | | | | | |
| 1 | Lunch | Tesco chicken and asparagus Risotto | 358.0 | 334.9 | 5.0 | 25.8 | 45.8 | 5.4 | 1.1 |  |
| 1 | Lunch | Mars bar | 51.0 | 219.8 | 8.5 | 2.2 | 35.3 | 0.0 | 0.2 |  |
| 1 | Dinner | CONTROL BREAD ROLL | 117.0 | 322.2 | 5.5 | 11.9 | 53.2 | 4.5 | 1.4 |  |
| 1 | Dinner | Tesco Macaroni and cheese pasta pot | 470.1 | 723.1 | 20.2 | 26.3 | 103.9 | 7.5 | 2.4 |  |
|  | Dinner | Tesco Sunflower spread | 16.6 | 81.5 | 9.1 | 0.0 | 0.1 | 0.0 | 0.2 |  |
| 1 | Dinner | Semi-skimmed milk, pasteurised | 339.0 | 160.3 | 5.8 | 11.9 | 15.9 | 0.0 | 0.4 |  |
| 1 | Dinner | double cream | 40.0 | 195.3 | 21.5 | 0.6 | 0.7 | 0.0 | 0.0 |  |
| 1 | Dinner | Digestive biscuit, plain | 16.0 | 76.7 | 3.6 | 1.1 | 10.3 | 0.6 | 0.2 |  |
| 1 | Dinner | Tesco peaches in light syrup | 104.8 | 48.0 | 0.0 | 0.3 | 11.7 | 0.9 | 0.0 |  |
| **TOTAL (Day 1)** | | | **1654.5** | **2500.0** | **84.7** | **92.2** | **347.5** | **23.5** | **7.2** |  |
| 2 | Breakfast | CONTROL BREAD ROLL | 117.0 | 322.2 | 5.5 | 11.9 | 53.2 | 4.5 | 1.4 |  |
| 2 | Breakfast | Rice Krispies | 46.0 | 175.9 | 0.5 | 2.6 | 42.0 | 0.3 | 0.4 |  |
| 2 | Breakfast | Jam | 20.0 | 53.3 | 0.0 | 0.1 | 13.8 | 0.0 | 0.0 |  |
| 2 | Breakfast | Tesco Sunflower spread | 8.5 | 41.7 | 4.7 | 0.0 | 0.1 | 0.0 | 0.1 |  |
| 2 | Breakfast | Orange juice, unsweetened | 281.0 | 102.8 | 0.0 | 2.5 | 24.2 | 0.0 | 0.0 |  |
| 2 | Breakfast | Semi-skimmed milk, pasteurised | 199.1 | 94.1 | 3.4 | 7.0 | 9.4 | 0.0 | 0.2 |  |
| 2 | Lunch | CONTROL BREAD ROLL | 117.0 | 322.2 | 5.5 | 11.9 | 53.2 | 4.5 | 1.4 |  |
| 2 | Lunch | Heinz potato and leek soup | 400.0 | 190.0 | 7.2 | 3.2 | 28.4 | 2.4 | 2.4 |  |
| 2 | Lunch | Tesco Sunflower spread | 10.0 | 49.1 | 5.5 | 0.0 | 0.1 | 0.0 | 0.1 |  |
| 2 | Lunch | Cheese, Cheddar-type, reduced fat | 19.8 | 61.8 | 4.4 | 5.5 | 0.2 | 0.0 | 0.4 |  |
| 2 | Lunch | Tesco Wafer Thin Honey Roast Ham | 20.0 | 23.9 | 0.5 | 4.3 | 0.4 | 0.1 | 0.4 |  |
| 2 | Lunch | walkers ready salted crisps | 25.0 | 133.0 | 8.0 | 1.5 | 14.2 | 1.1 | 0.4 |  |
| 2 | Dinner | chocolate, milk | 30.0 | 157.1 | 9.3 | 2.2 | 16.8 | 0.7 | 0.1 |  |
| 2 | Dinner | Tesco chicken curry with rice | 550.0 | 821.1 | 30.3 | 39.6 | 93.0 | 7.2 | 1.9 |  |
| **TOTAL (Day 2)** | | | **1843.4** | **2500.0** | **84.7** | **92.4** | **348.6** | **20.8** | **9.2** |  |
| 3 | Breakfast | CONTROL BREAD ROLL | 117.0 | 322.2 | 5.5 | 11.9 | 53.2 | 4.5 | 1.4 |  |
| 3 | Breakfast | Special K | 72.1 | 279.8 | 1.1 | 6.5 | 62.1 | 3.2 | 0.7 |  |
| 3 | Breakfast | Tesco Sunflower spread | 26.0 | 127.6 | 14.3 | 0.0 | 0.2 | 0.0 | 0.3 |  |
| 3 | Breakfast | jam | 33.1 | 88.2 | 0.0 | 0.2 | 22.8 | 0.0 | 0.0 |  |
| 3 | Breakfast | Orange juice, unsweetened | 314.7 | 115.1 | 0.0 | 2.8 | 27.1 | 0.0 | 0.0 |  |
| 3 | Breakfast | Semi-skimmed milk, pasteurised | 140.0 | 66.2 | 2.4 | 4.9 | 6.6 | 0.0 | 0.2 |  |
| 3 | Lunch | CONTROL BREAD ROLL | 117.0 | 322.2 | 5.5 | 11.9 | 53.2 | 4.5 | 1.4 |  |
| 3 | Lunch | Cucumber, raw | 40.0 | 6.1 | 0.2 | 0.4 | 0.5 | 0.3 | 0.0 |  |
| 3 | Lunch | Mayonnaise reduced fat | 35.0 | 99.5 | 9.8 | 0.4 | 2.9 | 0.0 | 0.0 |  |
| 3 | Lunch | tuna in brine | 34.0 | 37.4 | 0.3 | 8.5 | 0.0 | 0.0 | 0.2 |  |
| 3 | Lunch | walkers ready salted crisps | 25.0 | 133.0 | 8.0 | 1.5 | 14.2 | 1.1 | 0.4 |  |
| 3 | Dinner | Tesco finest cottage pie | 530.0 | 590.9 | 21.2 | 36.0 | 64.7 | 4.8 | 4.2 |  |
| 3 | Dinner | Frozen peas cooked | 80.0 | 66.6 | 0.7 | 4.6 | 8.6 | 4.5 | 0.0 |  |
| 3 | Dinner | Tesco individual strawberry cheesecake | 100.0 | 270.7 | 15.5 | 2.9 | 31.5 | 0.6 | 0.2 |  |
| **TOTAL (Day 3)** | | | **1663.9** | **2500.0** | **84.6** | **92.5** | **347.4** | **23.5** | **9.1** |  |

^1^ All food was purchased from Tesco (Aberdeen, UK), unless otherwise stated.  ^2^ The daily macronutrient intakes for the 3 days were determined by trained staff using WinDiets Nutritional Analysis Software Suite (The Robert Gordon University, Aberdeen, UK) which is a computerised version of *McCance and Widdowson’s The Composition of Foods.*

**Supplementary Table 3.** Test meal ingredients and composition*

| **(g)** | **Bean Hull** | **Control** |
| --- | --- | --- |
| White bread flour (Allinson) | 75 | 75 |
| Broad bean hull powder (Askew and Barrett Ltd, UK) | 20 | - |
| Yeast (Allinson dry instant) | 2 | 2 |
| Salt (Saxa fine sea) | 1.8 | 1.4 |
| Fat (Trex) | 3.75 | 3.75 |
| Water (Lukewarm at 35-40 ^o^C) | 75 | 59 |
|  | **Spread** | |
| Raspberry jam (g) | 25 | |

*All ingredients were purchased by Tesco, UK unless otherwise stated.

**
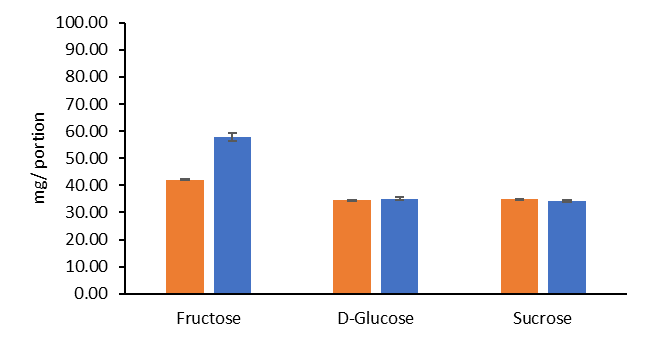
**

**Supplementary Figure 1**. Free sugar content of the bean hull (orange) and control (blue) bread rolls.

**
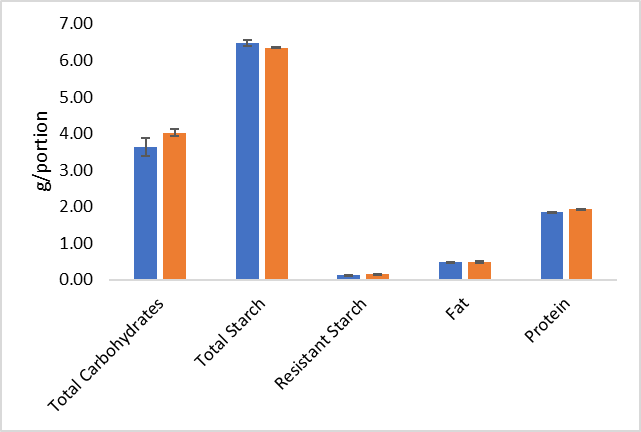
**

**Supplementary Figure 2.** Macronutrient composition of the bean hull (orange) and control (blue) bread rolls for one portion. CHO: Carbohydrates; Res. Starch: resistant starch

**Supplementary Table 4. Plant metabolites measured in the bean hull and control bread rolls. mg/portion consumed ± SD (n=3)**

|  | **mg/portion eaten** | **Control Bread** | | **Bean hull Bread** | |
| --- | --- | --- | --- | --- | --- |
|  |  | **Free** | **Bound** | **Free** | **Bound** |
| **Benzoic acid** | benzoic acid | 0 ± 0 | 0.45 ± 0.18 | 0.13 ± 0.13 | 0.64 ± 0.23 |
|  | salicylic acid | 0 ± 0 | 0.01 ± 0 | 0.02 ± 0 | 0.01 ± 0 |
|  | m-hydroxybenzoic acid | 0 ± 0 | 0 ± 0 | 0 ± 0 | 0 ± 0 |
|  | p-hydroxybenzoic acid | 0.03 ± 0 | 0.16 ± 0 | 0.1 ± 0.01 | 0.3 ± 0.01 |
|  | 2,3-dihydroxybenzoic acid | 0 ± 0 | 0 ± 0 | 0 ± 0 | 0.02 ± 0.02 |
|  | 2,4-dihydroxybenzoic acid | 0 ± 0 | 0 ± 0 | 0 ± 0 | 0 ± 0 |
|  | 2,5-dihydroxybenzoic acid | 0 ± 0 | 0 ± 0 | 0.04 ± 0 | 0.06 ± 0.04 |
|  | 2,6-dihydroxybenzoic acid | 0.01 ± 0 | 0 ± 0 | 0.01 ± 0 | 0 ± 0 |
|  | protocatechuic acid | 0.02 ± 0 | 0.01 ± 0.01 | 0.74 ± 0.04 | 2.05 ± 0.54 |
|  | 3,5-dihydroxybenzoic acid | 0 ± 0 | 0 ± 0 | 0 ± 0 | 0.01 ± 0.01 |
|  | o-anisic acid | 0 ± 0 | 0 ± 0 | 0 ± 0 | 0 ± 0 |
|  | m-anisic acid | 0 ± 0 | 0 ± 0 | 0 ± 0 | 0 ± 0 |
|  | p-anisic acid | 0 ± 0 | 0 ± 0 | 0 ± 0 | 0.02 ± 0.01 |
|  | gallic acid | 0 ± 0 | 0 ± 0 | 0.72 ± 0.04 | 0.96 ± 0.04 |
|  | vanillic acid | 0.05 ± 0.01 | 0.16 ± 0.05 | 0.09 ± 0.01 | 0.22 ± 0.04 |
|  | syringic acid | 0.02 ± 0 | 0.12 ± 0.01 | 0.02 ± 0 | 0.15 ± 0.01 |
|  | 3,4-dimethoxybenzoic acid | 0 ± 0 | 0 ± 0 | 0 ± 0 | 0 ± 0 |
| **Benzaldehydes** | p-hydroxybenzaldehyde | 0.03 ± 0.01 | 0.08 ± 0.04 | 0.01 ± 0 | 0.12 ± 0.05 |
|  | protocatachaldehyde | 0 ± 0 | 0 ± 0 | 0.33 ± 0.01 | 2.49 ± 0.81 |
|  | 3,4,5-trihydroxybenzaldehyde | 0 ± 0 | 0 ± 0 | 0.69 ± 0.03 | 3.14 ± 1.08 |
|  | vanillin | 0.02 ± 0 | 0.16 ± 0.03 | 0.02 ± 0 | 0.19 ± 0.04 |
|  | isovanillin | 0 ± 0 | 0 ± 0 | 0 ± 0 | 0 ± 0 |
|  | syringin | 0 ± 0 | 0.02 ± 0 | 0 ± 0 | 0.02 ± 0 |
|  | 3-methoxybenzaldehyde | 0 ± 0 | 0 ± 0 | 0 ± 0 | 0 ± 0 |
|  | 3,4-dimethoxybenzaldehyde | 0 ± 0 | 0 ± 0 | 0 ± 0 | 0 ± 0 |
|  | 3,4,5-trimethoxybenzaldehyde | 0 ± 0 | 0 ± 0 | 0 ± 0 | 0 ± 0 |
| **Cinnamic Acids** | cinnamic acid | 0 ± 0 | 0 ± 0 | 0.08 ± 0.01 | 0.01 ± 0.01 |
|  | o-coumaric acid | 0 ± 0 | 0 ± 0 | 0 ± 0 | 0 ± 0 |
|  | m-coumaric acid | 0 ± 0 | 0 ± 0 | 0 ± 0 | 0 ± 0 |
|  | p-coumaric acid | 0.01 ± 0 | 0.2 ± 0.11 | 0.09 ± 0.01 | 0.26 ± 0.13 |
|  | caffeic acid | 0.01 ± 0 | 0.07 ± 0.02 | 0.09 ± 0 | 0.11 ± 0.03 |
|  | ferulic acid | 0.2 ± 0.02 | 6.82 ± 4.49 | 0.23 ± 0.02 | 7.15 ± 4.69 |
|  | sinapic acid | 0.03 ± 0 | 0.53 ± 0.33 | 0.05 ± 0 | 0.69 ± 0.41 |
|  | 3-methoxycinnamic acid | 0 ± 0 | 0 ± 0 | 0 ± 0 | 0 ± 0 |
|  | 4-methoxycinnamic acid | 0 ± 0 | 0 ± 0 | 0 ± 0 | 0 ± 0 |
|  | 3,4-dimethoxycinnamic acid | 0 ± 0 | 0 ± 0 | 0 ± 0 | 0 ± 0 |
|  | 3,4,5-trimethoxycinnamic acid | 0 ± 0 | 0 ± 0 | 0 ± 0 | 0 ± 0 |
| **Phenylpropionic acids** | phenylpropionic acid | 0 ± 0 | 0 ± 0 | 0 ± 0 | 0 ± 0 |
|  | 2-hydroxyphenylpropionic acid | 0 ± 0 | 0 ± 0 | 0 ± 0 | 0 ± 0 |
|  | 3-hydroxyphenylpropionic acid | 0 ± 0 | 0 ± 0 | 0 ± 0 | 0 ± 0 |
|  | 4-hydroxyphenylpropionic acid | 0 ± 0 | 1.48 ± 0.31 | 0 ± 0 | 2 ± 0.33 |
|  | 3,4-dihydroxyphenylpropionic acid | 0 ± 0 | 0 ± 0 | 0 ± 0 | 0 ± 0 |
|  | 4-hydroxy-3-methoxyphenylpropionic acid | 0 ± 0 | 0.04 ± 0.01 | 0.02 ± 0 | 0.05 ± 0.04 |
|  | 3-methoxyphenylpropionic acid | 0 ± 0 | 0 ± 0 | 0 ± 0 | 0 ± 0 |
| **Benzenes** | phenol | 0 ± 0 | 0.27 ± 0.19 | 0 ± 0 | 0.36 ± 0.25 |
|  | 1,2-hydroxybenzene | 0.03 ± 0 | 0 ± 0 | 0 ± 0 | 0 ± 0 |
|  | 1,3-hydroxybenzene | 0 ± 0 | 0 ± 0 | 0 ± 0 | 0 ± 0 |
|  | 1,2,3-trihydroxybenzene | 0.06 ± 0.06 | 0 ± 0 | 0.06 ± 0.01 | 0 ± 0 |
| **Acetophenones** | 4-hydroxyacetophenone | 0 ± 0 | 0.02 ± 0 | 0 ± 0 | 0.02 ± 0 |
|  | 4-hydroxy-3-methoxyacetophenone | 0.01 ± 0 | 0.03 ± 0.01 | 0 ± 0 | 0.03 ± 0.01 |
|  | 4-hydroxy-3,5-dimethoxyacetophenone | 0 ± 0 | 0.05 ± 0.04 | 0 ± 0 | 0.06 ± 0.04 |
|  | 3,4-dimethoxyacetophenone | 0 ± 0 | 0 ± 0 | 0 ± 0 | 0 ± 0 |
|  | 3,4,5-trimethoxyacetophenone | 0 ± 0 | 0 ± 0 | 0 ± 0 | 0 ± 0 |
| **Phenylacetic acids** | phenylacetic acid | 0.04 ± 0 | 0.03 ± 0.01 | 0.11 ± 0.01 | 0.04 ± 0.01 |
|  | 3-hydroxyphenylacetic acid | 0 ± 0 | 0 ± 0 | 0 ± 0 | 0 ± 0 |
|  | 4-hydroxyphenylacetic acid | 0.01 ± 0.02 | 0 ± 0 | 0.09 ± 0.01 | 0.18 ± 0.01 |
|  | 3,4-dihydroxyphenylacetic acid | 0 ± 0 | 0 ± 0 | 0.06 ± 0.01 | 0.17 ± 0.07 |
|  | 4-hydroxy-3-methoxyphenylacetic acid | 0 ± 0 | 0 ± 0 | 0 ± 0 | 0 ± 0 |
|  | 4-methoxyphenylacetic acid | 0 ± 0 | 0 ± 0 | 0 ± 0 | 0 ± 0 |
| **Mandelic Acids** | mandelic acid | 0 ± 0 | 0 ± 0 | 0 ± 0 | 0 ± 0 |
|  | 3-hydroxymandelic acid | 0 ± 0 | 0 ± 0 | 0.15 ± 0.01 | 0.5 ± 0.06 |
|  | 4-hydroxymandelic acid | 0 ± 0 | 0.01 ± 0.01 | 0 ± 0 | 0 ± 0 |
|  | 3,4-dihydroxymandelic acid | 0 ± 0 | 0 ± 0 | 0.13 ± 0.02 | 0.29 ± 0.09 |
|  | 4-hydroxy-3-methoxymandelic acid | 0 ± 0 | 0 ± 0 | 0 ± 0 | 0 ± 0 |
| **Phenypyruvic Acids** | phenylpyruvic acid | 0.25 ± 0.04 | 0 ± 0 | 0.21 ± 0.03 | 0.06 ± 0.02 |
|  | 4-hydroxyphenylpyruvic acid | 0 ± 0 | 0 ± 0 | 0 ± 0 | 0 ± 0 |
| **Phenyllactic Acids** | phenyllactic acid | 0.13 ± 0.02 | 0 ± 0 | 0.17 ± 0.02 | 0.01 ± 0 |
|  | 4-hydroxyphenyllactic acid | 0.06 ± 0.01 | 0 ± 0 | 0.09 ± 0 | 0.02 ± 0.01 |
| **Phenolics Others** | anthranilic acid | 0 ± 0 | 0.01 ± 0 | 0 ± 0 | 0.02 ± 0.01 |
|  | quinadilic acid | 0 ± 0 | 0 ± 0 | 0 ± 0 | 0 ± 0 |
|  | chlorogenic acid | 0 ± 0 | 0 ± 0 | 0 ± 0 | 0 ± 0 |
|  | 0-hydroxyhippuric acid | 0 ± 0 | 0 ± 0 | 0 ± 0 | 0 ± 0 |
|  | ethylferulate | 0.03 ± 0 | 0 ± 0 | 0.02 ± 0 | 0 ± 0 |
|  | p-cresol | 0 ± 0 | 0 ± 0 | 0 ± 0 | 0 ± 0 |
|  | 4-ethylphenol | 0 ± 0 | 0 ± 0 | 0 ± 0 | 0 ± 0 |
|  | 4-methylcatechol | 0 ± 0 | 0 ± 0 | 0.01 ± 0.02 | 0.01 ± 0.01 |
|  | Tyrosol | 0.29 ± 0.05 | 0 ± 0 | 0.26 ± 0.01 | 0 ± 0 |
| **Phenolic Dimers** | Ellagic Acid | 0 ± 0 | 0 ± 0 | 0 ± 0 | 0 ± 0 |
|  | Ferulic Dimer 5-5 | 0 ± 0 | 0.43 ± 0.29 | 0 ± 0 | 0.4 ± 0.28 |
|  | Ferulic Dimer 8-8C | 0 ± 0 | 0 ± 0 | 0 ± 0 | 0.21 ± 0.15 |
|  | Ferulic Dimer 8-5C | 0 ± 0 | 0.28 ± 0.2 | 0 ± 0 | 0 ± 0 |
|  | Resveratrol | 0 ± 0 | 0 ± 0 | 0.02 ± 0 | 0 ± 0 |
| **Indoles** | Indole | 0.06 ± 0.01 | 0.02 ± 0.01 | 0.01 ± 0.01 | 0.02 ± 0.01 |
|  | Indole-3 Acetic acid | 0.07 ± 0.01 | 0.04 ± 0.03 | 0.02 ± 0 | 0.04 ± 0.03 |
|  | Indole-acrylic acid | 0 ± 0 | 0 ± 0 | 0 ± 0 | 0 ± 0 |
|  | Indole-3 Propionic acid | 0 ± 0 | 0 ± 0 | 0 ± 0 | 0 ± 0 |
|  | Indole-3-Carbinol | 0 ± 0 | 0 ± 0 | 0 ± 0 | 0 ± 0 |
|  | Indole-3 Carboxylic acid | 0.01 ± 0 | 0.01 ± 0.01 | 0.01 ± 0 | 0.02 ± 0.01 |
|  | Indole-3-Pyruvic acid | 0 ± 0 | 0 ± 0 | 0 ± 0 | 0 ± 0 |
|  | lndole-methyl | 0 ± 0 | 0 ± 0 | 0 ± 0 | 0 ± 0 |
|  | Indole-lactic acid | 0.03 ± 0 | 0 ± 0 | 0.02 ± 0 | 0 ± 0 |
|  | 3-Indoleacetonitrile | 0 ± 0 | 0 ± 0 | 0 ± 0 | 0 ± 0 |
|  | Indole-3-Carboxaldehyde | 0.07 ± 0.01 | 0.02 ± 0.01 | 0.02 ± 0 | 0.02 ± 0.02 |
| **Flavanoids/Coumarins/Isoflavonoids** | Coumarin | 0 ± 0 | 0 ± 0 | 0.03 ± 0 | 0 ± 0 |
|  | Psoralen | 0 ± 0 | 0 ± 0 | 0 ± 0 | 0 ± 0 |
|  | 8-methylpsoralen | 0 ± 0 | 0 ± 0 | 0 ± 0 | 0 ± 0 |
|  | Bergapten | 0 ± 0 | 0 ± 0 | 0 ± 0 | 0 ± 0 |
|  | Tangeretin | 0.01 ± 0 | 0.02 ± 0 | 0.01 ± 0 | 0.02 ± 0 |
|  | Coumesterol | 0 ± 0 | 0 ± 0 | 0 ± 0 | 0 ± 0 |
|  | Catechin | 0 ± 0 | 0 ± 0 | 3.43 ± 0.5 | 1.62 ± 1.15 |
|  | Epicatechin | 0 ± 0 | 0 ± 0 | 3.36 ± 0.09 | 0.25 ± 0.18 |
|  | Gallocatechin | 0 ± 0 | 0 ± 0 | 1.77 ± 0.24 | 0.41 ± 0.29 |
|  | Epigallocatechin | 0 ± 0 | 0 ± 0 | 0.5 ± 0.06 | 0.07 ± 0.05 |
|  | Epigallocatechin Gallate | 0 ± 0 | 0 ± 0 | 0 ± 0 | 0 ± 0 |
|  | Isoliquiritigenin | 0 ± 0 | 0 ± 0 | 0.02 ± 0 | 0.01 ± 0.01 |
|  | Phloretin | 0 ± 0 | 0 ± 0 | 0 ± 0 | 0 ± 0 |
|  | Imperatorin | 0 ± 0 | 0.01 ± 0 | 0.01 ± 0 | 0.01 ± 0 |
|  | Eriocitrin | 0 ± 0 | 0 ± 0 | 0 ± 0 | 0 ± 0 |
|  | Naringenin | 0 ± 0 | 0 ± 0 | 0.01 ± 0 | 0 ± 0 |
|  | Naringin | 0 ± 0 | 0 ± 0 | 0 ± 0 | 0 ± 0 |
|  | Hesperitin | 0 ± 0 | 0 ± 0 | 0 ± 0 | 0 ± 0 |
|  | Kaempferol | 0 ± 0 | 0 ± 0 | 0.03 ± 0 | 0.17 ± 0.1 |
|  | Morin | 0 ± 0 | 0 ± 0 | 0 ± 0 | 0 ± 0 |
|  | Quercetin | 0 ± 0 | 0.01 ± 0 | 0.26 ± 0.04 | 0.23 ± 0.02 |
|  | Myricetin | 0 ± 0 | 0 ± 0 | 0.14 ± 0.01 | 0.15 ± 0 |
|  | Quercetin-3-Glucoside | 0 ± 0 | 0 ± 0 | 0.01 ± 0 | 0 ± 0 |
|  | Taxifolin | 0 ± 0 | 0 ± 0 | 0.04 ± 0.01 | 0.06 ± 0 |
|  | Genistein | 0 ± 0 | 0 ± 0 | 0 ± 0 | 0 ± 0 |
|  | Scopoletin | 0 ± 0 | 0 ± 0 | 0 ± 0 | 0 ± 0 |
|  | Umbelliferone | 0 ± 0 | 0 ± 0 | 0 ± 0 | 0 ± 0 |
|  | 7,8-dihydroxy-6-methyl coumarin | 0 ± 0 | 0 ± 0 | 0 ± 0 | 0 ± 0 |
|  | Neohesperidin | 0 ± 0 | 0 ± 0 | 0 ± 0 | 0 ± 0 |
|  | Hesperidin | 0 ± 0 | 0 ± 0 | 0.02 ± 0 | 0 ± 0 |
|  | Quercitrin | 0 ± 0 | 0 ± 0 | 0.03 ± 0 | 0 ± 0 |
|  | Biochanin A | 0 ± 0 | 0 ± 0 | 0 ± 0 | 0 ± 0 |
|  | Poncirin | 0 ± 0 | 0 ± 0 | 0 ± 0 | 0 ± 0 |
|  | Didymin | 0 ± 0 | 0 ± 0 | 0 ± 0 | 0 ± 0 |
|  | Phloridzin | 0 ± 0 | 0 ± 0 | 0 ± 0 | 0 ± 0 |
|  | Daidzein | 0 ± 0 | 0 ± 0 | 0 ± 0 | 0 ± 0 |
|  | Galangin | 0 ± 0 | 0 ± 0 | 0 ± 0 | 0 ± 0 |
|  | Luteolin | 0.01 ± 0 | 0.02 ± 0 | 0.03 ± 0 | 0.03 ± 0 |
|  | Equol | 0 ± 0 | 0 ± 0 | 0 ± 0 | 0 ± 0 |
|  | Fisetin | 0 ± 0 | 0 ± 0 | 0 ± 0 | 0 ± 0 |
|  | Luteolinidin | 0.02 ± 0.01 | 0.02 ± 0 | 0.03 ± 0.01 | 0.09 ± 0.03 |
|  | Neoeriocitrin | 0 ± 0 | 0 ± 0 | 0 ± 0 | 0 ± 0 |
|  | Isorhamnetin | 0.01 ± 0 | 0.04 ± 0.02 | 0.04 ± 0 | 0.08 ± 0.03 |
|  | Formononetin | 0 ± 0 | 0 ± 0 | 0 ± 0 | 0 ± 0 |
|  | Apigenin | 0.01 ± 0 | 0.01 ± 0 | 0.01 ± 0 | 0.02 ± 0 |
|  | Gossypin | 0 ± 0 | 0 ± 0 | 0 ± 0 | 0 ± 0 |
|  | Rutin | 0 ± 0 | 0 ± 0 | 0.03 ± 0 | 0.03 ± 0.02 |
|  | Vitexin | 0 ± 0 | 0 ± 0 | 0.05 ± 0 | 0.03 ± 0.01 |
|  | Hyperoside | 0 ± 0 | 0 ± 0 | 0.01 ± 0 | 0 ± 0 |
|  | Glycitein | 0 ± 0 | 0 ± 0 | 0 ± 0 | 0 ± 0 |
| **Phenolic dimers and lignans** | Hydrogenated Ferulic Dimer H5-5 | 0 ± 0 | 0 ± 0 | 0 ± 0 | 0 ± 0 |
|  | Secoisolariciresinol | 0 ± 0 | 0 ± 0 | 0 ± 0 | 0 ± 0 |
|  | Matairesinol | 0 ± 0 | 0 ± 0 | 0 ± 0 | 0 ± 0 |
|  | Enterodiol | 0 ± 0 | 0 ± 0 | 0 ± 0 | 0 ± 0 |
|  | Enterlactone | 0 ± 0 | 0 ± 0 | 0 ± 0 | 0 ± 0 |
|  | Syringaresinol | 0 ± 0 | 0 ± 0 | 0 ± 0 | 0 ± 0 |
|  | Hydroxytyrosol | 0 ± 0 | 0 ± 0 | 0.01 ± 0 | 0 ± 0 |
|  | Pinoresinol | 0 ± 0 | 0 ± 0 | 0 ± 0 | 0 ± 0 |
|  | Hydroxymatairesinol | 0 ± 0 | 0 ± 0 | 0 ± 0 | 0 ± 0 |
|  | Kynurenic acid | 0.01 ± 0 | 0.01 ± 0 | 0.01 ± 0 | 0.02 ± 0 |
|  | Caffeine | 0 ± 0 | 0.01 ± 0 | 0 ± 0 | 0.01 ± 0 |
|  | Dopamine | 0 ± 0 | 0.01 ± 0 | 0.01 ± 0 | 0.01 ± 0 |
|  | Serotonin | 0 ± 0 | 0 ± 0 | 0 ± 0 | 0.01 ± 0 |
|  | Lariciresinol | 0 ± 0 | 0 ± 0 | 0 ± 0 | 0 ± 0 |

**Supplementary Table 5** Postprandial plasma concentration following the acute consumption (over 4 h) of the control and bean hull bread rolls.

|  |  | **Control** | | | | | |  | **Broad Bean Bread** | | | | | |
| --- | --- | --- | --- | --- | --- | --- | --- | --- | --- | --- | --- | --- | --- | --- |
|  |  | **0** | **30** | **60** | **120** | **180** | **240** |  | **0** | **30** | **60** | **120** | **180** | **240** |
| **Acetophenones** | 3,4,5-trimethoxyacetophenone | 0 ± 0 | 0 ± 0 | 0 ± 0 | 0 ± 0 | 0 ± 0 | 0 ± 0 |  | 0 ± 0 | 0 ± 0 | 0 ± 0 | 0 ± 0 | 0 ± 0 | 0 ± 0 |
|  | 3,4-dimethoxyacetophenone | 0 ± 0 | 0 ± 0 | 0 ± 0 | 0 ± 0 | 0 ± 0 | 0 ± 0 |  | 0 ± 0 | 0 ± 0 | 0 ± 0 | 0 ± 0 | 0 ± 0 | 0 ± 0 |
|  | 4-hydroxy-3,5-dimethoxyacetophenone | 0 ± 0 | 0 ± 0 | 0 ± 0 | 0 ± 0 | 0 ± 0 | 0 ± 0 |  | 0 ± 0 | 0 ± 0 | 0 ± 0 | 0 ± 0 | 0 ± 0 | 0 ± 0 |
|  | 4-hydroxy-3-methoxyacetophenone | 5.02 ± 0.91 | 5.69 ± 1.99 | 4.99 ± 1.53 | 3.9 ± 3.14 | 5.6 ± 1.47 | 4.97 ± 2.22 |  | 4.37 ± 2.81 | 6.51 ± 3.68 | 6.41 ± 2.81 | 5.86 ± 2.1 | 6.52 ± 2.99 | 4.52 ± 1.66 |
|  | 4-hydroxyacetophenone | 10.64 ± 3.68 | 10.88 ± 3.43 | 11.86 ± 3.29 | 10.31 ± 2.61 | 10.58 ± 2.36 | 10.47 ± 3.05 |  | 11.1 ± 4.93 | 11.4 ± 4.06 | 11.45 ± 3.61 | 11.3 ± 4.78 | 10.79 ± 3.95 | 11.11 ± 2.68 |
| **Benzaldehydes** | 3,4,5-trihydroxybenzaldehyde | 0 ± 0 | 0 ± 0 | 0 ± 0 | 0 ± 0 | 0 ± 0 | 0 ± 0 |  | 0 ± 0 | 0 ± 0 | 0 ± 0 | 0 ± 0 | 0 ± 0 | 0 ± 0 |
|  | 3,4,5-trimethoxybenzaldehyde | 0 ± 0 | 0 ± 0 | 0 ± 0 | 0 ± 0 | 0 ± 0 | 0 ± 0 |  | 0 ± 0 | 0 ± 0 | 0 ± 0 | 0 ± 0 | 0 ± 0 | 0 ± 0 |
|  | protocatachaldehyde | 49.78 ± 8.28 | 51.69 ± 5.67 | 52.02 ± 3.54 | 50.18 ± 7.51 | 49.64 ± 5.68 | 51.4 ± 8.07 |  | 46.96 ± 6.52 | 51.11 ± 5.53 | 51.13 ± 4.36 | **52.24 ± 4.79** | 48.28 ± 5.27 | 52.77 ± 7.7 |
|  | 3,4-dimethoxybenzaldehyde | 0 ± 0 | 0 ± 0 | 0 ± 0 | 0 ± 0 | 0 ± 0 | 0 ± 0 |  | 0 ± 0 | 0 ± 0 | 0 ± 0 | 0 ± 0 | 0 ± 0 | 0 ± 0 |
|  | syringin | 0 ± 0 | 0 ± 0 | 0 ± 0 | 0 ± 0 | 0 ± 0 | 0 ± 0 |  | 0 ± 0 | 0 ± 0 | 0 ± 0 | 0 ± 0 | 0 ± 0 | 0 ± 0 |
|  | vanillin | 0 ± 0 | 0 ± 0 | 0 ± 0 | 0 ± 0 | 0 ± 0 | 0 ± 0 |  | 0 ± 0 | 0 ± 0 | 0 ± 0 | 0 ± 0 | 0 ± 0 | 0 ± 0 |
|  | 3-methoxybenzaldehyde | 0 ± 0 | 0 ± 0 | 0 ± 0 | 0 ± 0 | 0 ± 0 | 0 ± 0 |  | 0 ± 0 | 0 ± 0 | 0 ± 0 | 0 ± 0 | 0 ± 0 | 0 ± 0 |
|  | p-hydroxybenzaldehyde | 16.95 ± 5.63 | 16.42 ± 5.91 | 16.22 ± 4.75 | 14.88 ± 5.21 | 15.57 ± 5.94 | 15.65 ± 4.99 |  | 14.98 ± 6.68 | 15.7 ± 5.1 | 16.61 ± 5.53 | 16.7 ± 4.93 | 14.98 ± 5.97 | 16.66 ± 7.66 |
|  | isovanillin | 0 ± 0 | 0 ± 0 | 0 ± 0 | 0 ± 0 | 0 ± 0 | 0 ± 0 |  | 0 ± 0 | 0 ± 0 | 0 ± 0 | 0 ± 0 | 0 ± 0 | 0 ± 0 |
| **Benzenes** | 1,2,3-trihydroxybenzene | 0 ± 0 | 0 ± 0 | 0 ± 0 | 0 ± 0 | 0 ± 0 | 0 ± 0 |  | 0 ± 0 | 0 ± 0 | 0 ± 0 | 0 ± 0 | 0 ± 0 | 0 ± 0 |
|  | 1,2-hydroxybenzene | 0 ± 0 | 0 ± 0 | 0 ± 0 | 0 ± 0 | 1.37 ± 4.1 | 0 ± 0 |  | 1.02 ± 3.07 | 0 ± 0 | 0 ± 0 | 0.73 ± 2.18 | 0 ± 0 | 0 ± 0 |
|  | 1,3-hydroxybenzene | 3.56 ± 7.34 | 0.73 ± 2.19 | 0 ± 0 | 0 ± 0 | 1.19 ± 3.57 | 1.96 ± 5.87 |  | 6.73 ± 13.6 | 0 ± 0 | 0 ± 0 | 4.4 ± 9.17 | 0 ± 0 | 3.93 ± 7.14 |
|  | phenol | 0 ± 0 | 0 ± 0 | 0 ± 0 | 0 ± 0 | 0 ± 0 | 0 ± 0 |  | 0 ± 0 | 0 ± 0 | 0 ± 0 | 0 ± 0 | 0 ± 0 | 0 ± 0 |
| **Benzoic Acids** | 2,3-dihydroxybenzoic acid | 0 ± 0 | 0 ± 0 | 0 ± 0 | 0 ± 0 | 0 ± 0 | 0 ± 0 |  | 0 ± 0 | 0 ± 0 | 0 ± 0 | 0 ± 0 | 0 ± 0 | 0 ± 0 |
|  | 2,4-dihydroxybenzoic acid | 0 ± 0 | 0 ± 0 | 0 ± 0 | 0 ± 0 | 0 ± 0 | 0 ± 0 |  | 0 ± 0 | 0 ± 0 | 0 ± 0 | 0 ± 0 | 0 ± 0 | 0 ± 0 |
|  | 2,5-dihydroxybenzoic acid | 0 ± 0 | 0 ± 0 | 0 ± 0 | 0 ± 0 | 0 ± 0 | 0 ± 0 |  | 0 ± 0 | 0 ± 0 | 0 ± 0 | 0 ± 0 | 0 ± 0 | 0 ± 0 |
|  | 2,6-dihydroxybenzoic acid | 18.13 ± 14.96 | 18.64 ± 14.99 | 18.09 ± 14.94 | 15.5 ± 15.75 | 16.64 ± 13.97 | 14.13 ± 14.54 |  | 13.82 ± 10.63 | 14.87 ± 11.36 | 15.55 ± 12.05 | 12.33 ± 11.86 | 13.74 ± 11.65 | 9.85 ± 12.47 |
|  | salicylic acid | 29.06 ± 17.88 | **32.04 ± 20.13** | 32.8 ± 21.17 | 29.73 ± 20.02 | 27.91 ± 18.72 | 28.85 ± 19.99 |  | 29.97 ± 20.12 | 32.92 ± 23.61 | **34.35 ± 24.76** | 31.1 ± 23.23 | 26.27 ± 23.73 | 27.12 ± 25.49 |
|  | o-anisic acid | 0 ± 0 | 0 ± 0 | 0 ± 0 | 0 ± 0 | 0 ± 0 | 0 ± 0 |  | 0 ± 0 | 0 ± 0 | 0 ± 0 | 0 ± 0 | 0 ± 0 | 0 ± 0 |
|  | gallic acid | 0 ± 0 | 0 ± 0 | 0 ± 0 | 0 ± 0 | 0 ± 0 | 0 ± 0 |  | 0 ± 0 | 0 ± 0 | 0 ± 0 | 0 ± 0 | 0 ± 0 | 0 ± 0 |
|  | 3,4-dimethoxybenzoic acid | 11.64 ± 2.45 | 11.76 ± 1.57 | 12.52 ± 2.36 | 12.3 ± 1.68 | 11.41 ± 2.51 | 12.15 ± 2.74 |  | 11.32 ± 2.22 | 12.03 ± 1.94 | **13.21 ± 2.21** | 12.2 ± 1.34 | 11.06 ± 1.93 | 10.97 ± 2.32 |
|  | 3,5-dihydroxybenzoic acid | 0 ± 0 | 0 ± 0 | 0 ± 0 | 0 ± 0 | 0 ± 0 | 0 ± 0 |  | 0 ± 0 | 0 ± 0 | 0 ± 0 | 0 ± 0 | 0 ± 0 | 0 ± 0 |
|  | syringic acid | 110.49 ± 12.41 | 113.71 ± 8.77 | 112.91 ± 15.11 | 108.36 ± 10.82 | 106.31 ± 15.16 | 110.2 ± 9.95 |  | 108.98 ± 14.77 | 111.87 ± 10.35 | 118.73 ± 9.99 | 110.82 ± 13.07 | 108.85 ± 11.69 | 108.6 ± 17.39 |
|  | m-hydroxybenzoic acid | 0 ± 0 | 0 ± 0 | 0 ± 0 | 0 ± 0 | 0 ± 0 | 0 ± 0 |  | 0 ± 0 | 0 ± 0 | 0 ± 0 | 0 ± 0 | 0 ± 0 | 0 ± 0 |
|  | vanillic acid | 1270.22 ± 91.98 | 1274.22 ± 55.97 | 1304.22 ± 83.96 | 1254.44 ± 76.75 | 1263.78 ± 136.07 | 1308.89 ± 75.39 |  | 1216.22 ± 112.66 | 1284.67 ± 75.76 | 1327.33 ± 68.75 | 1305.11 ± 99.05 | 1247.75 ± 133.29 | 1287.43 ± 129.39 |
|  | m-anisic acid | 0 ± 0 | 0 ± 0 | 0 ± 0 | 0 ± 0 | 0 ± 0 | 0 ± 0 |  | 0 ± 0 | 0 ± 0 | 0 ± 0 | 0 ± 0 | 0 ± 0 | 0 ± 0 |
|  | p-anisic acid | 0 ± 0 | 0 ± 0 | 0 ± 0 | 0 ± 0 | 0 ± 0 | 0 ± 0 |  | 0 ± 0 | 0 ± 0 | 0 ± 0 | 0 ± 0 | 0 ± 0 | 0 ± 0 |
|  | benzoic acid | 1308.22 ± 95.03 | 1306.89 ± 162.03 | 1290 ± 171.29 | 1306.67 ± 141.62 | 1332.67 ± 136.59 | 1371.11 ± 149.38 |  | 1283.56 ± 136.44 | **1333.11 ± 113.68** | 1309.78 ± 140.46 | 1319.11 ± 147.19 | 1283.5 ± 164.89 | 1288 ± 84.6 |
|  | p-hydroxybenzoic acid | 1856.89 ± 256.75 | 1883.11 ± 190.08 | 1889.56 ± 218.57 | 1826.89 ± 165.65 | 1836.22 ± 221.21 | 1882.67 ± 135.47 |  | 1720.67 ± 162.46 | **1820.00 ± 106.16** | **1859.11 ± 62.14** | 1826.89 ± 126.53 | 1779.5 ± 181.49 | 1848 ± 218.72 |
|  | protocatechuic acid | 2826.67 ± 272.4 | 2900 ± 152.32 | 2855.56 ± 158.68 | 2775.56 ± 239.75 | 2811.11 ± 222.06 | 2837.78 ± 134.7 |  | 2664.44 ± 312.21 | **2880 ± 200.75** | 2895.56 ± 175.44 | **2948.89 ± 155.28** | 2837.5 ± 251.72 | 2934.29 ± 351.51 |
| **Cinnamic Acids** | o-coumaric acid | 0 ± 0 | 0 ± 0 | 0 ± 0 | 0 ± 0 | 0 ± 0 | 0 ± 0 |  | 0 ± 0 | 0 ± 0 | 0 ± 0 | 0 ± 0 | 0 ± 0 | 0 ± 0 |
|  | 3,4,5-trimethoxycinnamic acid | 0 ± 0 | 0 ± 0 | 0 ± 0 | 0 ± 0 | 0 ± 0 | 0 ± 0 |  | 0 ± 0 | 0 ± 0 | 0 ± 0 | 0 ± 0 | 0 ± 0 | 0 ± 0 |
|  | caffeic acid | 31.56 ± 5.62 | **34.38 ± 3.26** | 33.22 ± 4.07 | 32.52 ± 9.61 | 30.87 ± 5.44 | 31.6 ± 4.8 |  | 30.15 ± 8.81 | 35.4 ± 5.05 | 34.84 ± 6.82 | 34.93 ± 4.39 | 32.93 ± 3.72 | 31.66 ± 5.92 |
|  | 3,4-dimethoxycinnamic acid | 24.84 ± 4.79 | 24.83 ± 5.26 | 24.38 ± 4.38 | 25.31 ± 2.62 | 24.2 ± 3.2 | 24.08 ± 3.43 |  | 26.24 ± 6.99 | 26.7 ± 6.78 | 26.69 ± 4.28 | 26.29 ± 6.2 | 24.44 ± 6.82 | 26.08 ± 4.59 |
|  | m-coumaric acid | 0 ± 0 | 0 ± 0 | 0 ± 0 | 0 ± 0 | 0 ± 0 | 0 ± 0 |  | 0 ± 0 | 0 ± 0 | 0 ± 0 | 0 ± 0 | 0 ± 0 | 0 ± 0 |
|  | ferulic acid | 28.8 ± 4.37 | **32.18 ± 4.06** | **33.07 ± 4.59** | **32.67 ± 3.97** | **32.8 ± 5.91** | 31.33 ± 3.21 |  | 26.49 ± 4.11 | **31.27 ± 2.22** | **33.47 ± 4.63** | **33.71 ± 3.33** | **32.03 ± 4.68** | 29.55 ± 7.01 |
|  | 3-methoxycinnamic acid | 0 ± 0 | 0 ± 0 | 0 ± 0 | 0 ± 0 | 0 ± 0 | 0 ± 0 |  | 0 ± 0 | 0 ± 0 | 0 ± 0 | 0 ± 0 | 0 ± 0 | 0 ± 0 |
|  | p-coumaric acid | 0 ± 0 | 0 ± 0 | 0 ± 0 | 0 ± 0 | 0 ± 0 | 0 ± 0 |  | 0 ± 0 | 0 ± 0 | 0 ± 0 | 0 ± 0 | 0 ± 0 | 0 ± 0 |
|  | 4-methoxycinnamic acid | 0 ± 0 | 0 ± 0 | 0 ± 0 | 0 ± 0 | 0 ± 0 | 0 ± 0 |  | 0 ± 0 | 0 ± 0 | 0 ± 0 | 0 ± 0 | 0 ± 0 | 0 ± 0 |
|  | cinnamic acid | 0 ± 0 | 0 ± 0 | 0 ± 0 | 0 ± 0 | 0 ± 0 | 0 ± 0 |  | 1.04 ± 3.11 | 0 ± 0 | 0 ± 0 | 0 ± 0 | 0 ± 0 | 0 ± 0 |
|  | sinapic acid | 19.94 ± 4.59 | 12.61 ± 9.82 | 19.99 ± 3.67 | 20.8 ± 2.79 | 15.31 ± 9.19 | 19.07 ± 7.57 |  | 17.9 ± 2.59 | 19.4 ± 8.44 | 13.32 ± 10.17 | 11.02 ± 10.66 | 19.37 ± 4.1 | 16.09 ± 7.65 |
| **Flavanoids/Coumarins/Isoflavonoids** | 7,8-dihydroxy-6-methyl coumarin | 0.46 ± 1.37 | 0 ± 0 | 0 ± 0 | 0 ± 0 | 0 ± 0 | 0.46 ± 1.38 |  | 0 ± 0 | 0.52 ± 1.55 | 0 ± 0 | 0.5 ± 1.49 | 0 ± 0 | 0 ± 0 |
|  | 8-methylpsoralen | 2.68 ± 0.65 | 2.7 ± 0.77 | 2.76 ± 0.78 | 2.71 ± 0.51 | 2.68 ± 0.64 | 2.6 ± 0.46 |  | 2.66 ± 0.48 | 2.74 ± 0.72 | 2.85 ± 0.8 | 3.08 ± 0.95 | 2.61 ± 0.55 | 2.79 ± 0.57 |
|  | Apigenin | 1329.33 ± 53.87 | 1404.89 ± 146.76 | 1419.33 ± 134.86 | 1398.67 ± 99.22 | 1383.78 ± 224.11 | 1434.89 ± 168.98 |  | 1273.33 ± 226.17 | **1386.67 ± 210.26** | 1409.33 ± 157.59 | 1426 ± 100.55 | 1374.5 ± 136.48 | 1431.14 ± 162.91 |
|  | Bergapten | 0 ± 0 | 0 ± 0 | 0 ± 0 | 0 ± 0 | 0 ± 0 | 0 ± 0 |  | 0 ± 0 | 0 ± 0 | 0 ± 0 | 0 ± 0 | 0 ± 0 | 0 ± 0 |
|  | Biochanin A | 94.33 ± 3.51 | 95.36 ± 8.25 | 96.49 ± 5.63 | 95.76 ± 6.93 | 95.31 ± 4.03 | 97 ± 4.92 |  | 92.36 ± 11.93 | 96.18 ± 8.79 | 94.89 ± 5.95 | 95.07 ± 5.47 | 96.28 ± 5.76 | 91.66 ± 8.5 |
|  | Catechin | 0 ± 0 | 0 ± 0 | 0 ± 0 | 0 ± 0 | 0 ± 0 | 0 ± 0 |  | 0 ± 0 | 0 ± 0 | 0 ± 0 | 0 ± 0 | 0 ± 0 | 0 ± 0 |
|  | Coumarin | 0 ± 0 | 0 ± 0 | 0 ± 0 | 0 ± 0 | 0 ± 0 | 0 ± 0 |  | 0 ± 0 | 0 ± 0 | 0 ± 0 | 0 ± 0 | 0 ± 0 | 0 ± 0 |
|  | Coumesterol | 0 ± 0 | 0 ± 0 | 0 ± 0 | 0 ± 0 | 0 ± 0 | 0 ± 0 |  | 0 ± 0 | 0 ± 0 | 0 ± 0 | 0 ± 0 | 0 ± 0 | 0 ± 0 |
|  | Daidzein | 19.52 ± 1.83 | 19.49 ± 2.43 | 19.49 ± 2.55 | 19.37 ± 1.66 | 18.87 ± 1.5 | 19.57 ± 1.7 |  | 17.72 ± 2.32 | 18.52 ± 1.95 | 20.01 ± 2.31 | 19.5 ± 1.38 | 19.91 ± 3.73 | 20.15 ± 2.29 |
|  | Didymin | 0 ± 0 | 0 ± 0 | 0 ± 0 | 0 ± 0 | 0 ± 0 | 0 ± 0 |  | 0 ± 0 | 0 ± 0 | 0 ± 0 | 0 ± 0 | 0 ± 0 | 0 ± 0 |
|  | Epicatechin | 0 ± 0 | 0 ± 0 | 0 ± 0 | 0 ± 0 | 0 ± 0 | 0 ± 0 |  | 0 ± 0 | 0 ± 0 | 0 ± 0 | 0 ± 0 | 0 ± 0 | 0 ± 0 |
|  | Epigallocatechin | 0 ± 0 | 0 ± 0 | 0 ± 0 | 0 ± 0 | 0 ± 0 | 0 ± 0 |  | 0 ± 0 | 0 ± 0 | 0 ± 0 | 0 ± 0 | 0 ± 0 | 0 ± 0 |
|  | Epigallocatechin Gallate | 0 ± 0 | 0 ± 0 | 0 ± 0 | 0 ± 0 | 0 ± 0 | 0 ± 0 |  | 0 ± 0 | 0 ± 0 | 0 ± 0 | 0 ± 0 | 0 ± 0 | 0 ± 0 |
|  | Equol | 0 ± 0 | 0 ± 0 | 0 ± 0 | 0 ± 0 | 0 ± 0 | 0 ± 0 |  | 0 ± 0 | 0 ± 0 | 0 ± 0 | 0 ± 0 | 0 ± 0 | 0 ± 0 |
|  | Eriocitrin | 0 ± 0 | 0 ± 0 | 0 ± 0 | 0 ± 0 | 0 ± 0 | 0 ± 0 |  | 0 ± 0 | 0 ± 0 | 0 ± 0 | 0 ± 0 | 0 ± 0 | 0 ± 0 |
|  | Fisetin | 0 ± 0 | 0 ± 0 | 0 ± 0 | 0 ± 0 | 0 ± 0 | 0 ± 0 |  | 0 ± 0 | 0 ± 0 | 0 ± 0 | 0 ± 0 | 0 ± 0 | 0 ± 0 |
|  | Formononetin | 179.04 ± 8.57 | 179.56 ± 13.8 | 180.89 ± 9.92 | 181.24 ± 9.24 | 172 ± 8.48 | 175.58 ± 7.55 |  | 165.24 ± 19.38 | **177.98 ± 16.19** | 177.6 ± 13.3 | 179.64 ± 9.64 | 174.58 ± 9.78 | 176.69 ± 13.66 |
|  | Galangin | 0 ± 0 | 0 ± 0 | 0 ± 0 | 0 ± 0 | 0 ± 0 | 0 ± 0 |  | 0 ± 0 | 0 ± 0 | 0 ± 0 | 0 ± 0 | 0 ± 0 | 0 ± 0 |
|  | Gallocatechin | 0 ± 0 | 0 ± 0 | 0 ± 0 | 0 ± 0 | 0 ± 0 | 0 ± 0 |  | 0 ± 0 | 0 ± 0 | 0 ± 0 | 0 ± 0 | 0 ± 0 | 0 ± 0 |
|  | Genistein | 150.51 ± 4.02 | 152.18 ± 13.66 | 155.8 ± 12.37 | 152.56 ± 11.81 | 148.18 ± 8.03 | 149.27 ± 10.24 |  | 149.87 ± 18.14 | 152.89 ± 16.36 | 151.29 ± 6.91 | 157.44 ± 7.38 | 155.53 ± 8.94 | 156.06 ± 11.49 |
|  | Gossypin | 0 ± 0 | 0 ± 0 | 0 ± 0 | 0 ± 0 | 0 ± 0 | 0 ± 0 |  | 0 ± 0 | 0 ± 0 | 0 ± 0 | 0 ± 0 | 0 ± 0 | 0 ± 0 |
|  | Hesperidin | 0 ± 0 | 0 ± 0 | 0 ± 0 | 0 ± 0 | 0 ± 0 | 0 ± 0 |  | 0 ± 0 | 0 ± 0 | 0 ± 0 | 0 ± 0 | 0 ± 0 | 0 ± 0 |
|  | Hesperitin | 69.64 ± 4.14 | 73.51 ± 6.16 | 72.04 ± 3.91 | 70.29 ± 3.29 | 68.76 ± 6.64 | 69.98 ± 4.04 |  | 68.44 ± 8.27 | 71.36 ± 7.27 | 73.09 ± 3.71 | 72.51 ± 5.81 | 72.6 ± 4.83 | 71.97 ± 4.32 |
|  | Imperatorin | 2.54 ± 2.02 | 2.34 ± 2.3 | 2.3 ± 2.25 | 1.8 ± 2.16 | 1.88 ± 2.24 | 2.96 ± 1.8 |  | 1.7 ± 2.06 | 3.07 ± 1.78 | 3.1 ± 1.79 | 2.73 ± 2.12 | 2.04 ± 2.2 | 3.43 ± 1.54 |
|  | Isoliquiritigenin | 0 ± 0 | 0 ± 0 | 0 ± 0 | 0 ± 0 | 0 ± 0 | 0 ± 0 |  | 0 ± 0 | 0 ± 0 | 0 ± 0 | 0 ± 0 | 0 ± 0 | 0 ± 0 |
|  | Isorhamnetin | 1099.11 ± 48.98 | 1135.56 ± 68.76 | 1132.67 ± 81.24 | 1126.89 ± 84.06 | 1106.89 ± 78.86 | 1111.33 ± 65.64 |  | 1091.78 ± 126.52 | 1112.89 ± 100.88 | 1100.22 ± 80.75 | 1138.67 ± 74.07 | 1124.75 ± 95.56 | 1118.86 ± 66.52 |
|  | Kaempferol | 536.44 ± 43.49 | 540.67 ± 59.76 | 569.11 ± 37.78 | 558.44 ± 65.37 | 517.11 ± 42.19 | 536.89 ± 40.42 |  | 508.89 ± 80.03 | 536.44 ± 70.18 | 522.67 ± 26.31 | 515.78 ± 44.37 | 526.25 ± 57.16 | 545.14 ± 44.5 |
|  | Luteolin | 3882.22 ± 170.42 | 3948.89 ± 221.38 | 3975.56 ± 278.53 | 3977.78 ± 201.36 | 3946.67 ± 245.97 | 3900 ± 375.37 |  | 3722.22 ± 399.56 | **3955.56 ± 441** | 3895.56 ± 313.33 | 3993.33 ± 164.32 | 3962.5 ± 192.26 | 3922.86 ± 154.24 |
|  | Luteolinidin | 11.31 ± 4.45 | 11.37 ± 4.46 | 9.72 ± 5.68 | 11.88 ± 1.26 | 12.64 ± 1.59 | 10.8 ± 4.34 |  | 10.4 ± 4 | 8.02 ± 6.15 | 11.05 ± 4.43 | 12.14 ± 1.23 | 9.48 ± 6.16 | 10.47 ± 4.82 |
|  | Morin | 72.04 ± 4.24 | 71.62 ± 8.18 | 77.31 ± 6.75 | 75.36 ± 4.58 | 75.87 ± 8.28 | 74.27 ± 5.48 |  | 70.98 ± 6.6 | **76.18 ± 8.46** | **76.73 ± 6.36** | 76.84 ± 7.74 | 72.7 ± 7.04 | 77.74 ± 6.26 |
|  | Myricetin | 0 ± 0 | 0 ± 0 | 0 ± 0 | 0 ± 0 | 0 ± 0 | 0 ± 0 |  | 0 ± 0 | 0 ± 0 | 0 ± 0 | 0 ± 0 | 0 ± 0 | 0 ± 0 |
|  | Naringenin | 52.53 ± 2.86 | 51.38 ± 5.61 | 52.87 ± 2.76 | 50.84 ± 3.17 | 52.6 ± 4.37 | 49.93 ± 4.38 |  | 50.93 ± 7.94 | 53 ± 5.57 | 51.16 ± 4.45 | 51.6 ± 4.98 | 51.05 ± 4.65 | 51.66 ± 4.56 |
|  | Naringin | 0 ± 0 | 0 ± 0 | 0 ± 0 | 0 ± 0 | 0 ± 0 | 0 ± 0 |  | 0 ± 0 | 0 ± 0 | 0 ± 0 | 0 ± 0 | 0 ± 0 | 0 ± 0 |
|  | Neoeriocitrin | 0 ± 0 | 0 ± 0 | 0 ± 0 | 0 ± 0 | 0 ± 0 | 0 ± 0 |  | 0 ± 0 | 0 ± 0 | 0 ± 0 | 0 ± 0 | 0 ± 0 | 0 ± 0 |
|  | Neohesperidin | 0 ± 0 | 0 ± 0 | 0 ± 0 | 0 ± 0 | 0 ± 0 | 0 ± 0 |  | 0 ± 0 | 0 ± 0 | 0 ± 0 | 0 ± 0 | 0 ± 0 | 0 ± 0 |
|  | Phloretin | 0 ± 0 | 0 ± 0 | 0 ± 0 | 0 ± 0 | 0 ± 0 | 0 ± 0 |  | 0 ± 0 | 0 ± 0 | 0 ± 0 | 0 ± 0 | 0 ± 0 | 0 ± 0 |
|  | Phloridzin | 0 ± 0 | 0 ± 0 | 0 ± 0 | 0 ± 0 | 0 ± 0 | 0 ± 0 |  | 0 ± 0 | 0 ± 0 | 0 ± 0 | 0 ± 0 | 0 ± 0 | 0 ± 0 |
|  | Poncirin | 0 ± 0 | 0 ± 0 | 0 ± 0 | 0 ± 0 | 0 ± 0 | 0 ± 0 |  | 0 ± 0 | 0 ± 0 | 0 ± 0 | 0 ± 0 | 0 ± 0 | 0 ± 0 |
|  | Psoralen | 4.79 ± 1.77 | 4.9 ± 1.42 | 4.84 ± 1.61 | 4.6 ± 0.82 | 4.9 ± 1.15 | 4.6 ± 0.98 |  | 4.63 ± 0.98 | 4.81 ± 0.82 | 4.77 ± 1.5 | 4.9 ± 1.38 | 4.9 ± 1.11 | 4.99 ± 0.88 |
|  | Quercetin | 633.78 ± 51.78 | 672.67 ± 61.05 | 668.67 ± 59.39 | 644.44 ± 87.05 | 627.56 ± 59.67 | 648.67 ± 63.28 |  | 624.67 ± 108.6 | **687.78 ± 88.05** | 649.78 ± 55.82 | 639.78 ± 75.09 | 639 ± 75.88 | 643.14 ± 60.56 |
|  | Quercetin-3-Glucoside | 0 ± 0 | 0 ± 0 | 0 ± 0 | 0 ± 0 | 0 ± 0 | 0 ± 0 |  | 0 ± 0 | 0 ± 0 | 0 ± 0 | 0 ± 0 | 0 ± 0 | 0 ± 0 |
|  | Quercitrin | 0 ± 0 | 0 ± 0 | 0 ± 0 | 0 ± 0 | 0 ± 0 | 0 ± 0 |  | 0 ± 0 | 0 ± 0 | 0 ± 0 | 0 ± 0 | 0 ± 0 | 0 ± 0 |
|  | Scopoletin | 75.47 ± 5.66 | 77.09 ± 3.91 | 77.49 ± 5.85 | 76.07 ± 5.84 | 75.16 ± 6.91 | 76.04 ± 5.47 |  | 72.36 ± 3.65 | **76.18 ± 7.68** | **77.4 ± 4.32** | 74.6 ± 2.95 | 77.13 ± 9.23 | 75.51 ± 9.09 |
|  | Tangeretin | 0.74 ± 1.47 | 0.31 ± 0.92 | 1.04 ± 1.6 | 0.93 ± 1.4 | 0.97 ± 1.46 | 1.11 ± 1.68 |  | 0.3 ± 0.89 | 0.71 ± 1.42 | 0.37 ± 1.12 | 0.75 ± 1.49 | 0.72 ± 1.34 | 0.4 ± 1.06 |
|  | Taxifolin | 0 ± 0 | 0 ± 0 | 0 ± 0 | 0 ± 0 | 0 ± 0 | 0 ± 0 |  | 0 ± 0 | 0 ± 0 | 0 ± 0 | 0 ± 0 | 0 ± 0 | 0 ± 0 |
|  | Umbelliferone | 28.31 ± 3.06 | 29.78 ± 2.28 | 30.07 ± 2.15 | 28.6 ± 1.97 | 28.31 ± 3.31 | 28.13 ± 1.35 |  | 28.29 ± 2.32 | **29.96 ± 3.75** | 29.6 ± 2.03 | 28.67 ± 1.47 | 28.78 ± 4.4 | 29.03 ± 4.04 |
|  | Hyperoside | 0 ± 0 | 0 ± 0 | 0 ± 0 | 0 ± 0 | 0 ± 0 | 0 ± 0 |  | 0 ± 0 | 0 ± 0 | 0 ± 0 | 0 ± 0 | 0 ± 0 | 0 ± 0 |
|  | Rutin | 0 ± 0 | 0 ± 0 | 0 ± 0 | 0 ± 0 | 0 ± 0 | 0 ± 0 |  | 0 ± 0 | 0 ± 0 | 0 ± 0 | 0 ± 0 | 0 ± 0 | 5.29 ± 13.98 |
|  | Vitexin | 466.67 ± 38.22 | 471.11 ± 42.91 | 465.56 ± 30.31 | 455.78 ± 33.37 | 458.89 ± 36.88 | 442.67 ± 26.04 |  | 449.11 ± 38.73 | 470.67 ± 37.59 | 456 ± 33.53 | 466.67 ± 28.84 | 460.25 ± 47.63 | 468.86 ± 51.17 |
|  | Glycitein | 6.85 ± 0.97 | 6.49 ± 0.79 | 7.08 ± 1.24 | 6.72 ± 0.74 | 6.71 ± 0.39 | 6.53 ± 0.63 |  | 6.22 ± 1.09 | 6.57 ± 0.54 | **7.58 ± 0.62** | **6.97 ± 0.68** | 6.63 ± 0.76 | 7.01 ± 0.86 |
| **Indoles** | indole-3-acetic acid | 561.11 ± 104.43 | **693.78 ± 81.64** | **776.89 ± 117.4** | **821.56 ± 112.78** | **807.11 ± 111.03** | **727.56 ± 108.82** |  | 532.89 ± 105.28 | **642 ± 60.54** | **688.44 ± 68.34** | **731.11 ± 73.89** | **679 ± 140.21** | **646.29 ± 132.42** |
|  | indole-3-carboxylic acid | 39.73 ± 3.83 | 41.44 ± 2.35 | 41.78 ± 3.09 | 41.02 ± 3.42 | 39.84 ± 2.43 | 40.24 ± 2.74 |  | 38.82 ± 4.77 | 41.24 ± 2.33 | 42.07 ± 2.06 | 41.18 ± 2.49 | 38.95 ± 4.36 | 40.23 ± 4.48 |
|  | indole-3-propionic acid | 244.8 ± 89.16 | 253.89 ± 93.86 | 252.29 ± 96.56 | 260.09 ± 111.19 | 252.73 ± 101.87 | 246.31 ± 108.05 |  | 222.2 ± 78.21 | **239.73 ± 79.19** | 259.02 ± 92.19 | 249.18 ± 83.28 | 258.88 ± 53.28 | 270.97 ± 64.81 |
|  | indole-3-carbinol | 0 ± 0 | 0 ± 0 | 0 ± 0 | 0 ± 0 | 0 ± 0 | 0 ± 0 |  | 0 ± 0 | 0 ± 0 | 0 ± 0 | 0 ± 0 | 0 ± 0 | 0 ± 0 |
|  | indole-3-pyruvic acid | 0 ± 0 | 0 ± 0 | 0 ± 0 | 0 ± 0 | 0 ± 0 | 0 ± 0 |  | 0 ± 0 | 0 ± 0 | 0 ± 0 | 0 ± 0 | 0 ± 0 | 0 ± 0 |
|  | indoLe-3-lactic acid | 160.98 ± 47.09 | 158.76 ± 36.69 | 152.89 ± 27.76 | 160.73 ± 27.09 | 154.58 ± 26.45 | 141.53 ± 18.6 |  | 138.49 ± 30.01 | 151.73 ± 22.03 | **167.93 ± 47.65** | 153.78 ± 37.02 | 157.4 ± 19.52 | 160.11 ± 28.16 |
|  | indole-3-methyl | 0 ± 0 | 0 ± 0 | 0 ± 0 | 0 ± 0 | 0 ± 0 | 0 ± 0 |  | 0 ± 0 | 0 ± 0 | 0 ± 0 | 0 ± 0 | 0 ± 0 | 0 ± 0 |
|  | Indole-acrylic acid | 0 ± 0 | 0 ± 0 | 0 ± 0 | 0 ± 0 | 0 ± 0 | 0 ± 0 |  | 0 ± 0 | 0 ± 0 | 0 ± 0 | 0 ± 0 | 0 ± 0 | 0 ± 0 |
|  | 3-Indoleacetonitrile | 0 ± 0 | 0 ± 0 | 0 ± 0 | 0 ± 0 | 0 ± 0 | 0 ± 0 |  | 0 ± 0 | 0 ± 0 | 0 ± 0 | 0 ± 0 | 0 ± 0 | 0 ± 0 |
|  | Indole-3-Carboxaldehyde | 0.17 ± 0.44 | 0.25 ± 0.45 | 0.19 ± 0.35 | 0.3 ± 0.61 | 0.2 ± 0.39 | 0.08 ± 0.24 |  | 0 ± 0 | 0.14 ± 0.42 | 0.37 ± 0.69 | 0.37 ± 0.69 | 0.25 ± 0.7 | 0.37 ± 0.98 |
| **Mandelic Acids** | 3,4-dihydroxymandelic acid | 0 ± 0 | 0 ± 0 | 0 ± 0 | 0 ± 0 | 0 ± 0 | 0 ± 0 |  | 0 ± 0 | 0 ± 0 | 0 ± 0 | 0 ± 0 | 0 ± 0 | 0 ± 0 |
|  | 3-hydroxymandelic acid | 0 ± 0 | 0 ± 0 | 0 ± 0 | 0 ± 0 | 0 ± 0 | 0 ± 0 |  | 0 ± 0 | 0 ± 0 | 0 ± 0 | 0 ± 0 | 0 ± 0 | 0 ± 0 |
|  | 4-hydroxymandelic acid | 426 ± 50.56 | 426 ± 26.23 | 423.56 ± 34.41 | 422.22 ± 39.81 | 430 ± 61.77 | 423.56 ± 30.23 |  | 387.56 ± 49.01 | **418.44 ± 40.27** | 425.56 ± 24.22 | **425.78 ± 43.56** | 415.25 ± 60.08 | 424.86 ± 78.07 |
|  | mandelic acid | 2082.67 ± 176.93 | 2134 ± 108.55 | 2164.89 ± 171.64 | 2144.44 ± 98.88 | 2153.33 ± 251.9 | 2170.89 ± 185.36 |  | 2033.11 ± 242.89 | **2135.56 ± 171.18** | 2172.67 ± 182.49 | 2170.22 ± 153.17 | 1999.5 ± 361.22 | 2147.43 ± 355.71 |
|  | 4-hydroxy-3-methoxymandelic acid | 51.27 ± 6.01 | 48.27 ± 7.87 | 50.11 ± 8.18 | 48.6 ± 6.47 | 47.8 ± 6.94 | 52.27 ± 5.43 |  | 44.16 ± 6.16 | 48.56 ± 5.81 | 49.11 ± 5.13 | 48.98 ± 6.64 | 46.48 ± 8.71 | 50.89 ± 10.45 |
| **Phenolic Dimers and Lignans** | ellagic Acid | 0 ± 0 | 0 ± 0 | 0 ± 0 | 0 ± 0 | 0 ± 0 | 0 ± 0 |  | 0 ± 0 | 0 ± 0 | 0 ± 0 | 0 ± 0 | 0 ± 0 | 0 ± 0 |
|  | ferulic dimer (5-5 linked) | 0 ± 0 | 0 ± 0 | 0 ± 0 | 0 ± 0 | 0 ± 0 | 0 ± 0 |  | 0 ± 0 | 0 ± 0 | 0 ± 0 | 0 ± 0 | 0 ± 0 | 0 ± 0 |
|  | ferulic dimer (8-5 linked) | 21.32 ± 4.03 | 20.48 ± 3.43 | 20.34 ± 4.06 | 20.66 ± 3.37 | 20.22 ± 5.29 | 21.65 ± 4.42 |  | 20.09 ± 3.56 | 21.35 ± 4.74 | 21.29 ± 4.38 | 21.87 ± 3.89 | 21.18 ± 5.41 | 19.96 ± 5.55 |
|  | ferulic dimer (8-8 linked) | 0 ± 0 | 0 ± 0 | 0 ± 0 | 0 ± 0 | 0 ± 0 | 0 ± 0 |  | 0 ± 0 | 0 ± 0 | 0 ± 0 | 0 ± 0 | 0 ± 0 | 0 ± 0 |
|  | Indole | 0 ± 0 | 0 ± 0 | 0 ± 0 | 0 ± 0 | 0 ± 0 | 0 ± 0 |  | 0 ± 0 | 0 ± 0 | 0 ± 0 | 0 ± 0 | 0 ± 0 | 0 ± 0 |
|  | Resveratrol | 0 ± 0 | 0 ± 0 | 0 ± 0 | 0 ± 0 | 0 ± 0 | 0 ± 0 |  | 0 ± 0 | 0 ± 0 | 0 ± 0 | 0 ± 0 | 0 ± 0 | 0 ± 0 |
|  | Syringaresinol | 1283.78 ± 263.99 | 1252.22 ± 257.41 | 1229.11 ± 251.25 | 1211.33 ± 203.55 | 1251.78 ± 366.53 | 1256.67 ± 226.88 |  | 1156 ± 189.47 | 1258.67 ± 241.06 | 1203.78 ± 207.11 | 1238.89 ± 224.26 | 1268 ± 335.37 | 1224.86 ± 267.52 |
|  | Hydroxymatairesinol | 0 ± 0 | 0 ± 0 | 0 ± 0 | 0 ± 0 | 0 ± 0 | 0 ± 0 |  | 0 ± 0 | 0 ± 0 | 0 ± 0 | 0 ± 0 | 0 ± 0 | 0 ± 0 |
|  | Lariciresinol | 0 ± 0 | 0 ± 0 | 0 ± 0 | 0 ± 0 | 0 ± 0 | 0 ± 0 |  | 0 ± 0 | 0 ± 0 | 0 ± 0 | 0 ± 0 | 0 ± 0 | 0 ± 0 |
|  | Matairesinol | 3.85 ± 5.8 | 5.24 ± 6.34 | 4.09 ± 6.27 | 2.37 ± 4.76 | 2.62 ± 5.29 | 2.74 ± 5.46 |  | 3.02 ± 6.01 | 1.36 ± 4.08 | 4.54 ± 5.43 | 4.15 ± 6.4 | 2.56 ± 4.9 | 3.21 ± 5.48 |
|  | Pinoresinol | 357.56 ± 63.37 | 352 ± 36.24 | 352.44 ± 52.91 | 352.44 ± 56.48 | 361.33 ± 72.12 | 362 ± 59.34 |  | 345.56 ± 61.05 | 354.89 ± 56.04 | 346.22 ± 47.13 | 350.22 ± 48.83 | 349 ± 75.87 | 346 ± 77.54 |
|  | Secoisolariciresinol | 50.8 ± 7.82 | 51.64 ± 7.03 | 52.56 ± 8.79 | 52.76 ± 8.98 | 52.58 ± 12.39 | 55.42 ± 9.03 |  | 48.84 ± 9.81 | 50.69 ± 9.45 | 50.33 ± 8.72 | 50.04 ± 8 | 50.3 ± 12.12 | 50.06 ± 12.97 |
|  | Enterodiol | 0.75 ± 2.24 | 0.64 ± 1.91 | 0.54 ± 1.61 | 0.59 ± 1.77 | 0.53 ± 1.59 | 0.48 ± 1.45 |  | 0.6 ± 1.8 | 0 ± 0 | 0.47 ± 1.41 | 0 ± 0 | 0.58 ± 1.63 | 0 ± 0 |
|  | Enterlactone | 9.62 ± 10.49 | 9.02 ± 9.48 | 8.05 ± 8.53 | **6.62 ± 7.71** | **5.66 ± 7.3** | **5.91 ± 7.91** |  | 4.04 ± 5.69 | 4.44 ± 5.97 | 3.69 ± 4.97 | 3.44 ± 4.62 | 3.33 ± 3.71 | 2.87 ± 3.87 |
|  | Hydrogenated Ferulic Dimer H5-6 | 4.58 ± 5.5 | 4.82 ± 4.64 | 4.58 ± 5.54 | 3.37 ± 5.06 | 7.06 ± 5.43 | 6.43 ± 4.96 |  | 4.92 ± 4.82 | 1.35 ± 4.05 | 6.16 ± 4.75 | 5.05 ± 4.82 | 5.94 ± 5.02 | 5.5 ± 5.16 |
| **Phenolics Others** | 4-methylcatechol | 5.84 ± 17.53 | 4.84 ± 14.53 | 4.98 ± 14.93 | 1.34 ± 4.01 | 2.38 ± 7.13 | 2.2 ± 6.61 |  | 0 ± 0 | 0 ± 0 | 0 ± 0 | 0 ± 0 | 0 ± 0 | 0 ± 0 |
|  | quinadilic acid | 6.86 ± 1.89 | 6.5 ± 1.54 | 6.92 ± 1.69 | 6.5 ± 1.38 | 6.62 ± 1.69 | **6.30 ± 1.52** |  | 6.66 ± 2.35 | 6.69 ± 1.82 | 6.86 ± 2.09 | 6.22 ± 1.79 | 6.19 ± 1.67 | 6.19 ± 1.59 |
|  | anthranilic acid | 5.88 ± 5.55 | 4.77 ± 5.58 | 3.99 ± 4.87 | 3.7 ± 4.19 | 4.5 ± 4.13 | **3.48 ± 4.02** |  | 5.28 ± 4.58 | 4.13 ± 4.72 | 4.54 ± 5.16 | 4.03 ± 3.41 | 4.01 ± 2.62 | 2.86 ± 3.68 |
|  | 0-hydroxyhippuric acid | 2.42 ± 2.1 | 2.66 ± 2.24 | 2.54 ± 2.12 | 2.08 ± 2.11 | 1.77 ± 1.97 | 1.72 ± 2.11 |  | 2.01 ± 2.62 | 2.09 ± 2.84 | 2.47 ± 3.06 | 2.42 ± 3.11 | 1.79 ± 3.18 | 2.09 ± 4.13 |
|  | coniferyl alcohol | 0 ± 0 | 0 ± 0 | 0 ± 0 | 0 ± 0 | 0 ± 0 | 0 ± 0 |  | 0 ± 0 | 0 ± 0 | 0 ± 0 | 0 ± 0 | 0 ± 0 | 0 ± 0 |
|  | 4-ethylphenol | 0 ± 0 | 0 ± 0 | 0 ± 0 | 0 ± 0 | 0 ± 0 | 0 ± 0 |  | 0 ± 0 | 0 ± 0 | 0 ± 0 | 0 ± 0 | 0 ± 0 | 0 ± 0 |
|  | ethylferulate | 0 ± 0 | 0 ± 0 | 0 ± 0 | 0 ± 0 | 0 ± 0 | 0 ± 0 |  | 0 ± 0 | 0 ± 0 | 0 ± 0 | 0 ± 0 | 0 ± 0 | 0 ± 0 |
|  | p-cresol | 0 ± 0 | 0 ± 0 | 0 ± 0 | 0 ± 0 | 0 ± 0 | 0 ± 0 |  | 0 ± 0 | 0 ± 0 | 0 ± 0 | 0 ± 0 | 0 ± 0 | 0 ± 0 |
|  | chlorogenic acid | 0 ± 0 | 0 ± 0 | 0 ± 0 | 0 ± 0 | 0 ± 0 | 0 ± 0 |  | 0 ± 0 | 0 ± 0 | 0 ± 0 | 0 ± 0 | 0 ± 0 | 0 ± 0 |
|  | Tyrosol | 0 ± 0 | 0 ± 0 | 0 ± 0 | 0 ± 0 | 0 ± 0 | 0 ± 0 |  | 0 ± 0 | 0 ± 0 | 0 ± 0 | 0 ± 0 | 0 ± 0 | 0 ± 0 |
|  | Hydroxytyrosol | 0 ± 0 | 0 ± 0 | 0 ± 0 | 0 ± 0 | 0 ± 0 | 0 ± 0 |  | 0 ± 0 | 0 ± 0 | 0 ± 0 | 0 ± 0 | 0 ± 0 | 0 ± 0 |
|  |  |  |  |  |  |  |  |  |  |  |  |  |  |  |
|  | Caffeine | 264.14 ± 246.81 | 242.22 ± 233.53 | 223.27 ± 213.64 | **191.98 ± 185.32** | **164.98 ± 155.02** | **135.83 ± 137.25** |  | 219.46 ± 278.4 | 205.44 ± 254.96 | 215.77 ± 281.89 | **174.24 ± 235.41** | **164.81 ± 217.79** | 143.58 ± 181.56 |
|  | Dopamine | 40.71 ± 14.29 | 39.04 ± 9.76 | 39.11 ± 8.86 | 38.24 ± 8.02 | 37.62 ± 7.03 | 39 ± 7.2 |  | 41.49 ± 22.54 | 51.76 ± 40.39 | **46.24 ± 19.13** | 43.13 ± 10.29 | 38.53 ± 9.58 | 38.26 ± 8.42 |
|  | Kynurenic acid | 1189.56 ± 187.33 | 1170.44 ± 128.94 | 1167.33 ± 162.03 | 1172.22 ± 156.67 | 1187.33 ± 159.15 | 1158.44 ± 98.26 |  | 1148.67 ± 153.81 | 1180.22 ± 127.16 | 1187.78 ± 142.03 | 1152.67 ± 89.37 | 1147 ± 161.91 | 1114.86 ± 176.48 |
|  | Melatonin | 0 ± 0 | 0 ± 0 | 0 ± 0 | 0 ± 0 | 0 ± 0 | 0 ± 0 |  | 0 ± 0 | 0 ± 0 | 0 ± 0 | 0 ± 0 | 0 ± 0 | 0 ± 0 |
|  | Niacin | 359.11 ± 61.53 | 352 ± 34.47 | 361.11 ± 31.13 | 354 ± 48.49 | 362.67 ± 67.57 | 359.33 ± 32.37 |  | 323.78 ± 67.09 | **380 ± 30.72** | **372 ± 39.76** | **381.78 ± 30.81** | 347.75 ± 71.78 | 346.57 ± 64.08 |
|  |  |  |  |  |  |  |  |  |  |  |  |  |  |  |
|  |  |  |  |  |  |  |  |  |  |  |  |  |  |  |
| **Phenylacetic acids** | 3,4-dihydroxyphenylacetic acid | 0 ± 0 | 0 ± 0 | 0 ± 0 | 0 ± 0 | 0 ± 0 | 0 ± 0 |  | 0 ± 0 | 0 ± 0 | 0 ± 0 | 0 ± 0 | 0 ± 0 | 0 ± 0 |
|  | 3-hydroxyphenylacetic acid | 9.37 ± 10.51 | 8.33 ± 11.66 | 6.79 ± 10.57 | 6.92 ± 11.64 | 8.75 ± 10.17 | **6.58 ± 9.01** |  | 7 ± 6.26 | 6.88 ± 8.21 | 8.01 ± 6.92 | 6.66 ± 8.24 | 6.28 ± 5.77 | 5.21 ± 5.22 |
|  | 4-hydroxy-3-methoxyphenylacetic acid | 0 ± 0 | 0 ± 0 | 0 ± 0 | 0 ± 0 | 0 ± 0 | 0 ± 0 |  | 0 ± 0 | 0 ± 0 | 0 ± 0 | 0 ± 0 | 0 ± 0 | 0 ± 0 |
|  | 4-hydroxyphenylacetic acid | 663.56 ± 80.04 | 660 ± 44.43 | 686.89 ± 72.53 | 671.33 ± 56.14 | 661.33 ± 83.93 | 697.56 ± 43.8 |  | 636.44 ± 74.25 | 667.11 ± 60.55 | 676.44 ± 43.67 | 675.11 ± 69.33 | 638.75 ± 97.27 | 671.43 ± 120.16 |
|  | 4-methoxyphenylacetic acid | 8.92 ± 1.44 | 8.4 ± 1.76 | 9.45 ± 1.21 | 8.73 ± 4.13 | 8.47 ± 1.73 | 9.9 ± 1.29 |  | 7.75 ± 3 | 9.63 ± 2.2 | 9.16 ± 1.95 | **9.69 ± 1.45** | 9.77 ± 2.12 | 9.87 ± 1.96 |
|  | phenylacetic acid | 7580 ± 800.75 | 7585 ± 368.12 | 7580 ± 498.4 | 7715.56 ± 404.2 | 7644.44 ± 646.59 | 7688.89 ± 330.47 |  | 7148.89 ± 652.47 | **7591.11 ± 435.1** | **7760 ± 309.03** | **7573.33 ± 247.79** | 7440 ± 849.87 | 7762.86 ± 987.15 |
| **Phenyllactic Acids** | 4-hydroxyphenyllactic acid | 239.8 ± 46.23 | 244.56 ± 42.43 | 248.31 ± 56.69 | 254 ± 48.21 | 240 ± 42.53 | 227.4 ± 42.27 |  | 225.49 ± 38.79 | **240.11 ± 41.26** | **256.18 ± 37.42** | 236.56 ± 37.37 | 229.85 ± 42.98 | 224.69 ± 36.12 |
|  | phenyllactic acid | 5308.89 ± 1031.8 | 5157.78 ± 808.24 | 5144.44 ± 977.38 | 5251.11 ± 827.9 | 5242.22 ± 1316.38 | 5324.44 ± 992.56 |  | 4924.44 ± 794.03 | 5108.89 ± 888.26 | 5068.89 ± 870.18 | 5088.89 ± 846.17 | 5015 ± 1174.43 | 4911.43 ± 1154.46 |
| **Phenylpropionic acids** | 2-hydroxyphenylpropionic acid | 8.25 ± 3.32 | 7.28 ± 3.14 | 8.74 ± 3.77 | 7.54 ± 4.86 | 8.39 ± 3.33 | 7.63 ± 3.63 |  | 6.33 ± 3.95 | 6.84 ± 4.27 | 8.16 ± 6.05 | 9.71 ± 2.69 | 8.44 ± 3.96 | 5.53 ± 5.41 |
|  | 3,4-dihydroxyphenylpropionic acid | 35.58 ± 42.46 | 67.27 ± 38.8 | 57.71 ± 43.73 | 56.67 ± 43.39 | 46.38 ± 45.23 | 50.02 ± 47.92 |  | 43.18 ± 41.82 | 39.64 ± 48.18 | 72.09 ± 42.42 | 26.36 ± 39.55 | 29.88 ± 43.02 | 51.57 ± 50.74 |
|  | 3-hydroxyphenylpropionic acid | 15.02 ± 22.71 | 0 ± 0 | 4.29 ± 12.87 | 5.16 ± 15.47 | 0 ± 0 | 3.29 ± 9.87 |  | 9.58 ± 19.72 | 3.11 ± 9.33 | 0 ± 0 | 2.82 ± 8.47 | 0 ± 0 | 0 ± 0 |
|  | 4-hydroxy-3-methoxyphenylpropionic acid | 744.89 ± 68.9 | 764.89 ± 52.8 | 763.11 ± 60.77 | 768.89 ± 60.48 | 754.89 ± 89.14 | 777.56 ± 48.98 |  | 713.11 ± 74.51 | **765.56 ± 47.94** | 778.22 ± 35.21 | 771.78 ± 37.81 | 744.25 ± 96.71 | 772.57 ± 113.98 |
|  | 3-methoxyphenylpropionic acid | 15.91 ± 47.73 | 14.56 ± 43.67 | 16.33 ± 49 | 14.78 ± 44.33 | 13.38 ± 40.13 | 14.69 ± 44.07 |  | 23.56 ± 70.67 | 23.78 ± 71.33 | 22.44 ± 67.33 | 22.22 ± 66.67 | 30.25 ± 85.56 | 33.43 ± 88.44 |
|  | 4-hydroxyphenylpropionic acid | 0 ± 0 | 0 ± 0 | 0 ± 0 | 0 ± 0 | 0 ± 0 | 0 ± 0 |  | 0 ± 0 | 0 ± 0 | 0 ± 0 | 0 ± 0 | 0 ± 0 | 0 ± 0 |
|  | phenylpropionic acid | 88.69 ± 149.62 | 135.11 ± 170.8 | 166.69 ± 145.72 | 109.02 ± 145.6 | 37.56 ± 112.67 | 30.22 ± 90.67 |  | 88.67 ± 178.57 | 109.33 ± 170.65 | 102.89 ± 157.51 | 91.11 ± 138.85 | 84.25 ± 116.63 | 93.43 ± 116.87 |
| **Phenypyruvic Acids** | 4-hydroxyphenylpyruvic acid | 136555.56 ± 34973.1 | 136688.89 ± 27032.59 | 136466.67 ± 40651.32 | 118866.67 ± 26520.37 | 127955.56 ± 32722.59 | 125622.22 ± 20404.77 |  | 123222.22 ± 27583.59 | 123377.78 ± 23170.55 | 128088.89 ± 28453.14 | 136266.67 ± 30491.15 | 131400 ± 37108.95 | 139857.14 ± 40040.6 |
|  | phenylpyruvic acid | 12904.44 ± 3122.56 | 12437.78 ± 2477.27 | 12373.33 ± 2826.71 | 12682.22 ± 2639.46 | 12835.56 ± 3820.74 | 12717.78 ± 2823.07 |  | 11520 ± 2325.19 | 12120 ± 2593.57 | 12137.78 ± 2674.42 | 12240 ± 2496.38 | 12030 ± 3261.83 | 11522.86 ± 3098.48 |
|  |  |  |  |  |  |  |  |  |  |  |  |  |  |  |

Bold underlined values indicate significant differences in scores when compared to the baseline.

**Supplementary Table 6.** Plasma metabolites following the chronic consumption (over 3 days) of the control and bean hull bread rolls.

|  |  | **Control** | **Broad Bean Bread** |
| --- | --- | --- | --- |
| **Acetophenones** | 3,4,5-trimethoxyacetophenone | 0 ± 0 | 0 ± 0 |
|  | 3,4-dimethoxyacetophenone | 0 ± 0 | 0 ± 0 |
|  | 4-hydroxy-3,5-dimethoxyacetophenone | 0 ± 0 | 0 ± 0 |
|  | 4-hydroxy-3-methoxyacetophenone | 5.42 ± 6.45 | 6.45 ± 2.16 |
|  | 4-hydroxyacetophenone | 11.62 ± 10.52 | 10.52 ± 5.52 |
| **Amines** | 5-OHtryptophan | 581.78 ± 572.22 | 572.22 ± 53.89 |
|  | cadaverine | 1134.67 ± 1171.33 | 1171.33 ± 64.98 |
|  | histamine | 28.44 ± 30.36 | 30.36 ± 1.32 |
|  | piperidine | 1096.67 ± 1159.56 | 1159.56 ± 68.51 |
|  | putresine | 24733.33 ± 26088.89 | 26088.89 ± 2181.74 |
|  | pyrollidine | 0 ± 0 | 0 ± 0 |
|  | Spermidine | 4204.44 ± 4562.22 | 4562.22 ± 263.59 |
|  | tyromine | 15415.56 ± 15968.89 | 15968.89 ± 649.44 |
| **Benzaldehydes** | 3,4,5-trihydroxybenzaldehyde | 0 ± 0 | 0 ± 0 |
|  | 3,4,5-trimethoxybenzaldehyde | 0 ± 0 | 0 ± 0 |
|  | protocatachaldehyde | 40.78 ± 40.53 | 40.53 ± 3.84 |
|  | 3,4-dimethoxybenzaldehyde | 0 ± 0 | 0 ± 0 |
|  | syringin | 0 ± 0 | 0 ± 0 |
|  | vanillin | 0 ± 0 | 0 ± 0 |
|  | 3-methoxybenzaldehyde | 0 ± 0 | 0 ± 0 |
|  | p-hydroxybenzaldehyde | 11.95 ± 10.97 | 10.97 ± 2.07 |
|  | isovanillin | 0 ± 0 | 0 ± 0 |
| **Benzenes** | 1,2,3-trihydroxybenzene | 0 ± 0 | 0 ± 0 |
|  | 1,2-hydroxybenzene | 0 ± 0 | 0 ± 0 |
|  | 1,3-hydroxybenzene | 0 ± 0 | 0 ± 0 |
|  | phenol | 0 ± 0 | 0 ± 0 |
| **Benzoic Acids** | 2,3-dihydroxybenzoic acid | 0 ± 0 | 0 ± 0 |
|  | 2,4-dihydroxybenzoic acid | 0 ± 0 | 0 ± 0 |
|  | 2,5-dihydroxybenzoic acid | 0 ± 0 | 0 ± 0 |
|  | 2,6-dihydroxybenzoic acid | 1.74 ± 3.52 | 3.52 ± 5.23 |
|  | salicylic acid | 20.46 ± 29.08 | 29.08 ± 13.72 |
|  | o-anisic acid | 0 ± 0 | 0 ± 0 |
|  | gallic acid | 0 ± 0 | 0 ± 0 |
|  | 3,4-dimethoxybenzoic acid | 12.19 ± 11.81 | 11.81 ± 3.98 |
|  | 3,5-dihydroxybenzoic acid | 0 ± 0 | 0 ± 0 |
|  | syringic acid | 123.76 ± 124.09 | 124.09 ± 7.83 |
|  | m-hydroxybenzoic acid | 0 ± 0 | 0 ± 0 |
|  | vanillic acid | 1342.67 ± 1309.11 | 1309.11 ± 73.99 |
|  | m-anisic acid | 0 ± 0 | 0 ± 0 |
|  | p-hydroxybenzoic acid | 2011.11 ± 1840.67 | 1840.67 ± 643.5 |
|  | p-anisic acid | 0 ± 0 | 0 ± 0 |
|  | benzoic acid | 1167.56 ± 1144.22 | 1144.22 ± 101.83 |
|  | protocatechuic acid | 2511.11 ± 2504.44 | 2504.44 ± 204.97 |
| **Cinnamic Acids** | o-coumaric acid | 0 ± 0 | 0 ± 0 |
|  | 3,4-dimethoxycinnamic acid | 28.89 ± 25.76 | 25.76 ± 7.93 |
|  | 3,4,5-trimethoxycinnamic acid | 0 ± 0 | 0 ± 0 |
|  | caffeic acid | 30.84 ± 31.58 | 31.58 ± 2.83 |
|  | m-coumaric acid | 0 ± 0 | 0 ± 0 |
|  | ferulic acid | 31.42 ± 31.04 | 31.04 ± 5.84 |
|  | 3-methoxycinnamic acid | 0 ± 0 | 0 ± 0 |
|  | p-coumaric acid | 0 ± 0 | 0 ± 0 |
|  | 4-methoxycinnamic acid | 0 ± 0 | 0 ± 0 |
|  | cinnamic acid | 0 ± 0 | 0 ± 0 |
|  | sinapic acid | 21.53 ± 18.71 | 18.71 ± 3.25 |
| **Flavanoids/Coumarins/Isoflavonoids** |  |  |  |
|  | 7,8-dihydroxy-6-methyl coumarin | 0.95 ± 0.52 | 0.52 ± 1.9 |
|  | 8-methylpsoralen | 2.64 ± 2.54 | 2.54 ± 0.57 |
|  | Apigenin | 1123.56 ± 1197.11 | 1197.11 ± 109.48 |
|  | Bergapten | 0 ± 0 | 0 ± 0 |
|  | Biochanin A | 87.36 ± 89.33 | 89.33 ± 5.09 |
|  | Catechin | 0 ± 0 | 0 ± 0 |
|  | Coumarin | 0 ± 0 | 0 ± 0 |
|  | Coumesterol | 0 ± 0 | 0 ± 0 |
|  | Daidzein | 17.84 ± 17.5 | 17.5 ± 1.38 |
|  | Didymin | 0 ± 0 | 0 ± 0 |
|  | Epicatechin | 0 ± 0 | 0 ± 0 |
|  | Epigallocatechin | 0 ± 0 | 0 ± 0 |
|  | Epigallocatechin Gallate | 0 ± 0 | 0 ± 0 |
|  | Eriocitrin | 0 ± 0 | 0 ± 0 |
|  | Fisetin | 0 ± 0 | 0 ± 0 |
|  | Formononetin | 161.98 ± 166.22 | 166.22 ± 12.05 |
|  | Galangin | 0 ± 0 | 0 ± 0 |
|  | Gallocatechin | 0 ± 0 | 0 ± 0 |
|  | Genstein | 131.4 ± 134.27 | 134.27 ± 11.21 |
|  | Glycitein | 6.08 ± 6.33 | 6.33 ± 0.96 |
|  | Gossypin | 0 ± 0 | 0 ± 0 |
|  | Hesperidin | 0 ± 0 | 0 ± 0 |
|  | Hesperitin | 69.31 ± 68.73 | 68.73 ± 5.61 |
|  | Hyperoside | 0 ± 0 | 0 ± 0 |
|  | Imperatorin | 1.05 ± 0.64 | 0.64 ± 1.58 |
|  | Isoliquiritigenin | 0 ± 0 | 0 ± 0 |
|  | Isorhamnetin | 996.44 ± 1055.56 | 1055.56 ± 78.52 |
|  | Kaempferol | 462.89 ± 490.67 | 490.67 ± 43.93 |
|  | Lariciresinol | 0 ± 0 | 0 ± 0 |
|  | Luteolin | 3442.22 ± 3417.78 | 3417.78 ± 460.59 |
|  | Luteolinidin | 24.71 ± 22.12 | 22.12 ± 12.28 |
|  | Morin | 66.49 ± 68.71 | 68.71 ± 6.58 |
|  | Myricetin | 0 ± 0 | 0 ± 0 |
|  | Naringenin | 49.91 ± 49.07 | 49.07 ± 3.47 |
|  | Naringin | 0 ± 0 | 0 ± 0 |
|  | Neoeriocitrin | 0 ± 0 | 0 ± 0 |
|  | Neohesperidin | 0 ± 0 | 0 ± 0 |
|  | Phloretin | 0 ± 0 | 0 ± 0 |
|  | Phloridzin | 0 ± 0 | 0 ± 0 |
|  | Pinoresinol | 507.56 ± 470.89 | 470.89 ± 60.43 |
|  | Poncirin | 0 ± 0 | 0 ± 0 |
|  | Psoralen | 3.95 ± 4.1 | 4.1 ± 0.88 |
|  | Quercetin | 548 ± 559.11 | 559.11 ± 88.13 |
|  | Quercetin-3-Glucoside | 0 ± 0 | 0 ± 0 |
|  | Quercitrin | 0 ± 0 | 0 ± 0 |
|  | Scopoletin | 85.42 ± 83.36 | 83.36 ± 7.68 |
|  | Rutin | 0 ± 0 | 0 ± 0 |
|  |  |  |  |
|  | Tangeretin | 2.42 ± 2.69 | 2.69 ± 1.42 |
|  | Taxifolin | 0 ± 0 | 0 ± 0 |
|  | Umbelliferone | 30.96 ± 30.67 | 30.67 ± 1.74 |
|  | Vitexin | 471.56 ± 453.78 | 453.78 ± 21.65 |
|  |  |  |  |
| **Indoles** | indole-3-acetic acid | 636.89 ± 606 | 606 ± 80.78 |
|  | indole-3-carboxylic acid | 42 ± 39.71 | 39.71 ± 4.92 |
|  | indole-3-propionic acid acid | 190.31 ± 281.6 | 281.6 ± 49.52 |
|  | indole-3-carbinol | 0 ± 0 | 0 ± 0 |
|  | indole-3-pyruvic acid | 0 ± 0 | 0 ± 0 |
|  | indoe-3-lactic acid | 182.22 ± 179.87 | 179.87 ± 25.66 |
|  | indole-3-methyl | 0 ± 0 | 0 ± 0 |
|  | indole | 0 ± 0 | 0 ± 0 |
|  | 3-Indoleacetonitrile | 0 ± 0 | 0 ± 0 |
|  |  |  |  |
|  | I3-carboxaldehyde | 0 ± 0 | 0 ± 0 |
|  | I-acrylic | 0 ± 0 | 0 ± 0 |
| **Mandelic Acids** | 3,4-dihydroxymandelic acid | 0 ± 0 | 0 ± 0 |
|  | 3-hydroxymandelic acid | 0 ± 0 | 0 ± 0 |
|  | 4-hydroxy-3-methoxymandelic acid | 51.33 ± 50.93 | 50.93 ± 5.24 |
|  | 4-hydroxymandelic acid | 426.89 ± 426.89 | 426.89 ± 16.19 |
|  | mandelic acid | 2218.89 ± 2143.78 | 2143.78 ± 189.63 |
| **Phenolic Dimers and lignans** | ellagic acid | 0 ± 0 | 0 ± 0 |
|  | ferulic dimer (5-5 linked) | 0 ± 0 | 0 ± 0 |
|  | ferulic dimer (8-5 linked) | 26.58 ± 24.96 | 24.96 ± 2.39 |
|  | ferulic dimer (8-8 linked) | 0 ± 0 | 0 ± 0 |
|  | reservatrol | 0 ± 0 | 0 ± 0 |
|  | Secoisolariciresinol | 67.67 ± 64.09 | 64.09 ± 7.36 |
|  | Syringaresinol | 1949.56 ± 1751.11 | 1751.11 ± 163.17 |
|  | Matairesinol | 6.45 ± 5.92 | 5.92 ± 7.83 |
|  | Hydroxymatairesinol | 0 ± 0 | 0 ± 0 |
|  | Hydrogenated Ferulic Dimer H5-5 | 9.92 ± 7.98 | 7.98 ± 5.79 |
|  | Enterlactone | 24.38 ± 20.1 | 20.1 ± 11.87 |
|  | Enterodiol | 0 ± 0 | 0 ± 0 |
|  | Equol | 0 ± 0 | 0 ± 0 |
|  | ethylferulate | 0 ± 0 | 0 ± 0 |
| **Phenolics Others** | quinadilic acid | 4.1 ± 3.44 | 3.44 ± 5.12 |
|  | 0-hydroxyhippuric acid | 1.33 ± 2.16 | 2.16 ± 2.09 |
|  | coniferyl alcohol | 0 ± 0 | 0 ± 0 |
|  | 4-ethylphenol | 0 ± 0 | 0 ± 0 |
|  | 4-methylcatechol | 1.82 ± 3.18 | 3.18 ± 5.47 |
|  | anthranilic acid | 6.32 ± 3.11 | 3.11 ± 6.33 |
|  | p-cresol | 0 ± 0 | 0 ± 0 |
|  | chlorogenic acid | 0 ± 0 | 0 ± 0 |
|  | tyrosol | 0 ± 0 | 0 ± 0 |
|  | Hydroxytyrosol | 0 ± 0 | 0 ± 0 |
|  | Caffeine | 366.15 ± 218 | 218 ± 225.09 |
|  | Dopamine | 27.62 ± 28.89 | 28.89 ± 2.25 |
|  | Kynurenic acid | 1169.33 ± 1199.33 | 1199.33 ± 56.96 |
|  | Melatonin | 0 ± 0 | 0 ± 0 |
|  | Niacin | 340 ± 350.89 | 350.89 ± 26.53 |
|  | Serotonin | 3.75 ± 5.31 | 5.31 ± 3.57 |
|  | Spermine | 197.69 ± 174.51 | 174.51 ± 20.84 |
| **Phenylacetic acids** | 3,4-dihydroxyphenylacetic acid | 0 ± 0 | 0 ± 0 |
|  | 3-hydroxyphenylacetic acid | 5.9 ± 9.72 | 9.72 ± 6.56 |
|  | 4-hydroxy-3-methoxyphenylacetic acid | 0 ± 0 | 0 ± 0 |
|  | 4-hydroxyphenylacetic acid | 773.56 ± 762.22 | 762.22 ± 122.58 |
|  | 4-methoxyphenylacetic acid | 9.87 ± 8.79 | 8.79 ± 1.71 |
|  | phenylacetic acid | 8080 ± 7884.44 | 7884.44 ± 553.62 |
| **Phenyllactic Acids** | 4-hydroxyphenyllactic acid | 244.96 ± 245.18 | 245.18 ± 47.04 |
|  | phenyllactic acid | 7448.89 ± 7055.56 | 7055.56 ± 412.45 |
| **Phenylpropionic acids** | 2-hydroxyphenylpropionic acid | 7.74 ± 9.77 | 9.77 ± 3.22 |
|  | 3,4-dihydroxyphenylpropionic acid | 39.58 ± 26.93 | 26.93 ± 47.09 |
|  | 3-hydroxyphenylpropionic acid | 3.89 ± 23.87 | 23.87 ± 11.67 |
|  | 3-methoxyphenylpropionic acid | 0 ± 0 | 0 ± 0 |
|  | 4-hydroxy-3-methoxyphenylpropionic acid | 869.56 ± 817.78 | 817.78 ± 45.67 |
|  | 4-hydroxyphenylpropionic acid | 0 ± 0 | 0 ± 0 |
|  | phenylpropionic acid | 45.58 ± 93.56 | 93.56 ± 90.51 |
| **Phenypyruvic Acids** | 4-hydroxyphenylpyruvic acid | 81488.89 ± 90266.67 | 90266.67 ± 28086.85 |
|  | phenylpyruvic acid | 18833.33 ± 17766.67 | 17766.67 ± 1168.46 |
| **Bile acids** | Deoxycholic acid | 68.15 ± 83.79 | 83.79 ± 45.78 |
|  | b-Muricholic Acid | 0 ± 0 | 0 ± 0 |
|  | Tauromuricholic Acid | 1.12 ± 1.83 | 1.83 ± 3.35 |
|  | Ursodeoxycholic Acid | 2.66 ± 0 | 0 ± 7.97 |
|  | Cholic acid | 22.35 ± 24.18 | 24.18 ± 37.73 |
|  | Chenodeoxycholic acid | 7.81 ± 22.74 | 22.74 ± 23.42 |
|  | Glycocholic acid | 31.98 ± 45.79 | 45.79 ± 28.95 |
|  | Glycochenodeoxycholic acid | 148.27 ± 177.96 | 177.96 ± 90.99 |
|  | Glycodeoxycholic | 66.59 ± 124.38 | 124.38 ± 49.38 |
|  | Glycoursodeoxycholic acid | 17.36 ± 15.83 | 15.83 ± 19.75 |
|  | Taurocholic acid | 3.03 ± 2.99 | 2.99 ± 9.09 |
|  | Taurochenodeoxycholic acid | 35.75 ± 42.84 | 42.84 ± 32.85 |
|  | Taurodeoxycholic acid | 12.36 ± 16.06 | 16.06 ± 8.22 |
|  | Tauroursodeoxycholic acid | 0 ± 0 | 0 ± 0 |


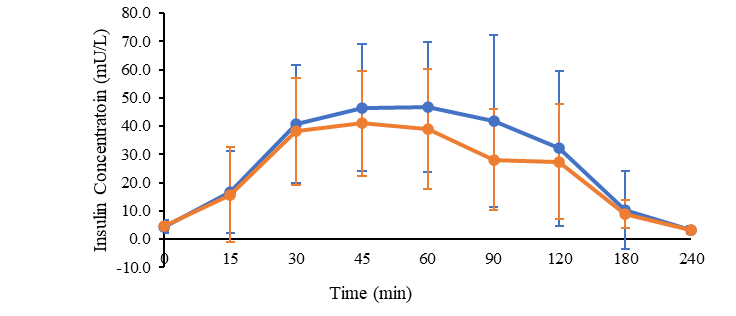


*

**Supplementary Figure 3**. Postprandial insulin concentration over 4 hours following the bean hull (orange) and control (blue) bread roll on day 1.

**Supplementary Figure 4**. Blood glucose levels using the continuous glucose monitor for 4 hours following the bean hull (orange) and control (blue) bread rolls.

**Supplementary Table 7.** Lipid profile over 4 hours (day 1) following the acute consumption of bean hull and control bread rolls. No significant difference was observed between the two interventions.

|  | **Fasted** | **1 h** | **2 h** | **3 h** |
| --- | --- | --- | --- | --- |
| **Control bread roll** | | | | |
| Total cholesterol | 5.4 ± 1.2 | 5.3 ± 1.2 | 5.2 ± 1.1 | 5.3 ± 1.3 |
| HDL | 1.5 ± 0.5 | 1.4 ± 0.5 | 1.4 ± 0.5 | 1.5 ± 0.6 |
| LDL | 3 ± 0.8 | 2.9 ± 0.8 | 2.9 ± 0.7 | 2.9 ± 0.8 |
| Triglycerides | 1.3 ± 0.8 | 1.2 ± 0.7 | 1.2 ± 0.8 | 1.3 ± 0.8 |
| **Bean hull bread roll** | | | | |
| Total cholesterol | 5.3 ± 0.6 | 5.2 ± 0.7 | 5.3 ± 0.7 | 5.1 ± 0.5 |
| HDL | 1.4 ± 0.5 | 1.4 ± 0.5 | 1.4 ± 0.5 | 1.3 ± 0.5 |
| LDL | 3 ± 0.4 | 2.9 ± 0.4 | 2.9 ± 0.4 | 2.9 ± 0.4 |
| Triglycerides | 1.2 ± 0.8 | 1.2 ± 0.6 | 1.2 ± 0.6 | 1.3 ± 0.6 |

HDL: High density lipoprotein; LDL: Low density lipoprotein

| **Intervention (Day 4)** | **Total Cholesterol (mmol/L)** | **HDL (mmol/L)** | **LDL (mmol/L)** | **Triglycerides (mmol/L)** |
| --- | --- | --- | --- | --- |
| Control bread rolls | 5.5 ± 1.3 | 1.5 ± 0.5 | 3.0 ± 0.8 | 1.1 ± 0.5 |
| Bean Hull bread rolls | 5.3 ± 0.8 | 1.4 ± 0.6 | 3.0 ± 0.5 | 1.0 ± 0.2 |

**Supplementary Table 8.** Lipid profile on day 4 following the chronic consumption of bean hull and control bread rolls. No significant difference was observed between the two interventions.

HDL: High density lipoprotein; LDL: Low density lipoprotein

**Supplementary Figure 5**. Interindividual variation in faecal metabolites measured. The numbers depict the individual volunteers’ numbers. Where NS-nonsignificant.

**Supplementary Figure 6**. Mean ± SEM average hunger, fullness, desire and quantity as assessed with the visual analogue scale following the acute consumption of the bean hull (orange) and control (blue) bread rolls. No significant differences were found between the test meals.
